# The 12-Year Experience of the Hungarian Pancreatic Study Group

**DOI:** 10.3390/jcm14041362

**Published:** 2025-02-18

**Authors:** Andrea Szentesi, Péter Hegyi, on behalf of the Hungarian Pancreatic Study Group

**Affiliations:** 1 Institute for Translational Medicine, Medical School, University of Pécs, 7624 Pécs, Hungary; szentesiai@gmail.com; 2 Institute of Pancreatic Diseases, Semmelweis University, 1083 Budapest, Hungary; 3 Centre for Translational Medicine, Semmelweis University, 1085 Budapest, Hungary; 4 Translational Pancreatology Research Group, Interdisciplinary Centre of Excellence for Research Development and Innovation, University of Szeged, 6720 Szeged, Hungary

**Keywords:** acute pancreatitis, chronic pancreatitis, pancreatic cancer, study group

## Abstract

The Hungarian Pancreatic Study Group (HPSG) was established with the aim of advancing pancreatology. Our summary outlines the methodologies, key results, and future directions of the HPSG. Methodological elements included, the formation of strategic national and international collaborations, the establishment of patient registries and biobanks, and a strong focus on education and guideline development. Key results encompassed, pioneering research on pancreatic ductal function and the role of cystic fibrosis transmembrane conductance regulator (CFTR) in inflammation, significant advancements in understanding acute and chronic pancreatitis, and the execution of numerous clinical trials to explore new therapeutic approaches. Despite challenges, such as securing funding and translating research into clinical practice, the HPSG’s commitment to patient care and scientific innovation has been unwavering. The group aims to deepen research into pancreatic cancer and chronic pancreatitis, conduct more randomized controlled trials (RCTs), and expand its efforts internationally by involving global staff and patients. The authors hope that this summary inspires others to undertake similar initiatives and contribute to the global advancement of medical research and patient care in pancreatology.

## 1. Importance of a Research Network

The incidence of pancreatic diseases has increased substantially in recent decades [1,2]. Furthermore, acute pancreatitis (AP) is the most frequent gastrointestinal disease requiring hospitalization, and the early diagnosis of chronic pancreatitis (CP) and pancreatic cancer (PC) are still a great challenge [3,4,5]. The majority of PC patients are diagnosed with locally advanced or metastatic disease, and only a minority are at a resectable stage and the five-year survival is less than 10% [6]. Although there is a great need for solutions, pancreatitis research, especially in acute pancreatitis, had been decreasing between 1965 and 2015 [7]. Public and private financing did not support improvement in this field either [8,9]. Large collaborative efforts were warranted for quality research in pancreatology and also to utilize the power of large sample sizes and to secure the generalizability of the research results.

The European Pancreatic Club (EPC) was established in 1965 [10], the Japan Pancreatic Research Association in 1969 [11], the American Pancreatic Association in 1978 [12], and the Pancreatic Society of Great Britain and Ireland in 1975. Several western European (German, Italian, etc.) national pancreatic associations were born in the 1980s. In the 2000s, some central–eastern European countries had their own societies (Romania, Poland, etc.) and the Indian Pancreas Club was also born. Although the EPC made efforts to involve eastern European countries [10] and had a great role in supporting collaborations, knowledge sharing, and the training of researchers, there was no international visibility of the central-eastern European pancreatic research efforts.

In Hungary, Vince Varró, the key figure of Hungarian gastroenterology [13], recognized the need for specialists who primarily treat pancreatic patients and perform research in the field of pancreatology as early as the 1970s. This was the period when the history of Hungarian pancreatology was born. In the 1980s, Miklós Papp (Budapest) and Ákos Pap (Szeged) ensured the visibility of Hungarian pancreatology and the first Hungarian conference was held in Budapest in 1988. During these periods, researchers committed to Hungarian pancreatology went mainly to western Europe, mostly to Marseille [14,15], to do research and to try to establish their knowledge. However, the feudalistic and economic conditions of the eastern and central European countries did not allow the establishment of a center of excellence. Between 1980 and 2000, the Hungarian conditions were perfect for young people to learn the basics of pancreatic research in their early years (Péter Hegyi’s first publication was published in 1994, as a fourth-year medical student), but they did not have the opportunity to break out. The really big change came in 2001, when Péter Hegyi managed to win a Wellcome Trust Initiative Research Development Award, which gave him the opportunity to set up a well-equipped laboratory in Szeged. His enthusiasm and commitment made it attractive for young people to join pancreatic research, and within a short period of time the Szeged lab has made a fundamental contribution to the understanding of the pathomechanism of AP. Today, Péter Hegyi and his colleagues are associated in the international community with our understanding of pancreatic ductal function and the role of CFTR in inflammation. They discovered that (i) pancreatitis-inducing factors such as bile acids, fatty acids, and ethanol dose-dependently deteriorate pancreatic ductal secretion via mitochondrial damage and calcium overload [16,17], (ii) the function of CFTR is strongly inhibited by alcohol and fatty acids [18], and (iii) the restoration of ductal function by ATP or MPTP inhibitors decrease the severity of acute pancreatitis [19].

There was a burning desire among them to translate these findings for patients’ benefit, so in 2011 the HPSG was established. Since then, the HPSG initiated seven patient registries and 12 clinical trials, organized 16 conferences, attracted 64 PhD students, built an extensive collaboration network with 20 Hungarian centers and hundreds of foreign centers, and published 156 first and/or last authored papers and contributed to 54 further articles.

Here, we aim to summarize the first 12 years of the HPSG to serve as a model to build a study group and support and boost research and collaborations in pancreatology.

## 2. How to Build a Study Group?

The strategic plan for building the study group included the following: (1) improving patient care, (2) establishing clinical research, (3) attracting young researchers, (4) boosting national and international collaborations, (5) developing interdisciplinary clinical research support, and (6) developing educational materials for patients and healthcare professionals. The most important steps involved in reaching these goals are as follows.

### 2.1. International Experience and Leadership

Between 1999 and 2008, several members of the Szeged working group (Péter Hegyi, Zoltán Rakonczay, and Viktória Venglovecz) spent different periods in the most renowned physiological institutes in England (University of Newcastle upon Tyne and University of Liverpool). At the encouragement of Barry E. Argent and Ole Petersen, a bid was submitted, under the leadership of Péter Hegyi, to organize the 41st meeting of the EPC in 2009. In addition to the two English physiologists, Markus Lerch, the EPC General Secretary at the time, had great confidence in the working group, whose trust and support played a key role in the EPC’s selection of Péter Hegyi as the Society’s 2009 President, who at the age of 37 became the youngest ever President so far.

Learning was another milestone in the creation of the HPSG. Peter Hegyi and his colleagues, after a detailed review of the literature, discovered that the Dutch Pancreatitis Study Group was one of the most active and organized networks in the world and invited them to provide a practical introduction on how to set up a study group. Two of Marco Bruno’s students came and presented the model that the HPSG has adopted.

### 2.2. Attracting Young Researchers

Enthusiastic young researchers were recruited among undergraduate students to join as project students and among graduated professionals with medical or natural science background to join as Doctor of Philosophy (PhD) students. Péter Hegyi, as the chair of the study group, regularly presented the activities, results, and opportunities to undergraduate and graduating students in the different faculties of the university. Also, the experimental team took part in the yearly organized “Night of the Researchers” scientific event to demonstrate the research in practice to the interested.

As a result, 19 experimental and 45 clinical PhD students joined the HPSG in the last 12 years, of which 16 and 21 have already graduated, respectively. As project students, more than 45 people have contributed to the research and had the opportunity to improve their scientific records. It is very important to note that in 56% of the publications, the first author was a HPSG PhD student.

### 2.3. Organization of and Participation in International Conferences

One of the main objectives of the national society is to provide a platform for researchers to meet. Although there were efforts by the EPC to involve eastern European countries, it was important to organize conferences locally and make it possible to meet with both clinical experts and researchers. The 43rd EPC international conference was organized in Szeged in 2009, and it was a great success with more than 400 participants. The “International Research Workshop on Acute Pancreatitis” was hosted by the HPSG in 2011 with more than 200 international participants. Then, the 1st Conference of the HPSG was held in Szeged, Hungary in 2012. From that year on, the HPSG conference is traditionally a special occasion for exchanging pancreatic research news and building a valuable network in the pancreas society, especially supporting the participation of central and rastern European countries. Conferences organized by the HPSG are listed in Table 1.

The HPSG is responsible for the pancreas section of the Hungarian Society of Gastroenterology. Every year, both experimental and clinical research is presented by Hungarian researchers and clinicians.

Hungarian participants of the European Pancreatic Club conference have presented a great number of research projects each year. While 13 abstracts were presented in 2011, Hungary was the biggest community with its 57 abstracts at the conference in 2019 in Bergen (Figure S1). The HPSG was also represented at the conferences of United European Gastroenterology, the American Gastroenterology Association, the American Pancreas Association, and the International Association of Pancreatology.

### 2.4. Initiating Patient Registries and Clinical Trials, Establishing the Biobank and Collaborations

Having an internationally visible experimental pancreatitis research background, the translation of research into clinical relevance was extremely important. In order to collect quality clinical data and biological samples, the HPSG elaborated the research plans for patient registries in pancreatology. Clinical data and biomedical samples specific for acute and chronic pancreatitis and pancreatic cancer were collected prospectively by trained clinical administrators or nurses. To ensure data quality, yearly training was organized for data collection participants and a four-step data quality control system was set up in the electronic data management system.

The HPSG started seven patient registries and 12 clinical trials [20,21,22,23,24,25,26,27,28,29,30,31] to date, and Table 2 shows them in a list. The biobank was also set up for the uniform storage of biological samples for genetic and biochemical investigations. International collaborations started in order to involve partners and multiple centers in the patient registries, also, to provide data and samples for consortium research projects.

The main profile of experimental research was acute pancreatitis; however, with the establishment of clinical data collection also in CP and PC, national and international cooperations were intensively developed to exploit more opportunities in all three research areas.

The HPSG built contacts with local hospitals, and later with international healthcare establishments, to work together in data collection. Almost 20 Hungarian hospitals and hundreds of international hospitals and clinics provided data into the patient registries and clinical surveys [32,33]. Also, the HPSG collaborated with acknowledged pancreas research study groups. Miklós Sahin-Tóth and his research group at Boston University and then at the University of California Los Angeles, and Jonas Rosendahl at the University of Leipzig, then at the Martin-Luther-University Halle-Wittenberg, included Hungarian samples for genetic studies in CP [34,35,36,37,38]. In PC, the HPSG provided samples and clinical data to the Pancreatic Disease Research (PANDoRA) consortium led by Federico Canzian and Daniele Campa for genetic research, resulting in several publications [39,40,41,42]. The HPSG contributors are listed in the Supplementary Materials.

The Acute Pancreatitis Registry has over 4000 cases and provided the basis for 28 cohort analysis. The Chronic Pancreatitis Registry yielded two cohort analysis, 12 genetic studies with HPSG first or last authorships, and a further five contributions to other genetic investigations. The Pancreatic Cancer Registry was the basis of one cohort analysis and contributed to another, while 22 genetic investigations used the HPSG database and samples for multicenter genetic analyses.

The results of the first clinical trial, the Early Achievable Severity Index (EASY), have been published, and all other registry data collection and clinical trials are in progress.

### 2.5. Ensuring the Quality of Conducting and Reporting Research Projects

During the design and reporting of studies we followed the recommended guidelines from Enhancing the Quality and Transparency of Health Research (EQUATOR). In the case of meta-analyses, we used the Preferred Reporting Items for Systematic Reviews and Meta-Analyses (PRISMA) or PRISMA Network Meta-analysis (PRISMA-NMA) guidelines and all meta-analyses were registered in the international database of prospectively registered systematic reviews in health and social care (PROSPERO) in advance. Case reports followed the CARE guideline, while study protocols were developed according to the SPIRIT guideline. All clinical trials were registered in the International Standard Randomized Controlled Trial Number (ISRCTN) Registry or ClinicalTrials.gov. The cohort analyses were completed according to the STROBE guideline. The quality evaluation of these studies, using the Newcastle-Ottawa Scale tool, can be seen in Table S1.

### 2.6. Translational Attitude

After initiating patient registries and clinical trials, the HPSG had to cope with several obstacles, which are as follows: (1) No patient organizations or information sources were available. (2) Evidence-based guidelines for pancreatic diseases were not applied in most departments treating patients with pancreatic diseases. (3) Pancreatic patients were treated in scattered departments, making the patient routes, communication between departments, and the follow-up of patients very difficult. (4) Physicians did not receive education on clinical research methodologies. (5) There were not sufficient personnel for the clinical data administration in the healthcare institutions and there was no informatics, statistical, administrative, or communication support available for clinical research. Therefore, the HPSG had to do the following: (1) Disseminate information to patients and direct the training programs and courses to clinical research administrators, physicians, and medical students. Patients were educated at patient club events and through educational videos and printed materials. Clinical research administrators were trained for quality data and biological sample collection and monitoring. (2) Adapt international guidelines on pancreatic diseases and publish them in Hungarian. (3) Establish centralized patient care facilities in Pécs, Székesfehérvár, and Budapest. (4) Organize post-graduate courses for physicians to support the evidence-based treatment of patients and launch a medical doctor (MD)-PhD program. (5) Facilitate the employment and training of clinical research administrators through the Translational Medicine Foundation and establish the interdisciplinary research support unit, including informatics, statistics and communication, financed by the Medical School, University of Pécs in Pécs, Hungary and European Union grants.

### 2.7. Funding

Winning research grants is essential for research. The research team has been able to win a total of around 10 m Euro in funding. The HPSG is extremely thankful to the Hungarian Scientific Research Fund (OTKA), Wellcome Trust, Hungarian Academy of Sciences, Royal Society, Hungarian Government, European Union Cohesion Funds, DFG, (German Research Foundation), Rosztóczy Foundation, Hirschberg Foundation, and Cystic Fibrosis Trust for their support.

## 3. The HPSG Contribution to Pancreatic Research and Patient Care

The HPSG has achieved numerous milestones over the past twelve years, significantly advancing the field of pancreatology. This section summarizes the key outcomes and contributions of the HPSG. The following results underscore the group’s commitment to fostering young talent, improving patient care, and expanding scientific knowledge in pancreatology.

### 3.1. Evidence-Based Guideline Adaptation and Development

In 2014 the international evidence-based guidelines in acute, chronic, autoimmune, and pediatric pancreatitis and pancreatic cancer were adapted, and these guidelines were published in 2015 in Hungarian [43,44,45,46,47].

As pediatric pancreatitis is underdiagnosed and international guidelines are missing, the HPSG in collaboration with the EPC prepared the draft of the guidelines that were presented, discussed, and accepted by the expert panel at a consensus meeting held during the 49th Meeting of the EPC in July 2017 [48].

The experts in the HPSG were also active participants and contributors in the development of several European guidelines [49,50,51,52,53,54]. Table S2 shows the list of guidelines.

### 3.2. Educational Programs in Pancreatology

The HPSG started with post-graduate courses for clinicians to emphasize the importance of evidence-based medicine (EBM) guidelines. These courses were organized at the meetings of the Hungarian Society of Gastroenterology. Next, clinical research administrators were trained in quality data collection and monitoring and biological sample collection.

A clinical PhD program in pancreatology started in 2014, and several PhD theses were based on the Pancreas Registry data collection, monitoring, and analyses.

In 2016, four PhD students started at the University of Pécs and parallelly, they were involved in patient care at the specialized pancreatic department. They were treating patients and enrolled them into clinical studies. Between 2017 and 2020, altogether 16 students commenced their PhD studies in the area of pancreatic research. Between 2021 and 2023, 25 PhD students started their studies in the PhD–resident hybrid program at Semmelweis University in Budapest.

### 3.3. Organization of Centralized Patient Care and Clinical Research

Patients are the starting and endpoint of the translational cycle [55,56]. Clinical questions arise in healthcare and the final user of scientific research results should be the patients and the community. Quality patient care based on EBM guidelines is needed for quality research. In 2016, the University of Pécs and European grants provided resources for establishing an eight-bed specialized acute pancreatitis department that served as part of a translational model [55]. In 2017, a similar patient care unit started to operate at the Szent György Teaching Hospital of County Fejér in Székesfehérvár. Patients were treated according to EBM guidelines, and they were enrolled in clinical trials and patient registries. Also, efforts were made to turn research results back into patient benefits. As a result, the excess antibiotic use, length of hospital stay, mortality, and the cost of care could all be decreased [57]. In these patient care units, patients were also enrolled in patient clubs. They had access to information concerning their disease and follow-up, like recommended diet or control examinations. Moreover, they could be included in the design of future clinical trials.

In 2021, a unique opportunity was offered by the Semmelweis University, and the Institute of Pancreatic Diseases was launched with the aim of becoming a center of excellence for pancreatic diseases. The department started with 30 beds for pancreatitis patients. The next year, pancreatic cancer patient management started.

### 3.4. Basic Discoveries in the Pathomechanisms of AP

The working group was also the first internationally to start investigating the role of pancreatic ducts in AP. We were the first to show that the deletion of Na^+^/H^+^ exchanger regulatory factor-1 (NHERF-1) impaired the regulation of CFTR and ductal secretion in mouse pancreatic duct cells leading to the increased severity of AP. We could conclude that pancreatic ductal bicarbonate secretion has a protective effect in pancreatitis [58]. Our experiments revealed that toxic substances that induce pancreatitis, such as alcohol, fatty acids, bile acids, and smoking induce toxic calcium signaling, mitochondrial damage, damage of the anion exchanger, and CFTR, and as a result severely inhibit fluid and bicarbonate secretion [16,17]. In pancreatitis, both CFTR expression and function are severely impaired [59].

### 3.5. New Insights into the Therapy of AP

We also tested molecules which can serve therapeutic possibilities in AP. Ursodeoxycholate [60], novel mitochondrial transition pore inhibitor N-methyl-4-isoleucine cyclosporin [61], inhibitors of TRPM2 [62], kynurenic acid and its analog SZR-72 [63], CFTR corrector (VX-661) and potentiator (VX-770) [64], and fentanyl [65] improved the outcome of pancreatitis in different experimental models. We also provided evidence that restorations of the intracellular ATP level improves CFTR function and thus may have therapeutic benefits in AP [19].

### 3.6. Clinically Implacable Research Findings on Diagnosis, Risk Factors and Prognosis in AP

The first cohort analysis of the Acute Pancreatitis Registry data in 2016 characterized acute pancreatitis and investigated the current practice and guideline compliance [66]. After this general characterization, the HPSG organized several investigations into the diagnostics, risk factors, and prognosis of acute pancreatitis. Identifying etiology is still a challenge in 20–25% of cases. According to our analyses, the diagnostic work-up should be improved [67,68,69]. Our systematic search of drug-induced AP (DIAP) cases in the literature, together with the AP Registry as a control, showed that DIAP has worse outcomes than AP of other etiologies in a dose-dependent manner [70,71].

Age, comorbidities [72], alcohol and smoking [73], the components of metabolic syndrome [74,75,76,77,78], especially in combinations, metabolic-associated fatty liver disease [79,80], high glucose level [81], hypoalbuminemia [82], low initial renal function [83], intense and sharp pain [84], the development of disturbance of consciousness [85], and newly developing early pseudocysts [86] proved to be risk factors for a more severe disease outcome in our cohort and meta-analyses. The translational investigation on the association between blood potential of hydrogen (pH) level and the outcome of AP concluded that a lower pH level is associated with higher mortality rates, longer length of hospitalization, and more severe AP, and that pre-existing metabolic acidosis (MA) deteriorates AP and AP further increases the severity of MA [87]. According to our results, inflammatory bowel diseases elevate the risk of developing acute pancreatitis [88], but does not alter the clinical features and the management of AP [89]. According to our cohort analysis, peak C-reactive protein (CRP), on-admission CRP, and CRP measured within 24 h from the onset of pain all failed to predict AP severity or mortality [90]. Further risk factors (social status, diet, perceived stress, physical activity, and sleeping characteristics) are being investigated in the “Lifestyle, prevention and risk of acute pancreatitis” (LIFESPAN) observational clinical trial [24].

### 3.7. Prognostic Tools Supported by Artificial Intelligence (AI) in AP

Besides individual risk factors, prognostic scores and tools are warranted in AP as those at greater risk of an unfavorable outcome need special attention. Our meta-analysis concluded that if a computed tomography (CT) scan is performed, the CT severity index (CTSI) should be used in addition to the other scoring systems [91]. However, most guidelines recommend a CT scan at least 72 h after the symptoms started, therefore predictive tools in the first hours of admission were still missing. The HPSG developed the EASY severity prediction score [92] and a prognostic tool to identify patients with probable necrosis [93]. Both tools can be used within 24 h of hospital admission, require limited parameters, and are supported by a web application that makes clinical use easy.

### 3.8. New Therapeutic Opportunities in AP

Our study group conducted several surveys, cohort and meta-analysis, and developed clinical trials to investigate therapeutic options in AP. Our meta-analysis on the combined use of ulinastatin and somatostatin found a reduction in complication rates and shorter hospital stays compared to somatostatin analog monotherapy [94]. Fluid resuscitation is recommended as early supportive therapy; however, the quantity and type of fluid is still debated. A recent meta-analysis of ours concluded that lactated Ringer’s solution reduced local complications, organ failure, severity, and mortality in AP [95]. We also showed that enteral feeding is beneficial compared to a nil per os diet in mild and moderate AP as well [96]. The ongoing investigation, the “High versus low energy administration in the early phase of acute pancreatitis” (GOULASH) trial will provide information soon on the role of energy supply [26]. In addition, there are many drugs in pancreatitis, such as antibiotics and proton pump inhibitors, which are overused. Our data clearly suggest that these drugs should be applied much less than they are used today [33,97,98]. Concerning endoscopic interventions, first we summarized our experiences [99], followed by a network meta-analysis which concluded that the combined use of indomethacin and hydration is recommended to prevent post-ERCP (post-endoscopic retrograde cholangiopancreatography) pancreatitis [100]. Regarding the management of pancreatic pseudocyst and walled-off necrosis, in a meta-analysis of 25 studies we found that endoscopy and surgery are both preferable over percutaneous intervention and endoscopic treatment is associated with shorter hospitalization [101]. Mental health should also be considered in AP. Brief psychological intervention proved to be effective at reducing alcohol consumption and at preventing the recurrence of alcohol-induced AP in our post hoc analysis of a prospectively collected database [102], and a longitudinal randomized clinical trial was initiated to test the effect of a combined patient education and an alcohol and smoking cessation program on the recurrence of alcohol-induced AP [28].

Hypoalbuminemia and hypocalcemia are often observed in severe pancreatitis in coronavirus disease (COVID)-19, therefore the supplementation of albumin and calcium to avoid fatty acid toxicity may also be considered [103,104].

### 3.9. Outstanding Achievements in the Post-AP Period

In-hospital mortality can be 3–5% in AP, and up to 30–50% in severe cases. We showed clear evidence that almost as many patients die in the first 3 month as during a hospital stay [105]. In a recent cohort analysis, we proved that the proportion of patients developing CP after AP is exponentially and directly associated with the number of AP episodes (1.6%, 5.8%, and 21% with 1, 2, and 3 AP episodes, respectively) [106] and recurrent AP (RAP) with three or more episodes with no pancreatic morphological alterations can be considered as early CP [3].

The ongoing “Observational longitudinal multicenter investigation of acute pancreatitis” (GOULASH Plus) trial aims to understand the development of CP after AP and to find the factors that can protect the pancreas from the development of the disease [27].

### 3.10. Collaborative Efforts of HPSG in AP

The HPSG collaborated in the “Patient-reported outcome scale in AP” (PAN-PROMISE) study to design and validate a patient-reported outcome measure in AP which can be used as an endpoint in clinical trials [107] and in retrospective multicenter cohort analysis, concluding that early infection and early surgery is associated with mortality in infected pancreatic necrosis [108]. We also collaborated in an important genetic study showing that the variants in the *CLDN2-MORC4* and *PRSS1-PRSS2* loci confer susceptibility to acute pancreatitis [109].

### 3.11. Preclinical and Genetic Studies in CP

The HPSG has made significant contributions to understanding the basic, preclinical, and genetic aspects of CP. Miklós Sahin-Tóth, a key member of the HPSG, has led several studies elucidating the role of trypsinogen in CP [38,110,111]. His research identified that the *PRSS1* variant p.L104P exhibits various phenotypic changes that increase the risk for CP [112]. Additionally, the *PRSS1* mutation p.P17T was found to have similar biochemical characteristics to known pathogenic mutations, implicating accelerated N-terminal processing by CTRC as a relevant mechanism in CP [36].

Using human pancreas tissue samples and a mouse model, an experimental investigation concluded that an imbalance in mucus homeostasis might play a significant role in CP development [113]. The laboratory run by Miklós Sahin-Tóth has made several important discoveries related to genetic factors influencing CP risk. Genetic analysis of the bicarbonate-secreting anion exchanger *SLC26A6* showed that these variants do not alter CP risk [114]. Another study demonstrated that variants affecting amino acid position 241 in human chymotrypsin-like elastases 3A and 3B (*CELA3A* and *CELA3B*) are not associated with CP, However, the intronic variant c.643-7G>T in *CELA3B* was significantly underrepresented in alcoholic CP patients, suggesting it might be a protective variant [115].

Further research found that the common promoter variant c.-253T>C in serine peptidase inhibitor Kazal type 1 (*SPINK1*) was associated with CP. Two of the three newly identified *SPINK1* promoter variants exhibited significant functional defects and should be considered potential risk factors for CP [116]. The molecular targets of the ScheBo Pancreatic Elastase 1 Test predominantly measure CELA3B and also detect CELA3A with lower efficiency. Other pancreatic proteinases or genetic variants of the *CELA3* isoforms have no appreciable impact on test performance [117]. A study reported a hereditary pancreatitis family carrying the heterozygous c.311T>C (p.L104P) *PRSS1* variant, which induces misfolding and ER stress, linking it to recurrent acute or chronic pancreatitis [118].

The *PRSS1-PRSS2* haplotype modifies CP risk only in the presence of alcohol consumption [35]. Alcohol increases the expression of all three trypsinogen isoforms while pancreatic trypsin inhibitor expression remains relatively unchanged, making the alcoholic pancreas less protected against premature trypsin activation [35]. The common truncation variant in pancreatic lipase-related protein 2 (*PNLIPRP2*) was found to be expressed poorly and does not alter CP risk [119]. Common *CASR* gene variants do not modify CP risk in a Hungarian cohort and should not be considered genetic risk factors in the clinical setting [120]. A loss-of-function variant in *CELA3B* is associated with non-alcoholic CP, indicating that reduced CELA3B function predisposes one to pancreatitis [121]. Heterozygous loss-of-function *CTRC* variants increase CP risk approximately 3–7 fold, justifying genetic screening for CP patients [122]. Bicarbonate defective *CFTR* variants, except for p.R75Q, increase CP risk about 2–4 fold [123]. Finally, the novel p.K374E variant of *CPA1* causes misfolding-induced hereditary pancreatitis with autosomal dominant inheritance [124].

These studies highlight the significant advances made by the HPSG in understanding the genetic and molecular bases of chronic pancreatitis, contributing to better diagnostic and therapeutic strategies for the disease.

### 3.12. Involvement in the Basic and Clinical Research in CP

The HPSG started to collect CP patients’ clinical data in 2012 and a descriptive cohort analysis of 229 cases found that smoking and alcohol consumption are risk factors for surgical intervention [125]. The analysis has provided a basic understanding of the epidemiology of the disease in Hungary; however, we are still far behind the international research activity in this field.

### 3.13. Understanding the Genetic Alterations in PC

The HPSG has also focused on understanding the genetic alterations associated with PC. One study investigated a common cholecystokinin-B receptor (*CCKBR*) intronic variant in pancreatic adenocarcinoma within a Hungarian cohort. The data indicated that the variant c.811+32C>A in *CCKBR* does not significantly impact pancreatic cancer risk or survival in this cohort [126]. We also showed that mutations in *CFTR* modestly increase the risk of pancreatic cancer, whereas no association was found between *SPINK1* mutations and pancreatic cancer risk [127]. We also investigated the potential of exosomal biomarkers and cell-free deoxyribonucleic acid (DNA) for prognosis evaluation in pancreatic ductal adenocarcinoma (PDAC) [128]. However, the heterogeneity of study methods and the need for uniformization before clinical application were also highlighted [129]. The collaboration between the HPSG and PANDORA has also resulted in the publication of 21 highly significant papers, the details of which extend beyond the scope of this report [130].

### 3.14. New Opportunities in the Clinical Research of PC

One of our most significant projects is the multicenter prospective data collection and analysis of pancreatic cancer cases. In this study, the Hungarian cohort revealed a low frequency of both acute recurrent and chronic pancreatitis, with most patients having histologically proven ductal adenocarcinoma [131]. Biliary stent placement is a routine intervention, with Hungarian endoscopists favoring metal stents over plastic ones. Smoking status and gemcitabine-based chemotherapy were identified as independent predictors for overall survival [131]. A meta-analysis of the risk factors on pancreatic cancer showed that fatty pancreas markedly elevate the risk for PC [132]. Another investigation revealed that preoperative serum carbohydrate antigen 19-9 levels are not a reliable marker of unresectability and should not be solely used in surgical decision-making [133]. A systematic literature review on endoscopic ultrasound-guided ethanol and radiofrequency ablation of pancreatic insulinomas suggested that endoscopic ultrasound-guided ablation is a safe and effective treatment, serving as an alternative to surgical resection in selected cases [134]. Concerning patients’ quality of life (QoL), we showed that psychological intervention improves it in patients with early-stage cancer [135].

### 3.15. Research in Pediatric Pancreatitis

The HPSG has also made significant contributions to research in pediatric pancreatitis. We showed that early enteral nutrition, started within 24–48 h, is beneficial. However, prospective studies and better presentation of the research are crucially needed to achieve a higher level of evidence [136]. An analysis of an international cohort of 2335 patients found that a positive pancreatic family history most likely signifies a genetic background in early childhood [137]. Unfortunately, none of the available scoring systems provide acceptable sensitivity and specificity for predicting which patients will develop a moderate or severe disease course, so further efforts are crucially needed [138]. Two major pediatric studies are still running. The “Pain in the Early Phase of Pediatric Pancreatitis” (PINEAPPLE) Trial’s pre-study protocol outlines how this multinational prospective clinical trial will help recognize acute pancreatitis in children in a highly efficient manner [31]. Whereas, the “Analysis of Pediatric Pancreatitis” (APPLE) Trial’s pre-study protocol describes an important international study tracking earlier (APPLE-R) and ongoing episodes (APPLE-P) of pancreatitis [29].

## 4. Publication of Results

The HPSG has 210 publications to date, of which 156 are first and/or last authorships. The oldest international Acute Pancreatitis Registry served as a basis for 28 cohort studies and one genetic analysis. Altogether, two cohort studies and 17 genetic studies in chronic pancreatitis are based on the Chronic Pancreatitis Registry data and biological samples. The Pancreatic Cancer Registry data and samples were used for one cohort study and 22 genetic analyses. Both CP and PC genetic studies profited from the collaborations with international research groups. Further, 32 experimental, 13 guideline, 40 meta-analyses, 19 reviews, and 22 articles of other types were published by or in collaboration with the HPSG. Table S3 shows the list of publications.

**Figure 1:**
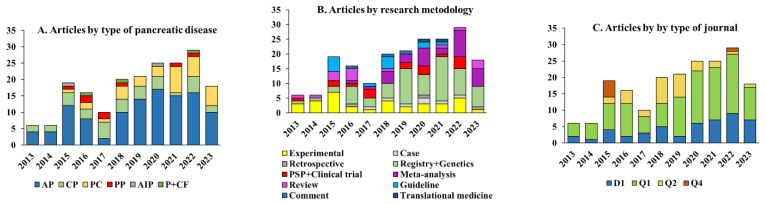
Number of publications by the Hungarian Pancreatic Study Group between 2013 and 2023. AP: acute pancreatitis; CP: chronic pancreatitis; PC: pancreatic cancer; PP: pediatric pancreatitis; AIP: autoimmune pancreatitis; P: pancreatitis miscellaneous; PSP: pre-study protocol.

Most of the research was conducted in acute pancreatitis, but collaborations in chronic pancreatitis and pancreatic cancer genetics resulted in several valuable publications as well (Figure 1A). On top of the experimental research background, clinical research methodologies were used after the foundation of the HPSG. Patient registry cohort analyses, meta-analyses, and clinical trial protocols were published (Figure 1B). The share of D1 and Q1 journals is steadily increasing, especially in the last three years (Figure 1C).

## 5. Major Factors in the Success of a Study Group

The HPSG has made significant strides in pancreatology over the past twelve years. The success of the HPSG can be attributed to several factors. Firstly, they built a robust foundation through experimental research, secondly, they formed strategic national and international collaborations which have been crucial. Partnering with renowned institutions and researchers has not only enhanced the quality of the research but also increased the group’s visibility and credibility on a global scale, and it has improved the generalizability of the results. Additionally, establishing extensive patient registries and biobanks has enabled the HPSG to collect high-quality clinical data and biological samples and has supported the planning and initiation of multicenter clinical trials. Other international examples of high quality multicenter clinical trials also underline the importance of collaborations and quality planning [139,140,141]. This has been essential for translating experimental findings into clinical practice and improving patient outcomes.

The HPSG’s commitment to education and guideline development has also played a critical role. By translating international guidelines into Hungarian and developing specific ones for local needs, they have ensured that healthcare providers have access to the best practices in patient care. Moreover, regularly organizing and participating in international conferences has fostered a strong network of researchers and clinicians.

Despite these achievements, the HPSG has faced several significant challenges. Securing consistent funding and resources for research and clinical trials has been a significant obstacle. While the HPSG has been successful in obtaining grants, ongoing financial support is essential for sustaining and expanding the research activities. Bridging the gap between experimental research and clinical application can also be difficult.

While evaluating the achievements of the HPSG, we also have to consider some limitations. This is a retrospective historical review of the studies organized and conducted by the HPSG. We could not cover the whole spectrum of pancreatic disease diagnosis and treatment and could not go into great details in all studies; however, further information can be found in the original publications. Also, during the journey we learned and continuously improved the quality of research, therefore recent studies definitely represent higher quality than earlier ones.

The journey of the HPSG serves as a powerful testament to what can be achieved with determination and strategic planning. Having a clear vision and unwavering commitment is essential. The HPSG’s dedication to improving pancreatology through research, education, and collaboration has been a driving force behind the success.

The HPSG has proved that with vision, collaboration, innovation, and perseverance, significant progress can be made in medical research and patient care even in a small country. We hope that this success story serves as a model and inspiration for other nations and individuals, proving that with determination and action substantial achievements are within reach.

## 6. Future Plans

The HPSG has ambitious plans to build on its past achievements and address pressing challenges in the field of pancreatology. Moving forward, the group aims to deepen its research into pancreatic cancer and chronic pancreatitis. These conditions remain significant challenges due to their complex pathogenesis and limited treatment options. One of the primary goals is to conduct more randomized controlled trials (RCTs) to explore new therapeutic approaches and improve patient outcomes. The HPSG plans to leverage its established patient registries and biobanks to support these trials, ensuring robust and comprehensive data collection. Additionally, the HPSG is committed to expanding its efforts internationally. This includes not only broadening the scope of research collaborations but also integrating international staff into the patient care teams. The HPSG envisions involving international patients in their studies, offering them access to cutting-edge treatments and the latest research developments. This internationalization strategy will help position the HPSG as a leading global player in pancreatology research and patient care.

The achievements we have reached so far inspire us rather than satisfy us, highlighting the numerous tasks still ahead. We have more questions than answers, and we are committed to serving the field of pancreatology for many decades to come. Our dedication and passion drive us to continue advancing research and improving patient care, ensuring a brighter future for pancreatology.

## Figures and Tables

**Table 1:** Conferences organized or hosted by the HPSG since 2011.

Conferences	Place	Year	International Participants
International Research Workshop on Acute Pancreatitis	Szeged	2011	215
1st Conference of the Hungarian Pancreatic Study Group	Szeged	2012	6
UEG Basic Science Workshop Szeged	Szeged	2012	60
2nd Conference of the Hungarian Pancreatic Study Group	Szeged	2013	4
3rd Conference of the Hungarian Pancreatic Study Group and 9th International Symposium on Alcoholic Liver and Pancreatic Diseases and Cirrhosis	Szeged	2014	63
4th Conference of the Hungarian Pancreatic Study Group	Budapest	2015	24
5th Conference of the Hungarian Pancreatic Study Group	Budapest	2016	6
49th European Pancreatic Club Meeting	Budapest	2017	483
6th Conference of the Hungarian Pancreatic Study Group	Budapest	2017	8
7th Conference of the Hungarian Pancreatic Study Group	Budapest	2018	3
UEG Basic Science Workshop	Pécs	2019	70
8th Conference of the Hungarian Pancreatic Study Group	Budapest	2019	17
9th Conference of the Hungarian Pancreatic Study Group	Pécs (online)	2020	7
10th Conference of the Hungarian Pancreatic Study Group	Budapest	2022	28
11th Conference of the Hungarian Pancreatic Study Group	Budapest	2022	6
12th Conference of the Hungarian Pancreatic Study Group	Budapest	2023	22

**Table 2:** Patient registries and clinical trials initiated by the Hungarian Pancreatic Study Group.

Patient Registries	
Acute Pancreatitis Registry	2012
Chronic Pancreatitis Registry	2012
Pancreatic Cancer Registry	2012
Establishment of the Biobank	2012
Autoimmune Pancreatitis Registry	2015
Walled-Off Pancreas Necrosis Registry	2020
Pancreatic Cystic Neoplasm Registry	2020
Pancreatic Solid Tumor Registry (including Renewed Pancreatic Cancer Registry)	2022
**Clinical Trials**	
EASY: Early achievable severity index	2015
PREPAST: Preventive pancreatic stents in acute biliary pancreatitis	2015
PINEAPPLE: Pain in the early phase of pediatric pancreatitis	2016
APPLE: Analysis of pediatric pancreatitis	2017
GOULASH: High versus low energy administration in the early phase of acute pancreatitis	2017
GOULASH-PLUS: Observational longitudinal multicenter investigation of acute pancreatitis	2019
EMILY: Endoscopic sphincterotomy for delaying cholecystectomy in mild biliary AP	2019
NODES: New onset of diabetes in association with pancreatic ductal adenocarcinoma	2020
ELEFANT: Early elimination of fatty acids in hypertriglyceridemia-induced acute pancreatitis	2020
LIFESPAN: Lifestyle, prevention and risk of acute pancreatitis	2020
REAPPEAR: Recurrent acute pancreatitis prevention by the elimination of alcohol and cigarette smoking	2022
EFFORT: The effect of dietary fat content on the recurrence of pancreatitis	2022

## Data Availability

Not applicable.

## Supplementary Materials

The following supporting information can be downloaded at: https://www.mdpi.com/article/10.3390/jcm14041362/s1, Hungarian Pancreatic Study Group contributors; Figure S1: Number of abstracts by countries at the EPC Meeting in 2019, Bergen, Norway; Table S1: Quality evaluation of HPSG cohort analyses; Table S2: Evidence-based guidelines by the HPSG; Table S3: The list of HPSG publications.

## References

[1] Iannuzzi J.P., King J.A., Leong J.H., Quan J., Windsor J.W., Tanyingoh D., Coward S., Forbes N., Heitman S.J., Shaheen A.-A. (2022). Global incidence of acute pancreatitis is increasing over time: A systematic review and meta-analysis. Gastroenterology.

[2] Pourshams A., Sepanlou S.G., Ikuta K.S., Bisignano C., Safiri S., Roshandel G., Sharif M., Khatibian M., Fitzmaurice C., Nixon M.R. (2019). The global, regional, and national burden of pancreatic cancer and its attributable risk factors in 195 countries and territories, 1990–2017: A systematic analysis for the Global Burden of Disease Study 2017. Lancet Gastroenterol. Hepatol..

[3] Hegyi P.J., Soós A., Tóth E., Ébert A., Venglovecz V., Márta K., Mátrai P., Mikó A., Bajor J., Sarlós P. (2021). Evidence for diagnosis of early chronic pancreatitis after three episodes of acute pancreatitis: A cross-sectional multicentre international study with experimental animal model. Sci. Rep..

[4] Pereira S.P., Oldfield L., Ney A., Hart P.A., Keane M.G., Pandol S.J., Li D., Greenhalf W., Jeon C.Y., Koay E.J. (2020). Early detection of pancreatic cancer. Lancet Gastroenterol. Hepatol..

[5] Singh V.K., Yadav D., Garg P.K. (2019). Diagnosis and management of chronic pancreatitis: A review. JAMA.

[6] Arnold M., Abnet C.C., Neale R.E., Vignat J., Giovannucci E.L., McGlynn K.A., Bray F. (2020). Global burden of 5 major types of gastrointestinal cancer. Gastroenterology.

[7] Szentesi A., Tóth E., Balint E., Fanczal J., Madácsy T., Laczkó D., Ignáth I., Balázs A., Pallagi P., Maléth J. (2016). Analysis of research activity in gastroenterology: Pancreatitis is in real danger. PLoS ONE.

[8] Moses H., Matheson D.H., Cairns-Smith S., George B.P., Palisch C., Dorsey E.R. (2015). The anatomy of medical research: US and international comparisons. JAMA.

[9] Sahin-Toth M. (2017). 2017 American Pancreatic Association Presidential Address Revitalizing Pancreatitis Research. Pancreas.

[10] Goebell H., Ammann R., Creutzfeldt W. (2005). History of the European Pancreatic Club: The First 40 Years 1965–2005. Pancreatology.

[11] Satake K., Takeuchi T. (1998). Brief history of the Japan Pancreas Society. Pancreas.

[12] Tempero M.A. (2019). 2019 American Pancreatic Association Presidential Address: On Our Golden Jubilee. Pancreas.

[13] Varró V. (1996). Zárójelentés.

[14] Pap A., Sarles H. (1986). Effect of atropine on pancreatic secretion in conscious rats. Digestion.

[15] Takacs T., Montet J. (1995). In vitro dissolution of cholesterol biliary stones. Gut.

[16] Venglovecz V., Rakonczay Z., Ozsvari B., Takacs T., Lonovics J., Varro A., Gray M.A., Argent B.E., Hegyi P. (2008). Effects of bile acids on pancreatic ductal bicarbonate secretion in guinea pig. Gut.

[17] Maléth J., Venglovecz V., Rázga Z., Tiszlavicz L., Rakonczay Z., Hegyi P. (2011). Non-conjugated chenodeoxycholate induces severe mitochondrial damage and inhibits bicarbonate transport in pancreatic duct cells. Gut.

[18] Maléth J., Balázs A., Pallagi P., Balla Z., Kui B., Katona M., Judák L., Németh I., Kemény L.V., Rakonczay Z. (2015). Alcohol disrupts levels and function of the cystic fibrosis transmembrane conductance regulator to promote development of pancreatitis. Gastroenterology.

[19] Judák L., Hegyi P., Rakonczay Z., Maléth J., Gray M.A., Venglovecz V. (2014). Ethanol and its non-oxidative metabolites profoundly inhibit CFTR function in pancreatic epithelial cells which is prevented by ATP supplementation. Pflügers Arch. Eur. J. Physiol..

[20] Dubravcsik Z., Madácsy L., Gyökeres T., Vincze Á., Szepes Z., Hegyi P., Hritz I., Szepes A., Group H.P.S. (2015). Preventive pancreatic stents in the management of acute biliary pancreatitis (PREPAST trial): Pre-study protocol for a multicenter, prospective, randomized, interventional, controlled trial. Pancreatology.

[21] Hritz I., Hegyi P. (2015). Early Achievable Severity (EASY) index for simple and accurate expedite risk stratification in acute pancreatitis. J. Gastrointest. Liver Dis..

[22] Illés D., Ivány E., Holzinger G., Kosár K., Adam M.G., Kamlage B., Zsóri G., Tajti M., Svébis M.M., Horváth V. (2020). Protocol: New Onset of DiabetEs in aSsociation with pancreatic ductal adenocarcinoma (NODES Trial): Protocol of a prospective, multicentre observational trial. BMJ Open.

[23] Juhász M.F., Vereczkei Z., Ocskay K., Szakó L., Farkas N., Szakács Z., Zádori N., Wilschanski M., Pandol S.J., Joly F. (2022). The EFFect of dietary fat content on the recurrence of pancreaTitis (EFFORT): Protocol of a multicenter randomized controlled trial. Pancreatology.

[24] Koncz B., Darvasi E., Erdősi D., Szentesi A., Márta K., Erőss B., Pécsi D., Gyöngyi Z., Girán J., Farkas N. (2020). LIFEStyle, Prevention and Risk of Acute PaNcreatitis (LIFESPAN): Protocol of a multicentre and multinational observational case–control study. BMJ Open.

[25] Kucserik L.P., Márta K., Vincze Á., Lázár G., Czakó L., Szentkereszty Z., Papp M., Palatka K., Izbéki F., Altorjay Á. (2019). Endoscopic sphincterotoMy for delayIng choLecystectomy in mild acute biliarY pancreatitis (EMILY study): Protocol of a multicentre randomised clinical trial. BMJ Open.

[26] Márta K., Szabó A.N., Pécsi D., Varjú P., Bajor J., Gódi S., Sarlós P., Mikó A., Papp M., Tornai T. (2017). High versus low energy administration in the early phase of acute pancreatitis (GOULASH trial): Protocol of a multicentre randomised double-blind clinical trial. BMJ Open.

[27] Mikó A., Erőss B., Sarlós P., Hegyi P., Márta K., Pécsi D., Vincze Á., Bódis B., Nemes O., Faluhelyi N. (2019). Observational longitudinal multicentre investigation of acute pancreatitis (GOULASH PLUS): Follow-up of the GOULASH study, protocol. BMJ Open.

[28] Ocskay K., Juhász M.F., Farkas N., Zádori N., Szakó L., Szakács Z., Szentesi A., Erőss B., Miklós E., Zemplényi A. (2022). Recurrent acute pancreatitis prevention by the elimination of alcohol and cigarette smoking (REAPPEAR): Protocol of a randomised controlled trial and a cohort study. BMJ Open.

[29] Párniczky A., Mosztbacher D., Zsoldos F., Tóth A., Lásztity N., Hegyi P., Group H.P.S., Pancreatology T.I.A.O. (2016). Analysis of pediatric pancreatitis (APPLE trial): Pre-study protocol of a multinational prospective clinical trial. Digestion.

[30] Zádori N., Gede N., Antal J., Szentesi A., Alizadeh H., Vincze Á., Izbéki F., Papp M., Czakó L., Varga M. (2020). Early elimination of fatty acids iN hypertriglyceridemia-induced acuTe pancreatitis (ELEFANT trial): Protocol of an open-label, multicenter, adaptive randomized clinical trial. Pancreatology.

[31] Zsoldos F., Párniczky A., Mosztbacher D., Tóth A., Lásztity N., Hegyi P. (2016). Pain in the early phase of pediatric pancreatitis (PINEAPPLE Trial): Pre-study protocol of a multinational prospective clinical trial. Digestion.

[32] Nagy R., Ocskay K., Sipos Z., Szentesi A., Vincze A., Czako L., Izbeki F., Shirinskaya N.V., Poluektov V.L., Zolotov A.N. (2023). Discharge protocol in acute pancreatitis: An international survey and cohort analysis. Sci. Rep..

[33] Párniczky A., Lantos T., Tóth E.M., Szakács Z., Gódi S., Hágendorn R., Illés D., Koncz B., Márta K., Mikó A. (2019). Antibiotic therapy in acute pancreatitis: From global overuse to evidence based recommendations. Pancreatology.

[34] Derikx M.H., Kovacs P., Scholz M., Masson E., Chen J.M., Ruffert C., Lichtner P., Te Morsche R.H., Cavestro G.M., Ferec C. (2015). Polymorphisms at PRSS1-PRSS2 and CLDN2-MORC4 loci associate with alcoholic and non-alcoholic chronic pancreatitis in a European replication study. Gut.

[35] Hegyi E., Tóth A.Z., Vincze Á., Szentesi A., Hegyi P., Sahin-Tóth M. (2020). Alcohol-dependent effect of PRSS1-PRSS2 haplotype in chronic pancreatitis. Gut.

[36] Németh B.C., Szücs Á., Hegyi P., Sahin-Tóth M. (2017). Novel PRSS1 mutation p. P17T validates pathogenic relevance of CTRC-mediated processing of the trypsinogen activation peptide in chronic pancreatitis. Am. J. Gastroenterol..

[37] Rosendahl J., Kirsten H., Hegyi E., Kovacs P., Weiss F.U., Laumen H., Lichtner P., Ruffert C., Chen J.M., Masson E. (2018). Genome-wide association study identifies inversion in the CTRB1-CTRB2 locus to modify risk for alcoholic and non-alcoholic chronic pancreatitis. Gut.

[38] Schnúr A., Beer S., Witt H., Hegyi P., Sahin-Tóth M. (2014). Functional effects of 13 rare PRSS1 variants presumed to cause chronic pancreatitis. Gut.

[39] Campa D., Gentiluomo M., Obazee O., Ballerini A., Vodickova L., Hegyi P., Soucek P., Brenner H., Milanetto A.C., Landi S. (2020). Genome-wide association study identifies an early onset pancreatic cancer risk locus. Int. J. Cancer.

[40] Gentiluomo M., Corradi C., Vanella G., Johansen A.Z., Strobel O., Szentesi A., Milanetto A.C., Hegyi P., Kupcinskas J., Tavano F. (2021). Lack of association of CD44-rs353630 and CHI3L2-rs684559 with pancreatic ductal adenocarcinoma survival. Sci. Rep..

[41] Gentiluomo M.P.M., Bertoncini S., Costello E., Morelli L., Landi S., Milanetto A., Schöttker B., Di Franco G., Ermini S., Scarpa A. (2022). Exploring the Neandertal legacy of pancreatic ductal adenocarcinoma risk in Eurasians. Biol. Res..

[42] Peduzzi G., Archibugi L., Katzke V., Gentiluomo M., Capurso G., Milanetto A.C., Gazouli M., Goetz M., Brenner H., Vermeulen R.C. (2022). Common variability in oestrogen-related genes and pancreatic ductal adenocarcinoma risk in women. Sci. Rep..

[43] Dubravcsik Z., Farkas G., Hegyi P., Hritz I., Kelemen D., Lásztity N., Morvay Z., Oláh A., Pap Á., Párniczky A. (2015). Autoimmune pancreatitis. Evidence based management guidelines of the Hungarian Pancreatic Study Group. Orvosi Hetil..

[44] Hritz I., Czakó L., Dubravcsik Z., Farkas G., Kelemen D., Lásztity N., Morvay Z., Oláh A., Pap Á., Párniczky A. (2015). Acute pancreatitis. Evidence based management guidelines of the Hungarian Pancreatic Study Group. Orvosi Hetil..

[45] Párniczky A., Czakó L., Dubravcsik Z., Farkas G., Hegyi P., Hritz I., Kelemen D., Morvay Z., Oláh A., Pap Á. (2015). Pediatric pancreatitis. Evidence based management guidelines of the Hungarian Pancreatic Study Group. Orvosi Hetil..

[46] Szmola R., Farkas G., Hegyi P., Czakó L., Dubravcsik Z., Hritz I., Kelemen D., Lásztity N., Morvay Z., Oláh A. (2015). Pancreatic cancer. Evidence based management guidelines of the Hungarian Pancreatic Study Group. Orvosi Hetil..

[47] Takács T., Czakó L., Dubravcsik Z., Farkas G., Hegyi P., Hritz I., Kelemen D., Lásztity N., Morvay Z., Oláh A. (2015). Chronic pancreatitis. Evidence based management guidelines of the Hungarian Pancreatic Study Group. Orvosi Hetil..

[48] Párniczky A., Abu-El-Haija M., Husain S., Lowe M., Oracz G., Sahin-Tóth M., Szabó F.K., Uc A., Wilschanski M., Witt H. (2018). EPC/HPSG evidence-based guidelines for the management of pediatric pancreatitis. Pancreatology.

[49] Dominguez-Munoz J.E., Drewes A.M., Lindkvist B., Ewald N., Czakó L., Rosendahl J., Löhr J.M., Löhr M., Besselink M., Mayerle J. (2018). Recommendations from the United European Gastroenterology evidence-based guidelines for the diagnosis and therapy of chronic pancreatitis. Pancreatology.

[50] Greenhalf W., Lévy P., Gress T., Rebours V., Brand R., Pandol S., Chari S., Jørgensen M., Mayerle J., Lerch M. (2020). Working group for the International (IAP–APA–JPS–EPC) Consensus Guidelines for Chronic Pancreatitis. International consensus guidelines on surveillance for pancreatic cancer in chronic pancreatitis. Recommendations from the working group for the international consensus guidelines for chronic pancreatitis in collaboration with the International Association of Pancreatology, the American Pancreatic Association, the Japan Pancreas Society, and European Pancreatic Club. Pancreatology.

[51] Hegyi P., Párniczky A., Lerch M., Sheel A., Rebours V., Forsmark C., Del Chiaro M., Rosendahl J., de-Madaria E., Szücs Á. (2020). Working Group for the International (IAP–APA–JPS–EPC) Consensus Guidelines for Chronic Pancreatitis. International Consensus Guidelines for Risk Factors in Chronic Pancreatitis. Recommendations from the working group for the international consensus guidelines for chronic pancreatitis in collaboration with the International Association of Pancreatology, the American Pancreatic Association, the Japan Pancreas Society, and European Pancreatic Club. Pancreatology.

[52] Löhr J.M., Dominguez-Munoz E., Rosendahl J., Besselink M., Mayerle J., Lerch M.M., Haas S., Akisik F., Kartalis N., Iglesias-Garcia J. (2017). United European Gastroenterology evidence-based guidelines for the diagnosis and therapy of chronic pancreatitis (HaPanEU). United Eur. Gastroenterol. J..

[53] European Study Group on Cystic Tumours of the Pancreas (2018). The European Study Group on Cystic Tumours of the Pancreas. Gut.

[54] Whitcomb D., Shimosegawa T., Chari S., Forsmark C., Frulloni L., Garg P., Hegyi P., Hirooka Y., Irisawa A., Ishikawa T. (2018). Working Group for the International (IAP–APA–JPS–EPC) Consensus Guidelines for Chronic Pancreatitis. International consensus statements on early chronic Pancreatitis. Recommendations from the working group for the international consensus guidelines for chronic pancreatitis in collaboration with The International Association of Pancreatology, American Pancreatic Association, Japan Pancreas Society, PancreasFest Working Group and European Pancreatic Club. Pancreatology.

[55] Hegyi P., Erőss B., Izbéki F., Párniczky A., Szentesi A. (2021). Accelerating the translational medicine cycle: The Academia Europaea pilot. Nat. Med..

[56] Hegyi P., Petersen O.H., Holgate S., Erőss B., Garami A., Szakács Z., Dobszai D., Balaskó M., Kemény L., Peng S. (2020). Academia Europaea position paper on translational medicine: The cycle model for translating scientific results into community benefits. J. Clin. Med..

[57] Gódi S., Erőss B.M., Gyömbér Z., Szentesi A.I., Borbásné Farkas K., Párniczky A., Sarlós P., Bajor J., Czimmer J., Mikó A. (2018). Centralized care for acute pancreatitis significantly improves outcomes. J. Gastrointest. Liver Dis..

[58] Pallagi P., Balla Z., Singh A.K., Dósa S., Iványi B., Kukor Z., Tóth A., Riederer B., Liu Y., Engelhardt R. (2014). The role of pancreatic ductal secretion in protection against acute pancreatitis in mice. Crit. Care Med..

[59] Hegyi P., Petersen O.H. (2013). The exocrine pancreas: The acinar-ductal tango in physiology and pathophysiology. Rev. Physiol. Biochem. Pharmacol..

[60] Katona M., Hegyi P., Kui B., Balla Z., Rakonczay Z., Rázga Z., Tiszlavicz L., Maléth J., Venglovecz V. (2016). A novel, protective role of ursodeoxycholate in bile-induced pancreatic ductal injury. Am. J. Physiol. Gastrointest. Liver Physiol..

[61] Tóth E., Maléth J., Závogyán N., Fanczal J., Grassalkovich A., Erdős R., Pallagi P., Horváth G., Tretter L., Bálint E.R. (2019). Novel mitochondrial transition pore inhibitor N-methyl-4-isoleucine cyclosporin is a new therapeutic option in acute pancreatitis. J. Physiol..

[62] Fanczal J., Pallagi P., Görög M., Diszházi G., Almássy J., Madácsy T., Varga Á., Csernay-Biró P., Katona X., Tóth E. (2020). TRPM2-mediated extracellular Ca^2+^ entry promotes acinar cell necrosis in biliary acute pancreatitis. J. Physiol..

[63] Balla Z., Kormányos E.S., Kui B., Bálint E.R., Fűr G., Orján E.M., Iványi B., Vécsei L., Fülöp F., Varga G. (2021). Kynurenic acid and its analogue SZR-72 ameliorate the severity of experimental acute necrotizing pancreatitis. Front. Immunol..

[64] Fűr G., Bálint E.R., Orján E.M., Balla Z., Kormányos E.S., Czira B., Szűcs A., Kovács D.P., Pallagi P., Maléth J. (2021). Mislocalization of CFTR expression in acute pancreatitis and the beneficial effect of VX-661 + VX-770 treatment on disease severity. J. Physiol..

[65] Bálint E.R., Fűr G., Kui B., Balla Z., Kormányos E.S., Orján E.M., Tóth B., Horváth G., Szűcs E., Benyhe S. (2022). Fentanyl but not morphine or buprenorphine improves the severity of necrotizing acute pancreatitis in rats. Int. J. Mol. Sci..

[66] Párniczky A., Kui B., Szentesi A., Balázs A., Szűcs Á., Mosztbacher D., Czimmer J., Sarlós P., Bajor J., Gódi S. (2016). Prospective, multicentre, nationwide clinical data from 600 cases of acute pancreatitis. PLoS ONE.

[67] Bálint E.R., Fűr G., Kiss L., Németh D.I., Soós A., Hegyi P., Szakács Z., Tinusz B., Varjú P., Vincze Á. (2020). Assessment of the course of acute pancreatitis in the light of aetiology: A systematic review and meta-analysis. Sci. Rep..

[68] Juhász M.F., Ocskay K., Kiss S., Hegyi P., Párniczky A. (2020). Insufficient etiological workup of COVID-19-associated acute pancreatitis: A systematic review. World J. Gastroenterol..

[69] Zádori N., Párniczky A., Szentesi A., Hegyi P. (2020). Insufficient implementation of the IAP/APA guidelines on aetiology in acute pancreatitis: Is there a need for implementation managers in pancreatology?. United Eur. Gastroenterol. J..

[70] Meczker Á., Hanák L., Párniczky A., Szentesi A., Erőss B., Hegyi P., Erdősi D., Mikó A., Zs S., Dobszai D. (2020). Analysis of 1060 cases of drug-induced acute pancreatitis. Gastroenterology.

[71] Meczker Á., Mikó A., Gede N., Szentesi A., Párniczky A., Gódi S., Hegyi P. (2019). Retrospective Matched-Cohort Analysis of Acute Pancreatitis Induced by 5-Aminosalicylic Acid–Derived Drugs. Pancreas.

[72] Szakács Z., Gede N., Pécsi D., Izbéki F., Papp M., Kovács G., Fehér E., Dobszai D., Kui B., Márta K. (2019). Aging and comorbidities in acute pancreatitis II.: A cohort-analysis of 1203 prospectively collected cases. Front. Physiol..

[73] Szentesi A., Farkas N., Sipos Z., Mátrai P., Vincze Á., Izbéki F., Párniczky A., Hegyi P. (2022). Alcohol consumption and smoking dose-dependently and synergistically worsen local pancreas damage. Gut.

[74] Dobszai D., Mátrai P., Gyöngyi Z., Csupor D., Bajor J., Erőss B., Mikó A., Szakó L., Meczker Á., Hágendorn R. (2019). Body-mass index correlates with severity and mortality in acute pancreatitis: A meta-analysis. World J. Gastroenterol..

[75] Kiss L., Fűr G., Mátrai P., Hegyi P., Ivány E., Cazacu I.M., Szabó I., Habon T., Alizadeh H., Gyöngyi Z. (2018). The effect of serum triglyceride concentration on the outcome of acute pancreatitis: Systematic review and meta-analysis. Sci. Rep..

[76] Mikó A., Farkas N., Garami A., Szabó I., Vincze Á., Veres G., Bajor J., Alizadeh H., Rakonczay Z., Vigh É. (2018). Preexisting diabetes elevates risk of local and systemic complications in acute pancreatitis: Systematic review and meta-analysis. Pancreas.

[77] Mosztbacher D., Hanák L., Farkas N., Szentesi A., Mikó A., Bajor J., Sarlós P., Czimmer J., Vincze Á., Hegyi P.J. (2020). Hypertriglyceridemia-induced acute pancreatitis: A prospective, multicenter, international cohort analysis of 716 acute pancreatitis cases. Pancreatology.

[78] Szentesi A., Párniczky A., Vincze Á., Bajor J., Gódi S., Sarlós P., Gede N., Izbéki F., Halász A., Márta K. (2019). Multiple hits in acute pancreatitis: Components of metabolic syndrome synergize each other’s deteriorating effects. Front. Physiol..

[79] Váncsa S., Németh D., Hegyi P., Szakács Z., Hegyi P.J., Pécsi D., Mikó A., Erőss B., Erős A., Pár G. (2020). Fatty liver disease and non-alcoholic fatty liver disease worsen the outcome in acute pancreatitis: A systematic review and meta-analysis. J. Clin. Med..

[80] Váncsa S., Sipos Z., Váradi A., Nagy R., Ocskay K., Juhász F.M., Márta K., Teutsch B., Mikó A., Hegyi P.J. (2023). Metabolic-associated fatty liver disease is associated with acute pancreatitis with more severe course: Post hoc analysis of a prospectively collected international registry. United Eur. Gastroenterol. J..

[81] Nagy A., Juhász M.F., Görbe A., Váradi A., Izbéki F., Vincze Á., Sarlós P., Czimmer J., Szepes Z., Takács T. (2021). Glucose levels show independent and dose-dependent association with worsening acute pancreatitis outcomes: Post-hoc analysis of a prospective, international cohort of 2250 acute pancreatitis cases. Pancreatology.

[82] Ocskay K., Vinkó Z., Németh D., Szabó L., Bajor J., Gódi S., Sarlós P., Czakó L., Izbéki F., Hamvas J. (2021). Hypoalbuminemia affects one third of acute pancreatitis patients and is independently associated with severity and mortality. Sci. Rep..

[83] Tod P., Farkas N., Németh D., Szénási G., Vincze Á., Hágendorn R., Czakó L., Illés D., Izbéki F., Dunás-Varga V. (2021). Initial Renal Function (eGFR) Is a Prognostic Marker of Severe Acute Pancreatitis: A Cohort-Analysis of 1224 Prospectively Collected Cases. Front. Med..

[84] Földi M., Gede N., Kiss S., Vincze Á., Bajor J., Szabó I., Szepes Z., Izbéki F., Gervain J., Hamvas J. (2022). The characteristics and prognostic role of acute abdominal on-admission pain in acute pancreatitis: A prospective cohort analysis of 1432 cases. Eur. J. Pain.

[85] Hágendorn R., Vincze Á., Izbéki F., Gajdán L., Gódi S., Illés A., Sarlós P., Farkas N., Erőss B., Lillik V. (2020). Development of disturbance of consciousness is associated with increased severity in acute pancreatitis. Pancreatology.

[86] Szakó L., Gede N., Váradi A., Tinusz B., Vörhendi N., Mosztbacher D., Vincze Á., Takács T., Czakó L., Izbéki F. (2021). Early occurrence of pseudocysts in acute pancreatitis–a multicenter international cohort analysis of 2275 cases. Pancreatology.

[87] Rumbus Z., Toth E., Poto L., Vincze A., Veres G., Czako L., Olah E., Marta K., Miko A., Rakonczay Z. (2018). Bidirectional relationship between reduced blood pH and acute pancreatitis: A translational study of their noxious combination. Front. Physiol..

[88] Tél B., Stubnya B., Gede N., Varjú P., Kiss Z., Márta K., Hegyi P.J., Garami A., Hegyi E., Szakács Z. (2020). Inflammatory bowel diseases elevate the risk of developing acute pancreatitis: A meta-analysis. Pancreas.

[89] Dohos D., Farkas N., Váradi A., Erőss B., Párniczky A., Szentesi A., Hegyi P., Sarlós P., Czakó L., Boros E. (2022). Inflammatory bowel disease does not alter the clinical features and the management of acute pancreatitis: A prospective, multicentre, exact-matched cohort analysis. Pancreatology.

[90] Farkas N., Hanák L., Mikó A., Bajor J., Sarlós P., Czimmer J., Vincze Á., Gódi S., Pécsi D., Varjú P. (2019). A multicenter, international cohort analysis of 1435 cases to support clinical trial design in acute pancreatitis. Front. Physiol..

[91] Mikó A., Vigh É., Mátrai P., Soos A., Garami A., Balaskó M., Czakó L., Mosdósi B., Sarlós P., Erőss B. (2019). Computed tomography severity index vs. other indices in the prediction of severity and mortality in acute pancreatitis: A predictive accuracy meta-analysis. Front. Physiol..

[92] Kui B., Pintér J., Molontay R., Nagy M., Farkas N., Gede N., Vincze Á., Bajor J., Gódi S., Czimmer J. (2022). EASY-APP: An artificial intelligence model and application for early and easy prediction of severity in acute pancreatitis. Clin. Transl. Med..

[93] Kiss S., Pintér J., Molontay R., Nagy M., Farkas N., Sipos Z., Fehérvári P., Pecze L., Földi M., Vincze Á. (2022). Early prediction of acute necrotizing pancreatitis by artificial intelligence: A prospective cohort-analysis of 2387 cases. Sci. Rep..

[94] Horváth I.L., Bunduc S., Fehérvári P., Váncsa S., Nagy R., Garmaa G., Kleiner D., Hegyi P., Erőss B., Csupor D. (2022). The combination of ulinastatin and somatostatin reduces complication rates in acute pancreatitis: A systematic review and meta-analysis of randomized controlled trials. Sci. Rep..

[95] Ocskay K., Mátrai P., Hegyi P., Párniczky A. (2023). Lactated Ringer’s Solution Reduces Severity, Mortality, Systemic and Local Complications in Acute Pancreatitis: A Systematic Review and Meta-Analysis. Biomedicines.

[96] Márta K., Farkas N., Szabó I., Illés A., Vincze Á., Pár G., Sarlós P., Bajor J., Szűcs Á., Czimmer J. (2016). Meta-analysis of early nutrition: The benefits of enteral feeding compared to a nil per os diet not only in severe, but also in mild and moderate acute pancreatitis. Int. J. Mol. Sci..

[97] Demcsák A., Soós A., Kincses L., Capunge I., Minkov G., Kovacheva-Slavova M., Nakov R., Wu D., Huang W., Xia Q. (2020). Acid suppression therapy, gastrointestinal bleeding and infection in acute pancreatitis—An international cohort study. Pancreatology.

[98] Horváth I.L., Bunduc S., Hankó B., Kleiner D., Demcsák A., Szabó B., Hegyi P., Csupor D. (2023). No evidence for the benefit of PPIs in the treatment of acute pancreatitis: A systematic review and meta-analysis. Sci. Rep..

[99] Halász A., Pécsi D., Farkas N., Izbéki F., Gajdán L., Fejes R., Hamvas J., Takács T., Szepes Z., Czakó L. (2019). Outcomes and timing of endoscopic retrograde cholangiopancreatography for acute biliary pancreatitis. Dig. Liver Dis..

[100] Márta K., Gede N., Szakács Z., Solymár M., Hegyi P.J., Tél B., Erőss B., Vincze Á., Arvanitakis M., Boškoski I. (2021). Combined use of indomethacin and hydration is the best conservative approach for post-ERCP pancreatitis prevention: A network meta-analysis. Pancreatology.

[101] Szakó L., Mátrai P., Hegyi P., Pécsi D., Gyöngyi Z., Csupor D., Bajor J., Erőss B., Mikó A., Szakács Z. (2020). Endoscopic and surgical drainage for pancreatic fluid collections are better than percutaneous drainage: Meta-analysis. Pancreatology.

[102] Nagy R., Ocskay K., Váradi A., Papp M., Vitális Z., Izbéki F., Boros E., Gajdán L., Szentesi A., Erőss B. (2022). In-hospital patient education markedly reduces alcohol consumption after alcohol-induced acute pancreatitis. Nutrients.

[103] El-Kurdi B., Khatua B., Rood C., Snozek C., Cartin-Ceba R., Singh V.P., Kostenko S., Trivedi S., Folmes C., Dykhouse K.M. (2020). Mortality from coronavirus disease 2019 increases with unsaturated fat and may be reduced by early calcium and albumin supplementation. Gastroenterology.

[104] Hegyi P., Szakács Z., Sahin-Tóth M. (2020). Lipotoxicity and cytokine storm in severe acute pancreatitis and COVID-19. Gastroenterology.

[105] Czapári D., Váradi A., Farkas N., Nyári G., Márta K., Váncsa S., Nagy R., Teutsch B., Bunduc S., Erőss B. (2023). Detailed characteristics of post-discharge mortality in acute pancreatitis. Gastroenterology.

[106] Erőss B., Szentesi A., Hegyi P. (2021). Metabolic signature might be an option to identify patients with early CP. Gut.

[107] De-Madaria E., Sánchez-Marin C., Carrillo I., Vege S.S., Chooklin S., Bilyak A., Mejuto R., Mauriz V., Hegyi P., Márta K. (2021). Design and validation of a patient-reported outcome measure scale in acute pancreatitis: The PAN-PROMISE study. Gut.

[108] Moran R.A., Halloran C., Guo Q., Umapathy C., Jalaly N.Y., Jain S., Cowzer D., Robles E.P.C., Quesada-Vázquez N., Szentesi A. (2022). Early infection is an independent risk factor for increased mortality in patients with culture-confirmed infected pancreatic necrosis. Pancreatology.

[109] Weiss F.U., Hesselbarth N., Párniczky A., Mosztbacher D., Lämmerhirt F., Ruffert C., Kovacs P., Beer S., Seltsam K., Griesmann H. (2018). Common variants in the CLDN2-MORC4 and PRSS1-PRSS2 loci confer susceptibility to acute pancreatitis. Pancreatology.

[110] Geisz A., Hegyi P., Sahin-Tóth M. (2013). Robust autoactivation, chymotrypsin C independence and diminished secretion define a subset of hereditary pancreatitis-associated cationic trypsinogen mutants. FEBS J..

[111] Ózsvári B., Hegyi P., Sahin-Tóth M. (2008). The guinea pig pancreas secretes a single trypsinogen isoform, which is defective in autoactivation. Pancreas.

[112] Balázs A., Hegyi P., Sahin-Tóth M. (2016). Pathogenic cellular role of the p. L104P human cationic trypsinogen variant in chronic pancreatitis. Am. J. Physiol. Gastrointest. Liver Physiol..

[113] Balázs A., Balla Z., Kui B., Maléth J., Rakonczay Z., Duerr J., Zhou-Suckow Z., Schatterny J., Sendler M., Mayerle J. (2018). Ductal mucus obstruction and reduced fluid secretion are early defects in chronic pancreatitis. Front. Physiol..

[114] Balázs A., Ruffert C., Hegyi E., Hritz I., Czakó L., Takács T., Szepes Z., Németh B.C., Gervain J., Izbéki F. (2015). Genetic analysis of the bicarbonate secreting anion exchanger SLC26A6 in chronic pancreatitis. Pancreatology.

[115] Párniczky A., Hegyi E., Tóth A.Z., Szücs Á., Szentesi A., Vincze Á., Izbéki F., Németh B.C., Hegyi P., Sahin-Tóth M. (2016). Genetic analysis of human chymotrypsin-like elastases 3A and 3B (CELA3A and CELA3B) to assess the role of complex formation between proelastases and procarboxypeptidases in chronic pancreatitis. Int. J. Mol. Sci..

[116] Hegyi E., Geisz A., Sahin-Tóth M., Derikx M.H., Németh B.C., Balázs A., Hritz I., Izbéki F., Halász A., Párniczky A. (2016). SPINK1 promoter variants in chronic pancreatitis. Pancreas.

[117] Tóth A.Z., Szabó A., Hegyi E., Hegyi P., Sahin-Tóth M. (2017). Detection of human elastase isoforms by the ScheBo Pancreatic Elastase 1 Test. Am. J. Physiol. Gastrointest. Liver Physiol..

[118] Németh B.C., Patai Á.V., Sahin-Tóth M., Hegyi P. (2017). Misfolding cationic trypsinogen variant p. L104P causes hereditary pancreatitis. Gut.

[119] Németh B.C., Pesei Z.G., Hegyi E., Szücs Á., Szentesi A., Hegyi P., Lowe M.E., Sahin-Tóth M. (2018). The common truncation variant in pancreatic lipase related protein 2 (PNLIPRP2) is expressed poorly and does not alter risk for chronic pancreatitis. PLoS ONE.

[120] Takáts A., Berke G., Szentesi A., Farkas G., Izbéki F., Erőss B., Czakó L., Vincze Á., Hegyi P., Sahin-Tóth M. (2021). Common calcium-sensing receptor (CASR) gene variants do not modify risk for chronic pancreatitis in a Hungarian cohort. Pancreatology.

[121] Tóth A., Demcsák A., Zankl F., Oracz G., Unger L.S., Bugert P., Laumen H., Párniczky A., Hegyi P., Rosendahl J. (2022). Loss-of-function variant in chymotrypsin like elastase 3B (CELA3B) is associated with non-alcoholic chronic pancreatitis. Pancreatology.

[122] Takáts A., Berke G., Gede N., Németh B.C., Witt H., Głuszek S., Rygiel A.M., Hegyi P., Sahin-Tóth M., Hegyi E. (2022). Risk of chronic pancreatitis in carriers of loss-of-function CTRC variants: A meta-analysis. PLoS ONE.

[123] Berke G., Gede N., Szadai L., Ocskay K., Hegyi P., Sahin-Tóth M., Hegyi E. (2022). Bicarbonate defective CFTR variants increase risk for chronic pancreatitis: A meta-analysis. PLoS ONE.

[124] Németh B.C., Orekhova A., Zhang W., Nortman S.A., Thompson T., Hegyi P., Abu-El-Haija M. (2020). Novel p. K374E variant of CPA1 causes misfolding-induced hereditary pancreatitis with autosomal dominant inheritance. Gut.

[125] Szücs Á., Marjai T., Szentesi A., Farkas N., Párniczky A., Nagy G., Kui B., Takács T., Czakó L., Szepes Z. (2017). Chronic pancreatitis: Multicentre prospective data collection and analysis by the Hungarian Pancreatic Study Group. PLoS ONE.

[126] Balázs A., Németh B.C., Ördög B., Hegyi E., Hritz I., Czakó L., Czimmer J., Gódi S., Csiszkó A., Rakonczay Z. (2016). A Common CCK-B Receptor Intronic Variant in Pancreatic Adenocarcinoma in a Hungarian Cohort. Pancreas.

[127] Cazacu I.M., Farkas N., Garami A., Balaskó M., Mosdósi B., Alizadeh H., Gyöngyi Z., Rakonczay Z., Vigh É., Habon T. (2018). Pancreatitis-associated genes and pancreatic cancer risk: A systematic review and meta-analysis. Pancreas.

[128] Bunduc S., Gede N., Váncsa S., Lillik V., Kiss S., Juhász M.F., Erőss B., Szakács Z., Gheorghe C., Mikó A. (2022). Exosomes as prognostic biomarkers in pancreatic ductal adenocarcinoma—A systematic review and meta-analysis. Transl. Res..

[129] Bunduc S., Gede N., Váncsa S., Lillik V., Kiss S., Dembrovszky F., Eross B., Szakacs Z., Gheorghe C., Miko A. (2022). Prognostic role of cell-free DNA biomarkers in pancreatic adenocarcinoma: A systematic review and meta–analysis. Crit. Rev. Oncol./Hematol..

[130] Campa D., Gentiluomo M., Stein A., Aoki M.N., Oliverius M., Vodičková L., Jamroziak K., Theodoropoulos G., Pasquali C., Greenhalf W. (2023). The PANcreatic Disease ReseArch (PANDoRA) consortium: Ten years’ experience of association studies to understand the genetic architecture of pancreatic cancer. Crit. Rev. Oncol. Hematol..

[131] Lakatos G., Balazs A., Kui B., Godi S., Szücs Á., Szentesi A., Szentkereszty Z., Szmola R., Kelemen D., Papp R. (2016). Pancreatic cancer: Multicenter prospective data collection and analysis by the Hungarian Pancreatic Study Group. J. Gastrointest. Liver Dis..

[132] Lipp M., Tarján D., Lee J., Zolcsák Á., Szalai E., Teutsch B., Faluhelyi N., Erőss B., Hegyi P., Mikó A. (2023). Fatty pancreas is a risk factor for pancreatic Cancer: A systematic review and Meta-analysis of 2956 patients. Cancers.

[133] Benke M., Farkas N., Hegyi P., Tinusz B., Sarlós P., Erőss B., Vörhendi N., Szakács Z., Szücs Á. (2022). Preoperative Serum Carbohydrate Antigen 19-9 Levels Cannot Predict the Surgical Resectability of Pancreatic Cancer: A Meta-Analysis. Pathol. Oncol. Res..

[134] El Sayed G., Frim L., Franklin J., McCrudden R., Gordon C., Al-Shamma S., Kiss S., Hegyi P., Erőss B., Hegyi P.J. (2021). Endoscopic ultrasound-guided ethanol and radiofrequency ablation of pancreatic insulinomas: A systematic literature review. Ther. Adv. Gastroenterol..

[135] Bognár S.A., Teutsch B., Bunduc S., Veres D.S., Szabó B., Fogarasi B., Zahariev O.J., Vörhendi N., Almog O., Hadani Y. (2024). Psychological intervention improves quality of life in patients with early-stage cancer: A systematic review and meta-analysis of randomized clinical trials. Sci. Rep..

[136] Mosztbacher D., Farkas N., Solymár M., Pár G., Bajor J., Szűcs Á., Czimmer J., Márta K., Mikó A., Rumbus Z. (2017). Restoration of energy level in the early phase of acute pediatric pancreatitis. World J. Gastroenterol..

[137] Juhász M.F., Farkas N., Szentesi A., Wedrychowicz A., Nita A.F., Lásztity N., Tészás A., Tokodi I., Vincze Á., Eross B. (2022). Pancreatic family history does not predict disease progression but connotes alcohol consumption in adolescents and young adults with acute pancreatitis: Analysis of an international cohort of 2,335 patients. Front. Med..

[138] Juhász M.F., Sipos Z., Ocskay K., Hegyi P., Nagy A., Párniczky A. (2022). Admission risk factors and predictors of moderate or severe pediatric acute pancreatitis: A systematic review and meta-analysis. Front. Pediatr..

[139] Latifi D., de Koning W., Vadgama D., Grevers F., van Dam C., Koerkamp B.G., van der Burg S., van Eijck C.H., Mustafa D.A., Dutch Pancreatic Cancer group (2023). The effect of neoadjuvant chemoradiotherapy on the immune profile of pancreatic ductal adenocarcinoma: In-depth analysis of the PREOPANC-1 randomized controlled trial. Pancreatology.

[140] Lee P.J., Culp S., Kamal A., Pothoulakis I., Talukdar R., Kochhar R., Goenka M.K., Gulla A., Gonzales J., Stevens T. (2022). Lactated Ringers Use in the First 24 Hours of Hospitalization is Associated with Improved Outcomes in 999 Acute Pancreatitis Patients. Off. J. Am. Coll. Gastroenterol. ACG.

[141] Yared R.A., Chen C.-C., Vandorpe A., Arvanitakis M., Delhaye M., Viesca M.F.Y., Huberty V., Blero D., Toussaint E., Hittelet A. (2024). Intravenous Hemin, a potential heme oxygenase-1 activator, does not protect from post-ERCP acute pancreatitis in humans: Results of a randomized multicentric multinational placebo-controlled trial. Pancreatology.

