# Supplementary material for: The 12-Year Experience of the Hungarian Pancreatic Study Group
Source: J Clin Med. 2025 Feb 18;14(4):1362. doi: 10.3390/jcm14041362 (PMC11855942; doi:10.3390/jcm14041362)
Supplement: Supplementary file 1 [file jcm-14-01362-s001.zip › jcm-3464742-supplementary.pdf]

The 12-year experience of the Hungarian Pancreatic Study Group

Andrea Szentesi[1], Péter Hegyi[1,2,3,4] , on behalf of the Hungarian Pancreatic Study Group

[1]Institute for Translational Medicine, Medical School, University of Pécs, Pécs, Hungary
[2]Institute of Pancreatic Diseases, Semmelweis University, Budapest, Hungary
[3]Centre for Translational Medicine, Semmelweis University, Budapest, Hungary
[4]Translational Pancreatology Research Group, Interdisciplinary Centre of Excellence for Research Development and Innovation, University of Szeged, Szeged, Hungary

Table of contents

Hungarian Pancreatic Study Group contributors…………………………………………………… 2
Figure S1. Number of abstracts by countries at the EPC Meeting in 2019, Bergen, Norway ….. 19
Table S1. Quality evaluation of HPSG cohort analyses………..……………………………… 20
Table S2. Evidence-based guidelines by the HPSG ……………..……………………………… 21
Table S3. The list of HPSG publications………………………………………………………… 22

Hungarian Pancreatic Study Group contributors

Name	Institute	Role
Hegyi, Péter	Institute of Pancreatic Diseases, Semmelweis University, Budapest, Hungary	author
Szentesi, Andrea	Institute for Translational Medicine, Medical School, University of Pécs, Pécs, Hungary	author
Abada, A	Department of Anaesthesiology and Intensive Therapy, Medical School, University of Pécs, Pécs, Hungary	contributor
Abu-El-Haija, M	Division of Gastroenterology, Hepatology and Nutrition, Cincinnati Children's Hospital Medical Center, Cincinnati, OH, USA	contributor
Adam, MG	Metanomics Health GmbH, Berlin, Germany	contributor
Aghdam, AN	Centre for Translational Medicine, Semmelweis University, Budapest, Hungary	contributor
Alberti, P	Department of Hepato-Pancreato-Biliary and Transplant Surgery, Hospital Universitari Vall d'Hebron, Universitat Autònoma de Barcelona, Barcelona, Spain	contributor
Algül, H	Department of Gastroenterology, TUM, Munich, Germany	contributor
Alhonen, L	Department of Biotechnology and Molecular Medicine, A. I. Virtanen Institute for Molecular Sciences, Biocenter Kuopio, University of Kuopio, Kuopio, Finland	contributor
Alizadeh, H	Division of Hematology, First Department of Internal Medicine, University of Pécs Medical School, Pécs, Hungary	contributor
Almássy, J	Department of Physiology, University of Debrecen, Debrecen, Hungary	contributor
Almog, O	Centre for Translational Medicine, Semmelweis University, Budapest, Hungary	contributor
Al-Shamma, S	The Royal Bournemouth Hospital, University Hospital Dorset, Bournemouth, UK	contributor
Altintas, E	Gastroenterology Department, Faculty of Medicine, Mersin University, Yenisehir/Mersin, Turkey	contributor
Altorjay, Á	Division of Surgery, Fejer County Saint George Teaching Hospital of University of Pécs, Székesfehérvár, Hungary	contributor
Andorka, C	First Department of Pediatrics, Semmelweis University, Faculty of Medicine, Budapest, Hungary	contributor
Antal, J	Institute for Translational Medicine, Medical School, University of Pécs, Pécs, Hungary	contributor
Apadula, L	Pancreas Translational and Clinical Research Center, San Raffaele Scientific Institute, Vita Salute San Raffaele University, Milan, Italy	contributor
Aparicio, D	Surgery Department, Hospital Prof. Ferndo Fonseca, Amadora, Portugal	contributor
Arcidiacono, PG	Pancreato-Biliary Endoscopy and Endosonography Division, Pancreas Translational and Clinical Research Centre, IRCCS San Raffaele Scientific Institute, Milan, Italy	contributor
Argent, BE	Institute for Cell and Molecular Biosciences, University of Newcastle, Newcastle, UK	contributor
Arvanitakis, M	Gastroenterology Department, Gastrointestinal Cancer Unit, Erasme Hospital University, Brussels, Belgium	contributor
Aslan, R	Marmara University Education and Training Hospital, Istanbul, Turkey	contributor
Aveiro, D	Unidade HBP, Serviço de Cirurgia Geral, Centro Hospitalar Tondela-Viseu, Viseu, Portugal	contributor
Aykut, UE	Marmara University Education and Training Hospital, Istanbul, Turkey	contributor
Bagyánszki, M	Department of Physiology, Anatomy, and Neuroscience, Faculty of Science and Informatics, University of Szeged, Szeged, Hungary	contributor
Bajor, J	Department of Gastroenterology, First Department of Medicine, Medical School, University of Pécs, Pécs, Hungary	contributor
Bak, D	Department of Medical Genetics, Institute of Mother and Child, Warsaw, Poland	contributor
Balaban, VD	Department of Surgery, Lithuanian University of Health Sciences, Kaunas, Lithuania	contributor
Balascak, I	Clinic of Obstetrics and Gynecology, Department of Neonatology, University Hospital Motol and 2nd Medical School, Charles University Prague, Prague, Czech Republic	contributor
Balaskó, M	Institute for Translational Medicine, Medical School, University of Pécs, Pécs, Hungary	contributor
Balázs, A	First Department of Medicine, University of Szeged, Szeged, Hungary	contributor
Balci, HR	Gastroenterology Department, Faculty of Medicine, Mersin University, Yenisehir/Mersin, Turkey	contributor
Bálint, ER	Department of Pathophysiology, University of Szeged, Szeged, Hungary	contributor
Balla, Z	Department of Pathophysiology, University of Szeged, Szeged, Hungary	contributor
Baltatzis, M	Manchester Royal Infirmary Hospital, Manchester, United Kingdom	contributor
Bánovčin, P	Clinic of Internal Medicine - Gastroenterology, Jessenius Faculty of Medicine in Martin, Comenius University, Bratislava, Slovakia	contributor
Barauskas, G	Department of Surgery, Lithuanian University of Health Sciences, Kaunas, Lithuania	contributor
Barbu, S	4th Department of Surgery, Iuliu Hatieganu University of Medicine and Pharmacy, Cluj Napoca, Romania	contributor
Bátovský, M	Departement of Gastroenterology Slovak Medical University in Bratislava, Bratislava, Slovakia	contributor
Battyáni, I	Department of Radiology, Medical School, University of Pécs, Pécs, Hungary	contributor
Becker, D	Heart and Vascular Centre, Semmelweis University, Budapest, Hungary	contributor
Becskeházi, E	Department of Pharmacology and Pharmacotherapy, University of Szeged, Szeged, Hungary	contributor
Beer, S	Division of Gastroenterology, Medical Department II, University of Leipzig Medical Center, Leipzig, Germany	contributor

Name	Institute	Role
Bence, M	Department of Molecular and Cell Biology, Boston University, Boston, USA	contributor
Benke, M	First Department of Surgery, Semmelweis University, Budapest, Hungary	contributor
Benyhe, S	Institute of Biochemistry, Biological Research Center, Szeged, Hungary	contributor
Berczi, S	Department of Pathology, University of Szeged, Szeged, Hungary	contributor
Berke, G	Institute for Translational Medicine, Medical School, University of Pécs, Pécs, Hungary	contributor
Berkó, A	Department of Comparative Physiology, University of Szeged, Szeged, Hungary	contributor
Bertilsson, S	Department of Clinical Sciences, Lund University, Lund, Sweden; Department of Health Sciences, Lund University, Lund, Sweden	contributor
Beyer, G	LMU University Hospital, LMU Munich, Munich, Germany	contributor
Bezmarevic, M	Department for Hepatobiliary and Pancreatic Surgery, Clinic for General Surgery, Military Medical Academy, University of Defense, Belgrade, Serbia	contributor
Bhaskar, S	Centre for Cellular and Molecular Biology (CCMB), Council of Scientific and Industrial Research (CSIR), Hyderabad, India	contributor
Bhatia, E	Department of Endocrinology, Sanjay Gandhi Postgraduate Institute of Medical Sciences, Lucknow, India	contributor
Biczó, G	First Department of Medicine, University of Szeged, Szeged, Hungary	contributor
Bideeva, T	Semashko Central Clinical Hospital, Moscow, Russia	contributor
Birkás B	Medical School, Institute of Behavioural Sciences, University of Pécs, Pécs, Hungary	contributor
Blaskó, Á	Institute for Translational Medicine, University of Pécs, Pécs, Hungary	contributor
Blüher, M	Department for Internal Medicine, Neurology and Dermatology, Division of Endocrinology, University of Leipzig, Leipzig, Germany	contributor
Bod, B	Dr. Bugyi István Hospital, Szentes, Hungary	contributor
Boda, K	Department of Medical Informatics, University of Szeged, Szeged, Hungary	contributor
Bódi, N	Department of Physiology, Anatomy, and Neuroscience, Faculty of Science and Informatics, University of Szeged, Szeged, Hungary	contributor
Bódis, B	Division of Endocrinology and Metabolism, First Department of Medicine, University of Pécs, Pécs, Hungary	contributor
Bogár, L	Department of Anaesthesiology and Intensive Therapy, Pecsi Tudomanyegyetem Altalanos Orvostudomanyi Kar, Pecs, Hungary	contributor
Bognár, SA	Institute of Pancreatic Diseases, Semmelweis University, Budapest, Hungary	contributor
Bóna, E	Department of Emergency Medicine, Medical School, University of Pécs, Pécs, Hungary	contributor
Borbély, RZ	Department of Medical Imaging, Bajcsy-Zsilinszky Hospital and Clinic, Budapest, Hungary	contributor
Bordin, DS	A.S. Loginov Moscow Clinical Scientific Center, Moscow, Russia	contributor
Borka, K	Department of Pathology, Forensic and Insurance Medicine, Semmelweis University, Budapest, Hungary	contributor
Boros, E	Department of Internal Medicine, Szent György Teaching Hospital of County Fejér, Székesfehérvár, Hungary	contributor
Boros, I	Department of Biochemistry and Molecular Biology, University of Szeged, Szeged, Hungary	contributor
Boškoski, I	Centre for Endoscopic Research Therapeutics and Training (CERTT), Università Cattolica del Sacro Cuore, Italy	contributor
Braun, M	Institute for Occupational Medicine, GoetheUniversität Frankfurt, Frankfurt, Germany	contributor
Bruno, MJ	Department of Gastroenterology & Hepatology, Erasmus Medical Center, University Medical Center Rotterdam, Rotterdam, the Netherlands	contributor
Budai, BC	Institute of Pancreatic Diseases, Semmelweis University, Budapest, Hungary	contributor
Bugert, P	Institute of Transfusion Medicine and Immunology, Medical Faculty Mannheim, Heidelberg University, German Red Cross Blood Service of Baden-Württemberg, Mannheim, Germany	contributor
Bunduc, S	Center for Digestive Diseases and Liver Transplant, Fundeni Clinical Institute, 022328, Bucharest, Romania	contributor
Burgueño-Gómez, B	Digestive Diseases Department Clinical University Hospital of Valladolid, Valladolid, Spain	contributor
Burridge, IS	Liverpool University Hospitals NHS Foundation Trust, Liverpool, UK	contributor
Bush, N	Department of Gastroenterology, Postgraduate Institute of Medical Education and Research (PGIMER), Chandigarh, India	contributor
Bühler, F	Else Kröner-Fresenius-Zentrum für Ernährungsmedizin (EKFZ), Technische Universität München (TUM), Freising, Germany	contributor
Capunge, I	Clínica Sagrada Esperança, Luanda, Angola	contributor
Capurso, G	Pancreas Translational and Clinical Research Center, San Raffaele Scientific Institute, Vita Salute San Raffaele University, Milan, Italy	contributor
Carr, G	Epithelial Research Group, Institute for Cell and Molecular Biosciences, University of Newcastle, Newcastle, UK	contributor
Carvalho, JR	Department of Gastroenterology and Hepatology, North Lisbon Hospital Center, Hospital Santa Maria, University of Lisbon, Lisbon, Portugal	contributor
Casadei, R	Division of Pancreatic Surgery, IRCCS, Azienda Ospedaliero Universitaria di Bologna, Bologna, Italy	contributor
Castiñeira, AQ	Department of Gastroenterology, University Hospital of Santiago de Compostela, Santiago de Compostela, Spain	contributor
Cattani, MM	Department of Medicine, Gastroenterology, The Pancreas Institute, G.B. Rossi University Hospital, Verona, Italy	contributor
Cazacu, I	Research Center of Gastroenterology and Hepatology, Craiova, Romania	contributor
Cennamo, V	Unit of Gastroenterology and Digestive Endoscopy, Ausl Bologna Bellaria, Maggiore Hospital, Bologna, Italy	contributor

Name	Institute	Role
Ceranowicz, P	Department of Physiology, Faculty of Medicine, Jagiellonian University Medical College, Krakow, Poland	contributor
Chandak, G R	Centre for Cellular and Molecular Biology (CCMB), Council of Scientific and Industrial Research (CSIR), Hyderabad, India	contributor
Chang, JH	Bucheon St. Mary's Hospital, College of Medicine, The Catholic University of Korea, Seoul, Republic of Korea	contributor
Chang, MC	Division of Gastroenterology and Hepatology, Department of Internal Medicine, National Taiwan University Hospital, Taipei, Taiwan	contributor
Chang, YT	Division of Gastroenterology and Hepatology, Department of Internal Medicine, National Taiwan University Hospital, Taipei, Taiwan	contributor
Chen, J-M	Department for Internal Medicine, Neurology and Dermatology, Division of Gastroenterology, University of Leipzig, Leipzig, Germany	contributor
Chen, W	Department of Gastroenterology, Clinical Medical College, Yangzhou University, Yangzhou, Jiangsu, China	contributor
Choi, EK	Department of Internal Medicine, Jeju National University College of Medicine, Jeju, South Korea	contributor
Chooklin, S	Lviv Regional Clinical Hospital, Lviv, Ukraine	contributor
Choudhuri, G	Department of Gastroenterology, Sanjay Gandhi Postgraduate Institute of Medical Sciences, Lucknow, India	contributor
Chuklin, S	Lviv Regional Clinical Hospital, Lviv, Ukraine	contributor
Cirera, A	Department of Hepato-Pancreato-Biliary and Transplant Surgery, Hospital Universitari Vall d'Hebron, Universitat Autònoma de Barcelona, Barcelona, Spain	contributor
Ciubotaru, C	Emergency Hospital of Bucharest, Carol Davila University of Medicine and Pharmacy Bucharest, Bucharest, Romania	contributor
Comas, EP	University Hospital Arnau de Vilanova, Hospital University Santa Maria, Lleida, Spain	contributor
Constantinescu, G	Emergency Hospital of Bucharest, Carol Davila University of Medicine and Pharmacy Bucharest, Bucharest, Romania	contributor
Constantino, J	Unidade HBP, Serviço de Cirurgia Geral, Centro Hospitalar Tondela-Viseu, Viseu, Portugal	contributor
Crai, S	Pándy Kálmán Hospital of Békés County, Gyula, Hungary	contributor
Crul, T	Department of Medicine, University of Szeged, Szeged, Hungary	contributor
Cuadrado, AJ	Department of Gastroenterology, University Hospital of Santiago de Compostela, Santiago de Compostela, Spain	contributor
Czakó, B	Division of Gastroenterology, Department of Medicine, University of Szeged, Szeged, Hungary	contributor
Czakó, L	Department of Medicine, University of Szeged, Szeged, Hungary	contributor
Czapári, D	Institute for Translational Medicine, Medical School, University of Pécs, Pécs, Hungary	contributor
Czimmer, J	Department of Gastroenterology, First Department of Medicine, Medical School, University of Pécs, Pécs, Hungary	contributor
Czira, B	Department of Pathophysiology, University of Szeged, Szeged, Hungary	contributor
Czopf, L	Medical School, First Department of Medicine, Division of Cardiology and Angiology, University of Pécs, Pécs, Hungary	contributor
Csathó, Á	Medical School, Institute of Behavioural Sciences, University of Pécs, Pécs, Hungary	contributor
Csáti, S	First Department of Medicine, University of Szeged, Szeged, Hungary	contributor
Csefkó, K	Department of Gastroenterology, BVKK Dr. Réthy Pál Hospital of County Békés, Békéscsaba, Hungary	contributor
Csekő, K	Department of Pharmacology and Pharmacotherapy, Medical School, University of Pécs, Pécs, Hungary	contributor
Csernay-Biró, P	First Department of Internal Medicine, University of Szeged, Szeged, Hungary	contributor
Csiszkó A,	Institute of Surgery, University of Debrecen, Clinical Center, Debrecen, Hungary	contributor
Csont, T	Department of Biochemistry, University of Szeged, Szeged, Hungary	contributor
Csupor, D	Institute of Pharmacognosy, Faculty of Pharmacy, University of Szeged, Szeged, Hungary	contributor
Darvasi, E	First Department of Medicine, University of Szeged, Szeged, Hungary	contributor
Das, K	Division of Gastroenterology, School of Digestive and Liver Diseases, IPGME &R, Kolkata, India	contributor
De la Iglesia, D	Gastroenterology Department, University Hospital of Santiago de Compostela, Santiago de Compostela, Spain	contributor
Deák, PÁ	Division of Oncological Intervention, Department of Interventional Radiology, Heart and Vascular Centre, Semmelweis University, Budapest, Hungary	contributor
Del Chiaro, M	Division of Surgical Oncology, Department of Surgery - University of Colorado Anschutz Medical Campus, Denver, USA	contributor
Delaporte, C	Else Kröner-Fresenius-Zentrum für Ernährungsmedizin (EKFZ), Technische Universität München (TUM), Freising, Germany.	contributor
Deli, MA	Institute of Biophysics, Biological Research Centre, Szeged, Hungary	contributor
de-Madaria, E	Gastroenterology Department, Alicante University General Hospital, Alicante Institute for Health and Biomedical Research (ISABIAL), Alicante, Spain	contributor
Dembrovszky, F	Institute for Translational Medicine, Medical School, University of Pécs, Pécs, Hungary	contributor
Demcsák, A	Department of Surgery, University of California Los Angeles, Los Angeles, USA	contributor
Demeter, D	Dietetic Services, Central Hospital of Northern Pest - Military Hospital, Budapest, Hungary	contributor
Dénes, M	Second Department of Surgery, County Hospital Targu Mures, Targu Mures, Romania	contributor
Deng, L	Department of Integrated Traditional Chinese and Western Medicine, Sichuan Provincial Pancreatitis Center and West China-Liverpool Biomedical Research Center, West China Hospital, Sichuan University, C[illegible]	contributor

Name	Institute	Role
Derikx, MH	Department of Molecular and Cell Biology, Boston University Henry M. Goldman School of Dental Medicine, Boston, MA, USA	contributor
Diszházi, G	Department of Physiology, University of Debrecen, Debrecen, Hungary	contributor
Dobai, BM	George Emil Palade University of Medicine, Pharmacy, Science and Technology of Targu Mures, Targu, Mures, Romania	contributor
Dobszai, D	Institute for Translational Medicine, Medical School, University of Pécs, Pécs, Hungary	contributor
Dohos, D	Institute for Translational Medicine, Medical School, University of Pécs, Pécs, Hungary, Heim Pál National Institute of Pediatrics, Budapest, Hungary	contributor
Dolman, NJ	Molecular Probes Labelling and Detection Technologies, Life Technologies Corporation, Eugene, OR, USA	contributor
Dominguez-Munoz, JE	Department of Gastroenterology, University Hospital of Santiago de Compostela, Santiago de Compostela, Spain	contributor
Doros, A	Department of Interventional Radiology, Heart and Vascular Centre, Semmelweis University, Budapest, Hungary	contributor
Dósa, S	Department of Pathology, University of Szeged, Szeged, Hungary	contributor
Döbrönte, Z	Second Department of Medicine, Semmelweis University, Budapest, Hungary	contributor
Dronov, O	General Surgery #1, Bogomolets National Medical University, Kiev, Ukraine	contributor
Dubravcsik, Z	Bács-Kiskun County Hospital, Kecskemét, Hungary	contributor
Dubtsova, EA	A.S. Loginov Moscow Clinical Scientific Center, Moscow, Russia	contributor
Ducza, E	Department of Pharmacodynamics and Biopharmacy, Faculty of Pharmacy, University of Szeged, Szeged, Hungary	contributor
Duda, E	Biological Research Center, Institute of Biochemistry, Hungarian Academy of Sciences, Szeged, Hungary	contributor
Dudás, K	Department of Medicine, University of Szeged, Szeged, Hungary	contributor
Duerr, J	Department of Translational Pulmonology, Translational Lung Research Center Heidelberg, German Center for Lung Research (DZL), University of Heidelberg, Heidelberg, Germany	contributor
Duman, DG	Marmara University Education and Training Hospital, Istanbul, Turkey	contributor
Dumitru, A	Faculty of Medicine, Ovidius University of Constanta, Constanta, Romania	contributor
Dumitru, E	Faculty of Medicine, Ovidius University of Constanta, County, Emergency, and Clinical Hospital of Constanta, Constanta, Romania	contributor
Dunás-Varga, V	Szent György University Teaching Hospital of Fejér County, Székesfehérvár, Hungary	contributor
Durko, L	Department of Digestive Tract Diseases, Medical University of Lodz, Lodz, Poland	contributor
Ébert, A	Department of Pharmacology and Pharmacotherapy, University of Szeged, Szeged, Hungary	contributor
El Sayed, G	The Royal Bournemouth Hospital, University Hospital Dorset, Bournemouth, UK	contributor
Éliás, AJ	Center for Translational Medicine, Semmelweis University, Budapest, Hungary	contributor
Eminler, AT	Department of Gastroenterology, Faculty of Medicine, Sakarya University, Sakarya, Turkey	contributor
Enchev, E T	Department of Surgery, University Hospital, Stara Zagora, Bulgaria	contributor
Engelhardt, R	Department of Gastroenterology, Hepatology and Endocrinology, Hannover Medical School, Hannover, Germany	contributor
Eperjesi, O	Institute of Pancreatic Diseases, Semmelweis University, Budapest, Hungary	contributor
Erdős, R	First Department of Medicine, University of Szeged, Szeged, Hungary	contributor
Erdősi, D	Institute for Translational Medicine, Medical School, University of Pécs, Pécs, Hungary	contributor
Erős, A	Heim Pál Children's Hospital, Budapest, Hungary	contributor
Erőss, B	Institute of Pancreatic Diseases, Semmelweis University, Budapest, Hungary	contributor
Ewers, M	Pediatric Nutritional Medicine & Else Kröner-Fresenius-Centre for Nutritional Medicine (EKFZ), Technical University Munich (TUM), Freising, Germany	contributor
Fabisiak, A	Department of Digestive Tract Diseases, Medical University of Lodz, Lodz, Poland	contributor
Fabisiak, N	Department of Gastroenterology Medical University of Lodz, Poland	contributor
Faluhelyi, N.	Department of Medical Imaging, Medical School, University of Pécs, Pécs, Hungary	contributor
Fanczal, J	Department of Medicine, University of Szeged, Szeged, Hungary	contributor
Farkas, A	Second Department of Internal Medicine and Cardiological Center, University of Szeged, Szeged, Hungary	contributor
Farkas, G	Department of Surgery, University of Szeged, Szeged, Hungary	contributor
Farkas, G Jr	Department of Surgery, University of Szeged, Szeged, Hungary	contributor
Farkas, H	County Emergency Clinical Hospital of Târgu Mures-Gastroenterology Clinic and University of Medicine, Pharmacy, Sciences and Technology 'George Emil Palade', Targu Mures, Romania	contributor
Farkas, N	Institute of Bioanalysis, Medical School, University of Pécs, Pécs, Hungary	contributor
Farkas, N	First Department of Medicine, University of Szeged, Szeged, Hungary	contributor
Farkas, O	Department of Medical Imaging, Medical School, University of Pécs, Pécs, Hungary	contributor
Fazekas, A	Institute for Translational Medicine, Medical School, University of Pécs, Pécs, Hungary	contributor

Name	Institute	Role
Fehér, KE	Department of Internal Medicine, Division of Gastroenterology, Faculty of Medicine, University of Debrecen, Debrecen, Hungary	contributor
Fehérvári, P	Department of Biostatistics, University of Veterinary Medicine, Budapest, Hungary	contributor
Fejes, R	Szent György Teaching Hospital of County Fejér, Székesfehérvár, Hungary	contributor
Férec, C	Institut National de la Santé et de la Recherche Médicale (INSERM), Etablissement Français du Sang (EFS)–Bretagne, Brest, France	contributor
Fernandes, SR	Department of Gastroenterology and Hepatology, North Lisbon Hospital Center, Hospital Santa Maria, University of Lisbon, Lisbon, Portugal	contributor
Fernandez-Del Castillo, C	Department of Surgery, Massachusetts General Hospital, Harvard Medical School, Boston, MA, USA	contributor
FitzGerald, GA	Institute for Translational Medicine and Therapeutics, Perelman School of Medicine, University of Pennsylvania, Philadelphia, USA	contributor
Fogarasi, B	Institute of Pancreatic Diseases, Semmelweis University, Budapest, Hungary	contributor
Foldesi, I	Department of Laboratory Medicine, University of Szeged, Szeged, Hungary	contributor
Forsmark, CE	Division of Gastroenterology, Hepatology, and Nutrition, University of Florida, Gainesville, FL, USA	contributor
Földes, A	Department of Oral Biology, Semmelweis University, Budapest, Hungary	contributor
Földesi, I	Department of Laboratory Medicine, University of Szeged, Szeged, Hungary	contributor
Földi, I	Department of Gastroenterology, Institute of Internal Medicine, Faculty of Medicine, University of Debrecen, Debrecen, Hungary	contributor
Földi, M	Heim Pál National Pediatric Institute, Budapest, Hungary	contributor
Francisco, E	Surgery Department, Hospital Prof. Ferndo Fonseca, Amadora, Portugal	contributor
Franklin, J	The Royal Bournemouth Hospital, University Hospital Dorset, Institute of Medical Imaging and Visualisation, Bournemouth, UK	contributor
Freitas, M	Department of Gastroenterology, Peking Union Medical College Hospital, Chinese Academy of Medical Sciences & Peking Union Medical College, Beijing, China	contributor
Friess, H	Department of Surgery, TUM, Munich, Germany	contributor
Frim, L	Institute for Translational Medicine, Medical School, University of Pécs, Pécs, Hungary	contributor
Fülöp, F	Institute of Pharmaceutical Chemistry, University of Szeged, Szeged, Hungary	contributor
Fűr, G	Department of Pathophysiology, University of Szeged, Szeged, Hungary	contributor
Fürst, E	Institute of Pancreatic Diseases, Semmelweis University, Budapest, Hungary	contributor
Gaál, Sz	Department of Emergency Medicine, Semmelweis University, Budapest, Hungary	contributor
Gácser, A	Department of Microbiology, University of Szeged, Szeged, Hungary	contributor
Gagyi, EB	Center for Translational Medicine, Semmelweis University, Budapest, Hungary	contributor
Gajdán, L	Szent György Teaching Hospital of County Fejér, Székesfehérvár, Hungary	contributor
Gál, E	Department of Pharmacology and Pharmacotherapy, University of Szeged, Szeged, Hungary	contributor
Gál, J	Department of Anesthesiology and Intensive Therapy, Semmelweis University, Budapest, Hungary	contributor
Galbács, G	Department of Molecular and Analytical Chemistry, University of Szeged, Szeged, Hungary	contributor
Galeev, S	Saint Luke Clinical Hospital, St. Petersburg, Russia	contributor
Galego, M	Gastroenterology Department, University Hospital of Santiago de Compostela, Santiago de Compostela, Spain	contributor
Gambin, T	Department of Medical Genetics, Institute of Mother and Child, Warsaw, Poland; Institute of Computer Science, Warsaw University of Technology, Warsaw, Poland	contributor
Garami, A	Institute for Translational Medicine, Medical School, University of Pécs, Pécs, Hungary	contributor
Garcia, DI	Department of Gastroenterology, University Hospital of Santiago de Compostela, Santiago de Compostela, Spain	contributor
Garmaa, G	Centre for Translational Medicine, Semmelweis University, Budapest, Hungary	contributor
Gąsiorowska, A	Department of Gastroenterology Medical, University of Lodz, Lodz, Poland	contributor
Gede, N	Institute for Translational Medicine, Medical School, University of Pécs, Pécs, Hungary	contributor
Geisz, A	Department of Molecular and Cell Biology, Henry M. Goldman School of Dental Medicine, Boston University, Boston, USA	contributor
Gellért, B	Department of Surgery, Transplantation and Gastroenterology, Semmelweis University, Budapest, Hungary	contributor
Gergő, D	Department of Pharmacognosy, Semmelweis University, Budapest, Hungary	contributor
Gervain, J	Szent György University Teaching Hospital of Fejér County, Székesfehérvár, Hungary	contributor
Gheorghe, C	"Carol Davila" University of Medicine and Pharmacy, Bucharest, Romania, Clinical Institute Fundeni, Bucharest, Romania	contributor
Gherbon, A	Discipline of Internal Medicine: Diabetes, Nutrition, Metabolic Diseases and Systemic Rheumatology, Victor Babeş University of Medicine and Pharmacy Timisoara, Timisoara, Romania	contributor
Girán, J	Department of Public Health Medicine, Medical School, University of Pécs, Pécs, Hungary	contributor
Głuszek, S	Collegium Medicum, The Jan Kochanowski University in Kielce, Kielce, Poland	contributor
Gódi, S	Division of Gastroenterology, First Department of Medicine, Medical School, University of Pécs, Pécs, Hungary	contributor

Name	Institute	Role
Góg, Cs	Healthcare Center of County Csongrád, Makó, Hungary	contributor
Golovics, P	First Department of Medicine, Semmelweis University, Budapest, Hungary	contributor
Gombos, K	Department of Laboratory Medicine, Medical School, University of Pécs, Pécs, Hungary	contributor
Gomes, A	Surgery Department, Hospital Prof. Ferndo Fonseca, Amadora, Portugal	contributor
Gómez-Jurado, MJ	Department of Hepato-Pancreato-Biliary and Transplant Surgery, Hospital Universitari Vall d'Hebron, Universitat Autònoma de Barcelona, Barcelona, Spain	contributor
Gonçalves, TC	Gastroenterology Department, Hospital da Senhora da Oliveira, Guimarães, Portugal	contributor
Gong, L	"Carol Davila" University of Medicine and Pharmacy, Bucharest, Romania	contributor
Gordon, C	The Royal Bournemouth Hospital, University Hospital Dorset, Bournemouth, UK	contributor
Gougol, A	Division of Gastroenterology, Hepatology and Nutrition, Department of Medicine, University of Pittsburgh School of Medicine, Pittsburgh, PA, USA	contributor
Gökbulut, V	Department of Gastroenterology, Yüksek Ihtisas Hastanesi, Ankara, Turkey	contributor
Göltl, P	Department of Medicine II, University Medical Center Mannheim, Medical Faculty Mannheim, Heidelberg University, Mannheim, Germany	contributor
Görbe, A	Institute for Translational Medicine, Szentágothai Research Centre, Medical School, University of Pécs, Pécs, Hungary	contributor
Görög, M	Department of Medicine, University of Szeged, Szeged, Hungary	contributor
Grammatikopoulos, T	Paediatric Liver, GI & Nutrition Centre, King's College Hospital, London, United Kingdom	contributor
Grassalkovich, A	Department of Medicine, University of Szeged, Szeged, Hungary	contributor
Gray, M	Biosciences Institute, Newcastle University, Newcastle upon Tyne, UK	contributor
Gray, Z	National Institute for Health Research, Southampton, UK	contributor
Griffiths, CEM	Dermatology Centre, NIHR Manchester Biomedical Research Centre, University of Manchester, Manchester, UK	contributor
Groneberg, D A	Institute for Occupational Medicine, GoetheUniversität Frankfurt, Frankfurt, Germany	contributor
Grützmann, R	Department of Surgery, Universitätsklinikum Dresden, Dresden, Germany	contributor
Gukovskaya, A S	Veterans Affairs Greater Los Angeles Healthcare System and University of California, Los Angeles, USA	contributor
Gukovsky, Ilya	Research Center for Alcoholic Liver and Pancreatic Diseases and Department of Medicine, University of California, Los Angeles and Veterans Affairs Greater Los Angeles Healthcare System, USA	contributor
Gunay, S	İzmir Katip Çelebi University Atatürk Training and Research Hospital, Karabaglar/Izmir, Turkey	contributor
Gyökeres, T	Department of Gastroenterology, State Health Centre, Budapest, Hungary	contributor
Gyömbér, Zs	1st Department of Medicine, University of Szeged,Szeged, Hungary	contributor
Gyöngyi, Z	Department of Public Health Medicine, Medical School, University of Pécs, Pécs, Hungary	contributor
Habon, T	Division of Cardiology, First Department of Medicine, University of Pécs Medical School, Pécs, Hungary	contributor
Hadani, Y	Centre for Translational Medicine, Semmelweis University, 1085, Budapest, Hungary	contributor
Hágendorn, R	Department of Gastroenterology, First Department of Medicine, Medical School, University of Pécs, Pécs, Hungary	contributor
Hagymási, K	Department of Surgery, Transplantation and Gastroenterology, Semmelweis University, Budapest, Hungary	contributor
Halász, A	Szent György Teaching Hospital of County Fejér, Székesfehérvár, Hungary	contributor
Halloran, C	Institute of Systems, Molecular and Integrative Biology, University of Liverpool and Liverpool University Hospitals NHS Foundation Trust, Liverpool, England, UK	contributor
Hamar, P	Centre for Translational Medicine, Semmelweis University, Budapest, Hungary	contributor
Hamvas, J	Peterfy Hospital, Budapest, Hungary	contributor
Han, J	Division of Gastroenterology, Department of Internal Medicine, Daegu Catholic University Medical Center and School of Medicine, Daegu, South Korea	contributor
Hanák, L	Institute for Translational Medicine, Medical School, University of Pécs, Pécs, Hungary	contributor
Hankó, B	University Pharmacy, Department of Pharmacy Administration, Semmelweis University, Budapest, Hungary	contributor
Harangi, F	Department of Paediatrics, Balassa János Hospital of County Tolna, Szekszárd, Hungary	contributor
Harazin, A	Institute of Biophysics, Biological Research Centre, Szeged, Hungary	contributor
Hartung, I	Medical School, Institute of Behavioural Sciences, University of Pécs, Pécs, Hungary	contributor
Hasegawa, M	DNAVEC Corporation, Tsukuba, Ibaraki, Japan	contributor
Havelda, L	Institute of Pancreatic Diseases, Semmelweis University, Budapest, Hungary	contributor
He, W	Department of Gastroenterology, First Affiliated Hospital of Nanchang University, Nanchang, China	contributor
Hegyi, E	Institute for Translational Medicine, Medical School, University of Pécs, Pécs, Hungary	contributor
Hegyi, PJ	Center for Translational Medicine, Semmelweis University, Budapest, Hungary	contributor
Helyes, Z	Department of Pharmacology and Pharmacotherapy, Medical School, University of Pécs, Pécs, Hungary	contributor

Name	Institute	Role
Hirth, M	Department of Medicine II, University Medical Center Mannheim, Medical Faculty Mannheim, Heidelberg University, Mannheim, Germany	contributor
Hoferica, J	Clinic of Internal Medicine - Gastroenterology, Jessenius Faculty of Medicine in Martin, Comenius University, Bratislava, Slovakia	contributor
Hohwieler, M	Department of Internal Medicine I, Ulm University Hospital, Ulm, Germany	contributor
Holgate, S	Clinical and Experimental Sciences, Faculty of Medicine, University of Southampton, Southampton, UK	contributor
Hollenbach, M	Division of Gastroenterology, University of Leipzig Medical Center, Leipzig, Germany	contributor
Holzinger, G	First Department of Medicine, University of Szeged Faculty of Medicine, Szeged, Hungary	contributor
Horváth, G	Department of Medical Biochemistry, Semmelweis University, Budapest, Hungary	contributor
Horváth, IL	University Pharmacy, Department of Pharmacy Administration, Semmelweis University, Budapest, Hungary	contributor
Horváth, V	Department of Internal Medicine, Semmelweis University of Medicine, Budapest, Hungary	contributor
Hosszúfalusi, N	Department of Internal Medicine and Hematology, Semmelweis University, Budapest, Hungary	contributor
Hracskó, Zs	Department of Biochemistry and Molecular Biology, University of Szeged, Szeged, Hungary	contributor
Hritz, I	First Department of Medicine, University of Szeged, Szeged, Hungary	contributor
Huang, W	Department of Integrated Traditional Chinese and Western Medicine, Sichuan Provincial Pancreatitis Center and West China-Liverpool Biomedical Research Center, West China Hospital, Sichuan University, Cl	contributor
Hunor-Pál, F	County Emergency Clinical Hospital, University of Medicine, Pharmacy, Sciences and Technology of Targu Mures, Targu Mures, Romania	contributor
Husain, S	Department of Pediatrics, Children's Hospital of Pittsburgh of University of Pittsburgh Medical Center, Pittsburgh, PA, USA	contributor
Hussein, T	Division of Pancreatic Disorders, Heart and Vascular Centre, Semmelweis University, Budapest, Hungary	contributor
Ignatavicius, P	Kyiv City Clinical Emergency Hospital, Kiev, Ukraine	contributor
Ignáth, I	First Department of Medicine, University of Szeged, Szeged, Hungary	contributor
Illés, A	Division of Gastroenterology, First Department of Medicine, Medical School, University of Pécs, Pécs, Hungary	contributor
Illés, D	Division of Gastroenterology, Department of Medicine, University of Szeged, Szeged, Hungary	contributor
Imrei, M	Heim Pál National Pediatric Institute, Budapest, Hungary	contributor
Ince, AT	School of Medicine, Hospital of Bezmialem Vakif University, Istanbul, Turkey	contributor
Ingaldi, C	Division of Pancreatic Surgery, IRCCS, Azienda Ospedaliero Universitaria di Bologna, Bologna, Italy	contributor
Inoue, M	DNAVEC Corporation, Tsukuba, Ibaraki, Japan	contributor
Inui, K	Department of Gastroenterology, Second Teaching Hospital, Fujita Health University, Nagoya, Japan	contributor
Ioannidis, O	4th Department of Surgery, Medical School, Aristotle University of Thessaloniki, General Hospital "George Papanikolaou", Thessaloniki, Greece	contributor
Iordache, FM	mergency Hospital of Bucharest, Carol Davila University of Medicine and Pharmacy Bucharest, Bucharest, Romania	contributor
Ivány, E	First Department of Medicine, University of Szeged Faculty of Medicine, Szeged, Hungary	contributor
Iványi, B	Department of Pathology, University of Szeged, Szeged, Hungary	contributor
Izbéki, F	Szent György Teaching Hospital of County Fejér, Székesfehérvár, Hungary	contributor
Janecke, A	Universitätsklinik für Pädiatrie I, Department für Kinder-und Jugendheilkunde, Medizinische Universität Innsbruck, Innsbruck, Austria	contributor
Janka, T	Division of Gastroenterology, Department of Internal Medicine, Faculty of Medicine, University of Debrecen, Debrecen, Hungary	contributor
Janovszky, Á	Institute of Surgical Research, University of Szeged, Szeged, Hungary	contributor
Jármay, K	First Department of Medicine, University of Szeged, Szeged, Hungary	contributor
Jassem, J	Medical University of Gdańsk, Gdańsk, Poland	contributor
Jászai, VA	Semmelweis University, Budapest, Hungary	contributor
Jinga, M	Department of Surgery, Lithuanian University of Health Sciences, Kaunas, Lithuania	contributor
Joly, F	Centre for Intestinal Failure, Department of Gastroenterology and Nutritional Support, Hôpital Beaujon, Clichy, France	contributor
Jovine, E	Department of Surgery, AOU Sant'Orsola Malpighi, IRCCS Azienda Ospedaliera Universitaria, Bologna, Italy	contributor
Judák, L	First Department of Medicine, University of Szeged, Szeged, Hungary	contributor
Juhász, MF	Heim Pál National Pediatric Institute, Budapest, Hungary	contributor
Jumaa, H	Eskilstuna Hospital, Mälarsjukhuset, Eskilstuna, Sweden	contributor
Jung, MK	Division of Gastroenterology and Hepatology, Department of Internal Medicine, Korea University Anam Hospital, Seoul, Republic of Korea	contributor
Kacar, S	Department of Gastroenterology, Yüksek Ihtisas Hastanesi, Ankara, Turkey	contributor
Kahán, Zs	Department of Oncotherapy, University of Szeged, Szeged, Hungary	contributor
Kajner, Gy	Department of Molecular and Analytical Chemistry, University of Szeged, Szeged, Hungary	contributor

Name	Institute	Role
Kakuta, E	Division of Gastroenterology, Tohoku University Graduate School of Medicine, Sendai, Miyagi, Japan	contributor
Kamlage, B	Metanomics Health GmbH, Berlin, Germany	contributor
Kani, HT	Department of Gastroenterology, Marmara University, School of Medicine, Istanbul, Turkey	contributor
Kanizsai, P	Department of Emergency Medicine, Medical School, University of Pécs, Pécs, Hungary	contributor
Karamya, ZA	Division of Gastroenterology, Department of Medicine, University of Szeged, Szeged, Hungary	contributor
Kárász, K	Kanizsai Dorottya Hospital, Nagykanizsa, Hungary	contributor
Karg, E	Department of Pediatrics, University of Szeged, Szeged, Hungary	contributor
Károly, S	George Emil Palade University of Medicine, Pharmacy, Science and Technology of Targu Mures, Targu Mures, Romania	contributor
Kaszaki, J	9Institute of Surgical Research, University of Szeged, Szeged, Hungary	contributor
Kató, D	Institute for Translational Medicine, Szentágothai Research Centre, Medical School, University of Pécs, Pécs, Hungary	contributor
Katona, X	First Department of Medicine, University of Szeged, Szeged, Hungary	contributor
Kchaou, A	Habib Bourguiba University Hospital, Sfax, Tunisia	contributor
Keim, V	Department for Internal Medicine, Neurology and Dermatology, Division of Gastroenterology, University of Leipzig, Leipzig, Germany	contributor
Kelemen, D	Surgery Clinic, Pecsi Tudomanyegyetem, Pecs, Hungary	contributor
Kemény, L	Department of Dermatology and Allergology, University of Szeged, Szeged, Hungary	contributor
Kemény, L	First Department of Medicine, University of Szeged, Szeged, Hungary	contributor
Kéri, A	Department of Molecular and Analytical Chemistry, University of Szeged, Szeged, Hungary	contributor
Khatkov, IE	A.S. Loginov Moscow Clinical Scientific Center, Moscow, Russia	contributor
Kim, EJ	Division of Gastroenterology, Department of Internal Medicine, Gachon University Gil Medical Center, Gachon University College of Medicine, Incheon, Republic of Korea	contributor
Kincses, L	Department of Pediatrics and Pediatric Health Centre, University of Szeged, Szeged, Hungary	contributor
Kiryukova, MA	A.S. Loginov Moscow Clinical Scientific Center, Moscow, Russia	contributor
Kiss, A	Department of Medicine, University of Szeged, Szeged, Hungary	contributor
Kiss, L	Department of Pathophysiology, University of Szeged, Szeged, Hungary	contributor
Kiss, S	Heim Pál National Pediatric Institute, Budapest, Hungary	contributor
Klauss, S	LMU University Hospital, LMU Munich, Munich, Germany	contributor
Kleger, A	Department of Internal Medicine I, Ulm University Hospital, Ulm, Germany	contributor
Kleiner, D	University Pharmacy, Department of Pharmacy Administration, Semmelweis University, Budapest, Hungary	contributor
Kocsis, I	Department of Laboratory Medicine, Semmelweis University, Budapest, Hungary	contributor
Kói, T	Department of Stochastics, Institute of Mathematics, Budapest University of Technology and Economics, Budapest, Hungary	contributor
Koksal, AS	Department of Gastroenterology, Faculty of Medicine, Sakarya University, Sakarya, Turkey	contributor
Koncz, B	First Department of Medicine, University of Szeged, Szeged, Hungary	contributor
Kónya, K	University of Medicine and Pharmacy of Târgu Mures, Târgu Mures, Romania	contributor
Kormányos, ES	Department of Pathophysiology, University of Szeged, Szeged, Hungary	contributor
Kormos, Z	Institute of Pancreatic Diseases, Semmelweis University, Budapest, Hungary	contributor
Korompay, A	Department of Pathology, Semmelweis University, Budapest, Hungary	contributor
Kosár, K	First Department of Medicine, University of Szeged Faculty of Medicine, Szeged, Hungary	contributor
Kovacheva-Slavova, MD	Department of Gastroenterology, Queen Yoanna University Hospital, Medical University of Sofia, Sofia, Bulgaria	contributor
Kovács, A	Department of Gastroenterology and Internal Medicine, Markusovszky Teaching Hospital, Szombathely, Hungary	contributor
Kovács, B	Division of Pancreatic Disorders, Heart and Vascular Centre, Semmelweis University, Budapest, Hungary	contributor
Kovács, DP	Department of Pathophysiology, University of Szeged, Szeged, Hungary	contributor
Kovács, G	Department of Gastroenterology, Institute of Internal Medicine, Faculty of Medicine, University of Debrecen, Debrecen, Hungary	contributor
Kovacs, P	Department for Internal Medicine, Neurology and Dermatology, Division of Endocrinology, University of Leipzig, Leipzig, Germany	contributor
Kovalska, I	General Surgery #1, Bogomolets National Medical University, Kiev, Ukraine	contributor
Kozachenko, A	Kharkiv Emergency Hospital, Medical Faculty of V. N. Karazin Kharkiv National University, Kharkiv, Ukraine	contributor
Koziel, D	Collegium Medicum, Jan Kochanowski University of Kielce, Kielce, Poland	contributor
Krüger, R	Department of Pediatrics, Division of Pediatric Pulmonology, Charité, Campus Virchow-Klinikum, Berlin, Germany	contributor

Name	Institute	Role
Kucserik, LP	Division of Surgery, Universitatea de Medicina si Farmacie din Targu Mures, Targu Mures, Romania	contributor
Kui, B	Department of Medicine, University of Szeged, Szeged, Hungary	contributor
Kukor, Z	Department of Medical Chemistry, Molecular Biology, and Pathobiochemistry, Semmelweis University, Budapest, Hungary	contributor
Kume, K	Division of Gastroenterology, Tohoku University Graduate School of Medicine, Sendai, Miyagi, Japan	contributor
Kurti, F	Department of Gastroenterology and Hepatology, University Hospital Center "Mother Theresa", Tirana, Albania	contributor
Kusnierz, K	Vilnius University Hospital Santariskiu Klinikos, Vilnius, Lithuania	contributor
Kuśnierz-Cabala, B	Department of Physiology, Faculty of Medicine, Jagiellonian University Medical College, Krakow, Poland	contributor
Kúthy-Sutus, E	MTA-SZTE Momentum Epithelial Cell Signaling and Secretion Research Group, University of Szeged, Szeged, Hungary	contributor
Kühn, JP	Institute of Radiology, Universitätsmedizin Greifswald, Greifswald, Germany	contributor
Laczkó, D	First Department of Medicine, University of Szeged, Szeged, Hungary	contributor
Lakatos, G	Dept. Oncology, St. Istvan and St. Laszlo Hospital and Out-Patient Department, Budapest, Hungary	contributor
Lakner, L	Second Department of Medicine, Semmelweis University, Budapest, Hungary	contributor
Lamont, T	NIHR Dissemination Centre, University of Southampton, Southampton, UK	contributor
Landt, O	TIB MOLBIOL, Berlin, Germany	contributor
Lankó, E	Department of Pharmaceutics and Central Clinical Pharmacy, University of Pécs, Pécs, Hungary	contributor
Lantos, T	Department of Medical Physics and Informatics, Faculty of Medicine, University of Szeged, Szeged, Hungary	contributor
Lasher, D	Else Kröner-Fresenius-Zentrum für Ernährungsmedizin (EKFZ), Technische Universität München (TUM), Freising, Germany	contributor
Lásztity, N	Department of Pediatrics, Szent János's Hospital and North Buda Unified Hospitals, Budapest, Hungary	contributor
Latawiec, D	Institute of Systems, Molecular and Integrative Biology, University of Liverpool and Liverpool University Hospitals NHS Foundation Trust, Liverpool, England, UK	contributor
Laumen, H	Pediatric Nutritional Medicine & Else Kröner-Fresenius-Centre for Nutritional Medicine (EKFZ), Technical University Munich (TUM), Freising, Germany; Department of Internal Medicine I, Martin-Luther-Univ	contributor
Laurence, J	Weill Cornell Medical College, New York, NY, USA	contributor
Lázár, B	Division of Pancreatic Disorders, Heart and Vascular Centre, Semmelweis University, Budapest, Hungary	contributor
Lázár, Gy	Department of Surgery, University of Szeged, Szeged, Hungary	contributor
Lazarescu, AM	County Emergency Clinical Hospital of Timisoara, Clinic II Pediatrics, Timisoara, Romania	contributor
Lee, HS	Division of Gastroenterology and Hepatology, Department of Internal Medicine, Korea University Anam Hospital, Seoul, Republic of Korea	contributor
Lee, J	Centre for Translational Medicine, Semmelweis University, Budapest, Hungary	contributor
Lee, S	Department of Internal Medicine and Liver Research Institute, Seoul National University Hospital, Seoul, Republic of Korea	contributor
Leindler, L	Department of Surgery, University of Szeged, Szeged, Hungary	contributor
Lerch, MM	Department of Medicine A, Universitatsmedizin Greifswald, Greifswald, Germany	contributor
Lesko, D	1st Department of Surgery, University Hospital of L. Pasteur, Kosice, Slovakia	contributor
Li, W	Surgical Intensive Care Unit (SICU), Department of General Surgery, Jinling Hospital, Medical School of Nanjing University, Nanjing, China	contributor
Li, Y	"Carol Davila" University of Medicine and Pharmacy, Bucharest, Romania	contributor
Lillik, V	Institute for Translational Medicine, Medical School, University of Pécs, Pécs, Hungary	contributor
Lindgren, F	Department of Pediatric, Karolinska University Hospital, Stockholm, Sweden	contributor
Lipp, M	Institute of Pancreatic Diseases, Semmelweis University, Budapest, Hungary	contributor
Litvin, A	Gomel State Medical University, Gomel, Belarus	contributor
Liu, Y	Department of Gastroenterology, Hepatology and Endocrinology, Hannover Medical School, Hannover, Germany	contributor
Lonovics, J	First Department of Medicine, University of Szeged, Szeged, Hungary	contributor
Lopez-Diaz, J	Department of Gastroenterology, University Hospital of Santiago de Compostela, Santiago de Compostela, Spain	contributor
Lowe, ME	Department of Pediatrics, Washington University School of Medicine, St Louis, MO, USA	contributor
Löhr, JM	Department of Clinical Science, Intervention and Technology, Karolinska Institutet, Stockholm, Sweden	contributor
Ludwig, M	Else Kröner-Fresenius-Zentrum für Ernährungsmedizin (EKFZ), Technische Universität München (TUM), Freising, Germany	contributor
Lukacs, GL	Department of Physiology, McGill University, Montreal, Canada	contributor
Macarie, M	County Emergency Clinical Hospital - Gastroenterology and, University of Medicine, Pharmacy, Sciences and Technology, Târgu Mureș, Romania	contributor
Macedo, V	Department of Gastroenterology, Peking Union Medical College Hospital, Chinese Academy of Medical Sciences & Peking Union Medical College, Beijing, China	contributor
Macek Jr., M	Department of Biology and Medical Genetics, University Hospital Motol and 2nd Faculty of Medicine of Charles University Prague, Prague, Czech Republic	contributor

Name	Institute	Role
Madácsy, L	2nd Department of Internal Medicine, Semmelweis University, Budapest, Hungary	contributor
Madácsy, T	Department of Medicine, University of Szeged, Szeged, Hungary	contributor
Maldonado, ER	General Surgery, Consorci Sanitari del Garraf, Sant Pere de Ribes, Barcelona, Spain	contributor
Małecka-Panas, E	Department of Digestive Tract Diseases, Medical University of Lodz, Lodz, Poland	contributor
Maléth, J	Department of Medicine, University of Szeged, Szeged, Hungary	contributor
Mall, MA	Department of Translational Pulmonology, Translational Lung Research Center Heidelberg, German Center for Lung Research (DZL), University of Heidelberg, Heidelberg, Germany	contributor
Mándi, Y	Department of Microbiology, University of Szeged, Szeged, Hungary	contributor
Marino, MV	General Surgery Department, Azienda Ospedaliera Ospedali Riuniti Villa Sofia-Cervello, Palermo, Italy	contributor
Marjai, T	First Department of Surgery, Semmelweis University, Budapest, Hungary	contributor
Márta, K	Institute of Pancreatic Diseases, Semmelweis University, Budapest, Hungary	contributor
Márton, Z	First Department of Medicine, Medical School, University of Pécs, Pécs, Hungary	contributor
Masamune, A	Division of Gastroenterology, Tohoku University Graduate School of Medicine, Sendai, Japan	contributor
Masson, E	Institut National de la Santé et de la Recherche Médicale (INSERM), U1078, Etablissement Français du Sang (EFS)–Bretagne, Brest, France	contributor
Mastrangelo, L	Department of Surgery, AOU Sant'Orsola Malpighi, IRCCS Azienda Ospedaliera Universitaria, Bologna, Italy	contributor
Mateescu, RB	"Carol Davila" University of Medicine and Pharmacy, Bucharest, Romania	contributor
Mátrai, P	Institute for Translational Medicine, Medical School, University of Pécs, Pécs, Hungary	contributor
Maurovich-Horváth, P	Department of Radiology, Medical Imaging Centre, Semmelweis University, Budapest, Hungary	contributor
Mayerle, J	Medizinische Klinik und Poliklinik II, Klinikum der Universität München, München, Germany	contributor
McCrudden, R	The Royal Bournemouth Hospital, University Hospital Dorset, Bournemouth, UK	contributor
Meczker, Á	Institute for Translational Medicine, Medical School, University of Pécs, Pécs, Hungary	contributor
Merkely, B	Heart and Vascular Center, Semmelweis University, Budapest, Hungary	contributor
Mickevicius, A	Vilnius University Hospital Santariskiu Klinikos, Vilnius, Lithuania	contributor
Miklós, E	Institute for Translational Medicine, Medical School, University of Pécs, Pécs, Hungary	contributor
Miklós, V	University Biobank, University of Szeged, Szeged, Hungary	contributor
Mikó, A	Institute for Translational Medicine, Medical School, University of Pécs, Pécs, Hungary	contributor
Minkov, G A	Department of Surgery, University Hospital, Stara Zagora, Bulgaria	contributor
Mirabella, A	General and Emergency Surgery Department, Azienda Ospedaliera Ospedali Riuniti Villa Sofia-Cervello, Palermo, Italy	contributor
Miseta, A	Department of Laboratory Medicine, Medical School, University of Pécs, Pécs, Hungary	contributor
Misirlioglu Sucan, S	İzmir Katip Çelebi University Atatürk Training and Research Hospital, Karabaglar/Izmir, Turkey	contributor
Molcan, P	Hepatology and Gastroenterology Department of Roosevelt Hospital, Banska Bystrica, Slovakia	contributor
Molero, X	Exocrine Pancreas Research Unit, Hospital Universitari Vall d'Hebron, Institut de Recerca, Universitat Autònoma de Barcelona, CIBEREHD, Barcelona, Spain	contributor
Molnár, B	First Department of Medicine, University of Szeged, Szeged, Hungary	contributor
Molnár, M	Department of Medicine, University of Szeged, Szeged, Hungary	contributor
Molnár, R	Department of Medicine, University of Szeged, Szeged, Hungary	contributor
Molnár, T	MTA-SZTE Momentum Epithelial Cell Signaling and Secretion Research Group, University of Szeged, Szeged, Hungary	contributor
Molnár, Z	Department of Anestesiology and Intensive Care, University of Szeged, Szeged, Hungary	contributor
Molontay, R	Human and Social Data Science Lab, Budapest University of Technology and Economics, Budapest, Hungary	contributor
Monclús, NT	University Hospital Arnau de Vilanova, Hospital University Santa Maria, Lleida, Spain	contributor
Monteiro, J	Nature Medicine, New York, NY, USA	contributor
Monterisi, S	Department of Physiology, Anatomy and Genetics, Oxford University, Oxford, UK	contributor
Moore, D	Liverpool Pancreatitis Research Group, University of Liverpool and the Royal Liverpool and Broadgreen University Hospital Trust, Liverpool, UK	contributor
Morvay, M	Department of Radiology, University of Szeged, Szeged, Hungary	contributor
Morvay, Z	Department of Radiology, University of Szeged, Szeged, Hungary	contributor
Mosdósi, B	Department of Paediatrics, Medical School, University of Pécs, Pécs, Hungary	contributor
Mosztbacher, D	Institute for Translational Medicine, Medical School, University of Pécs, Pécs, Hungary, First Department of Paediatrics, Faculty of Medicine, Semmelweis University, Budapest, Hungary	contributor
Mössner, J	Department for Internal Medicine, Neurology and Dermatology, Division of Gastroenterology, University of Leipzig, Leipzig, Germany	contributor

Name	Institute	Role
Muallem, S	National Institute of Dental and Craniofacial Research, Bethesda, MD, USA	contributor
Mukherjee, R	University of Liverpool, Liverpool University Hospitals NHS Foundation Trust, Liverpool, UK	contributor
Müller, T	Universitätsklinik für Pädiatrie I, Department für Kinder-und Jugendheilkunde, Medizinische Universität Innsbruck, Innsbruck, Austria	contributor
Mykhailo, VM	Department of Surgery With a Course of Emergency and Vascular Surgery, Bogomolet National Medical University, Kiev, Ukraine	contributor
Nagy, A	Heim Pál National Pediatric Institute, Budapest, Hungary	contributor
Nagy, Á	Department of Dentistry, Oral and Maxillofacial Surgery, Medical School, University of Pécs, Pécs, Hungary	contributor
Nagy, F	Faculty of Medicine, First Department of Medicine, University of Szeged, Korányi fasor 8-10, H-6720, Szeged, Hungary	contributor
Nagy, G	Second Department of Internal Medicine, University of Debrecen, Debrecen, Hungary	contributor
Nagy, M	Human and Social Data Science Lab, Budapest University of Technology and Economics, Budapest, Hungary	contributor
Nagy, R	Heim Pál National Pediatric Institute, Budapest, Hungary	contributor
Nagy, T	Department of Laboratory Medicine, Medical School, University of Pécs, Pécs, Hungary	contributor
Nakano, E	Division of Gastroenterology, Tohoku University Graduate School of Medicine, Sendai, Miyagi, Japan	contributor
Nakov, R	Department of Gastroenterology, Queen Yoanna University Hospital, Medical University of Sofia, Sofia, Bulgaria	contributor
Nawacki, Ł	Collegium Medicum, The Jan Kochanowski University in Kielce, Kielce, Poland	contributor
Negoi, I	mergency Hospital of Bucharest, Carol Davila University of Medicine and Pharmacy Bucharest, Bucharest, Romania	contributor
Negoita, V	Surgery Department, Emergency Hospital of Bucharest, Carol Davila University of Medicine and Pharmacy, Bucharest, Romania	contributor
Nemes, O	Division of Endocrinology and Metabolism, First Department of Medicine, University of Pécs, Pécs, Hungary	contributor
Németh, A	Division of Pancreatic Disorders, Heart and Vascular Centre, Semmelweis University, Budapest, Hungary	contributor
Németh, BC	Department of Medicine, Albert Szent-Györgyi Medical School, University of Szeged, Szeged, Hungary	contributor
Németh, D	Institute for Translational Medicine, Szentágothai Research Centre, Medical School, University of Pécs, Pécs, Hungary	contributor
Németh, I	Data-Management, Pre-Clinical and Clinical Biostatistics, Adware Research Developing and Consulting Ltd, Balatonfüred, Hungary	contributor
Németh, J	First Department of Medicine, University of Szeged, Szeged, Hungary	contributor
Németh, M	First Department of Medicine, University of Szeged, Szeged, Hungary	contributor
Neoptolemos, J	Department of General Surgery, University of Heidelberg, Heidelberg, Germany	contributor
Nistal, RB	Servicio de Aparato Digestivo Hospital Clínico Universitario Valladolid, Valladolid, Spain	contributor
Nita, AF	Department of Paediatrics, Grigore Alexandrescu Emergency Hospital for Children, Bucharest, Romania	contributor
Nosáková, L	Clinic of Internal Medicine - Gastroenterology, JFM CU, Jessenius Faculty of Medicine in Martin (JFM CU), Comenius University in Bratislava, Bratislava, Slovakia	contributor
Novák, J	Pándy Kálmán Hospital of Békés County, Gyula, Hungary	contributor
Nunes, V	HPB Surgery, Department of Surgery, Hospital Prof. Dr. Fernando Fonseca, Amadora, Portugal	contributor
Nyári, G	Department of Pathology, University of Szeged, Szeged, Hungary	contributor
Oballe, JAR	University Hospital Arnau de Vilanova, Hospital University Santa Maria, Lleida, Spain	contributor
Ocskay, K	Institute for Translational Medicine, Medical School, University of Pécs, Pécs, Hungary	contributor
Oláh, A	Department of Surgery, Petz Aladár County Teaching Hospital, Győr, Hungary	contributor
Olah, E	Institute for Translational Medicine, Medical School, University of Pecs, Pecs, Hungary	contributor
Oláh, I	Ilona Tóth Outpatient Clinic, Budapest, Hungary	contributor
Oláh-Németh, O	Department of Pathology, University of Szeged, Szeged, Hungary	contributor
Olekcandr, AT	Kyiv City Clinical Emergency Hospital, Kiev, Ukraine	contributor
Oliveira, MJ	Department of Surgery, Hospital Prof. Dr. Fernando Fonseca, Amadora, Portugal	contributor
Opalchuk, K	Department of Surgery, Bogomolets National Medical University, Kyiv, Ukraine	contributor
Oracz, G	Department of Gastroenterology, Hepatology, Feeding Disorders and Pediatrics, The Children's Memorial Health Institute, Warsaw, Poland	contributor
Orján, EM	Department of Pathophysiology, University of Szeged, Szeged, Hungary	contributor
Ottóffy, M	Institute for Translational Medicine, University of Pécs Medical School, Pécs, Hungary	contributor
Ózsvári, B	First Department of Medicine, University of Szeged, Szeged, Hungary	contributor
Ördög, A	Department of Plant Biology, University of Szeged, Szeged, Hungary	contributor
Ördög, B	Department of Pharmacology and Pharmacotherapy, Faculty of Medicine, University of Szeged, Szeged, Hungary	contributor
Palatka, K	Division of Gastroenterology, Department of Internal Medicine, Faculty of Medicine, Debreceni Egyetem, Debrecen, Hungary	contributor

Name	Institute	Role
Pálinkás, D	Department of Gastroenterology, Military Hospital Medical Centre, Hungarian Defense Forces, Budapest, Hungary	contributor
Pallagi, P	Department of Medicine, University of Szeged, Szeged, Hungary	contributor
Pallagi-Kunstár, É	Faculty of Medicine, First Department of Medicine, University of Szeged, Korányi fasor 8-10, H-6720, Szeged, Hungary	contributor
Pálvölgyi, A	First Department of Medicine, University of Szeged, Szeged, Hungary	contributor
Pando, E	Department of Hepato-Pancreato-Biliary and Transplant Surgery, Hospital Universitari Vall d'Hebron, Universitat Autònoma de Barcelona, Barcelona, Spain	contributor
Pandol, SJ	Cedars-Sinai Medical Center and University of California, Los Angeles, USA	contributor
Pap, Á	Péterfy Sándor Hospital, Budapest, Hungary	contributor
Papachristou, G	Division of Gastroenterology, Hepatology and Nutrition, Department of Medicine, University of Pittsburgh School of Medicine, Pittsburgh, PA, USA	contributor
Papp, C	Department of Microbiology, University of Szeged, Szeged, Hungary	contributor
Papp, M	Medicine, University of Debrecen, Debrecen, Hungary	contributor
Papp, N	Department of Medicine, University of Szeged, Szeged, Hungary	contributor
Papp, R	Surgery Clinic, University of Pécs, Pécs, Hungary	contributor
Papp, Z	Institute for Translational Medicine, University of Pécs, Pécs, Hungary	contributor
Papuc, G	Gastroenterology Department, Colentina Clinical Hospital Bucharest, Bucharest, Romania	contributor
Pár, G	Division of Gastroenterology, First Department of Medicine, Medical School, University of Pécs, Pécs, Hungary	contributor
Párniczky, A	Heim Pál National Pediatric Institute, Budapest, Hungary	contributor
Patai, Á	Second Department of Medicine, Semmelweis Uiversity, Budapest, Hungary	contributor
Patai, Á	Markusovszky University Teaching Hospital, Szombathely, Hungary	contributor
Patoni, C	Carol Davila University of Medicine and Pharmacy, Bucharest, Romania	contributor
Pázmány, P	Heim Pál National Pediatric Institute, Budapest, Hungary	contributor
Pecze, L	Institute for Translational Medicine, Szentágothai Research Centre, Medical School, University of Pécs, Pécs, Hungary	contributor
Pécsi, D	Division of Gastroenterology, First Department of Medicine, Medical School, University of Pécs, Pécs, Hungary	contributor
Pelaez-Luna, M	Department of Gastroenterology, Pancreatic Disorders Unit, National Institute of Medical Sciences and Nutrition Salvador Zubiran, UNAM. Mexico City, Mexico	contributor
Pencik, P	Centrum péče o zažívací trakt, Vítkovická Nemocnice a.s., Ostrava, Czech Republic	contributor
Peng, S	Department of Pathophysiology, School of Medicine, Jinan University, Guangzhou, China	contributor
Perdomo, D	Department of Physiology, McGill University, Montreal, Quebec, Canada	contributor
Pereira, J	Unidade HBP, Serviço de Cirurgia Geral, Centro Hospitalar Tondela-Viseu, Viseu, Portugal	contributor
Perides, G	Department of Surgery, Tufts Medical Center, Boston, MA, US	contributor
Pesei, ZG	Center for Exocrine Disorders, Department of Molecular and Cell Biology, Boston University Henry M. Goldman School of Dental Medicine, Boston, MA, US	contributor
Petersen, OH	Cardiff University, Cardiff, UK	contributor
Pető, Z	Department of Emergency Medicine, University of Szeged, Szeged, Hungary	contributor
Pfützer, R	Department of Internal Medicine, Klinikum Döbeln, Döbeln, Germany	contributor
Philip, BM	Centre for Translational Medicine, Semmelweis University, Budapest, Hungary	contributor
Pickles, A	Biostatistics and Health Informatics, King's College London, London, UK	contributor
Pilsak, C	Else Kröner-Fresenius-Zentrum für Ernährungsmedizin (EKFZ), Technische Universität München (TUM), Freising, Germany	contributor
Pintér, J	Human and Social Data Science Lab, Budapest University of Technology and Economics, Budapest, Hungary	contributor
Pinto, B	Surgery Department, Hospital Prof. Ferndo Fonseca, Amadora, Portugal	contributor
Płoski, R	Department of Medical Genetics, Medical University of Warsaw, Warsaw, Poland	contributor
Poluektov, VL	Department of Surgery and Urology, Omsk State Medical University, Omsk, Russia	contributor
Poropat, G	Department of Gastroenterology, Clinical Hospital Center Rijeka, University of Rijeka, Rijeka, Croatia	contributor
Pósa, A	Department of Comparative Physiology, University of Szeged, Szeged, Hungary	contributor
Poto, L	Institute of Bioanalysis, Medical School, University of Pecs, Pecs, Hungary	contributor
Prónai, L	Second Department of Medicine, Semmelweis University, Budapest, Hungary	contributor
Puskás, LG	Laboratory of Functional Genomics, Biological Research Centre, Hungarian Academy of Sciences, Szeged, Hungary	contributor
Rabotyagova, Y	Medical Academy Named after S.I. Georgievsky, Crimean Federal University Named after V.I. Vernadsky, Simferopol, Russia	contributor
Radics, B	Department of Pathology, University of Szeged, Szeged, Hungary	contributor

Name	Institute	Role
Radovan, A	Department of Gastroenterology, Clinical Hospital Center Rijeka, University of Rijeka, Rijeka, Croatia	contributor
Rakk, D	Department of Microbiology, University of Szeged, Szeged, Hungary	contributor
Rakonczay, Z Jr.	Department of Pathophysiology, University of Szeged,Szeged, Hungary	contributor
Rama-Fernández, A	Gastroenterology Department, University Hospital of Santiago de Compostela, Santiago de Compostela, Spain	contributor
Ramírez-Maldonado, E	General Surgery, Consorci Sanitari del Garraf, Sant Pere de Ribes, Barcelona, Spain	contributor
Rana, SS	Department of Gastroenterology, Postgraduate Institute of Medical Education and Research (PGIMER), Chandigarh, India	contributor
Rasmussen, IC	Department of Surgery, Uppsala University Hospital, Uppsala, Sweden	contributor
Rázga, Z	Department of Pathology, University of Szeged, Szeged, Hungary	contributor
Rebollo, MLR	Servicio de Aparato Digestivo Hospital Clínico Universitario Valladolid, Valladolid, Spain	contributor
Rebours, V	Pancreatology Unit, Beaujon Hospital, APHP, Paris, Université de Paris, Paris-Diderot, France	contributor
Reddy, DN	Asian Institute of Gastroenterology, Hyderabad, India	contributor
Reiber, I	Division of Gastroenterology, Fejér County Saint George Teaching Hospital of the University of Pécs, Székesfehérvár, Hungary	contributor
Ricci, C	Division of Pancreatic Surgery, IRCCS, Azienda Ospedaliero Universitaria di Bologna, Bologna, Italy	contributor
Riederer, B	Department of Gastroenterology, Hepatology and Endocrinology, Hannover Medical School, Hannover, Germany	contributor
Riitta, S	Biotechnology and Molecular Medicine, A.I. Virtanen Institute for Molecular Sciences, Biocenter Kuopio, University of Eastern Finland, Kuopio, Finland	contributor
Rios, AS	Department of Gastroenterology, Pancreatic Disorders Unit, National Institute of Medical Sciences and Nutrition Salvador Zubiran, UNAM. Mexico City, Mexico	contributor
Ritter, E	Department of Pharmacology and Pharmacotherapy, Medical School, University of Pécs, Pécs, Hungary	contributor
Rodriguez-Oballe, JA	Department of Gastroenterology, University Hospital Santa María - University Hospital Arnau de Vilanova, Lerida, Spain	contributor
Rosendahl, J	Department of Internal Medicine I, Martin Luther University, Halle (Saale), Germany	contributor
Rostás, I	Institute for Translational Medicine, Szentágothai Research Centre, Medical School, University of Pécs, Pécs, Hungary	contributor
Rosztóczy, A	First Department of Medicine, University of Szeged, Szeged, Hungary	contributor
Rubovszky, G	National Institute of Oncology, Budapest, Hungary	contributor
Ruffert, C	Department of Internal Medicine, Neurology and Dermatology, Division of Gastroenterology and Rheumatology, University of Leipzig, Leipzig, Germany	contributor
Ruiz-Rebollo, ML	Digestive Diseases Department Clinical University Hospital of Valladolid, Valladolid, Spain	contributor
Rumbus, Z	Institute for Translational Medicine, Medical School, University of Pecs, Pecs, Hungary	contributor
Rusakov, DA	UCL Queen Square Institute of Neurology, University College London, London, UK	contributor
Rygiel, AM	Department of Medical Genetics, Institute of Mother and Child, Warsaw, Poland; Institute of Computer Science, Warsaw University of Technology, Warsaw, Poland	contributor
Sá, M	Unidade HBP, Serviço de Cirurgia Geral, Centro Hospitalar Tondela-Viseu, Viseu, Portugal	contributor
Sagau, ED	"Octavin Fodor" Institute of Gastroenterology and Hepartology, "Iuliu Hatieganu" University of Medicine and Pharmacy, Cluj Napoca, Romania	contributor
Sahin-Tóth, M	Department of Surgery, University of California Los Angeles, Los Angeles, CA, USA	contributor
Saizu, IA	Clinical Institute Fundeni, Bucharest, Romania	contributor
Salas, IM	Department of Gastroenterology, University Hospital Santa María - University Hospital Arnau de Vilanova, Lerida, Spain	contributor
Sallinen, V	Department of Transplantation and Liver Surgery, Helsinki University Hospital and University of Helsinki, Helsinki, Finland	contributor
Sandblom, G	Department of Clinical Science and Education Södersjukhuset, Karolinska Institutet, Department of Surgery, Södersjukhuset, Stockholm, Sweden	contributor
Sandru, V	mergency Hospital of Bucharest, Carol Davila University of Medicine and Pharmacy Bucharest, Bucharest, Romania	contributor
Santervas, SI	Servicio de Aparato Digestivo Hospital Clínico Universitario Valladolid, Valladolid, Spain	contributor
Sári, R	First Department of Medicine, University of Szeged, Szeged, Hungary	contributor
Sarlós, P	Department of Gastroenterology, First Department of Medicine, Medical School, University of Pécs, Pécs, Hungary	contributor
Saum, K	Else Kröner-Fresenius-Zentrum für Ernährungsmedizin (EKFZ), Technische Universität München (TUM), Freising, Germany	contributor
Schatterny, J	Department of Translational Pulmonology, Translational Lung Research Center Heidelberg, German Center for Lung Research (DZL), University of Heidelberg, Heidelberg, Germany	contributor
Schmidt, F	Department of Pediatrics, University of Halle-Wittenberg, Halle (Saale), Germany	contributor
Schmidt, H	Department for Transplant Medicine, University Hospital Münster, Albert-Schweitzer-Campus 1, Münster, Germany	contributor
Schneider, A	Department of Gastroenterology and Hepatology, Klinikum Bad Hersfeld, Bad Hersfeld, Germany	contributor
Schnúr, A	Department of Medicine, University of Szeged, Szeged, Hungary	contributor
Schuelke, M	Department of Neuropediatrics, Charité, Campus Virchow-Klinikum, Berlin, Germany	contributor
Schulz, HU	Department of Surgery, Otto-von-Guericke University Magdeburg, Magdeburg, Germany	contributor

Name	Institute	Role
Seidler, Ursula	Department of Gastroenterology, Hepatology and Endocrinology, Hannover Medical School, Hannover, Germany	contributor
Semenenko, IA	Sechenov University, Moscow, Russia	contributor
Sendler, M	Department of Internal Medicine A, Universitätsmedizin Greifswald, Greifswald, Germany	contributor
Sendstad, IH	Department of Medicine, University of Szeged, Szeged, Hungary	contributor
Shalbuyeva, N	Veterans Affairs Greater Los Angeles Healthcare System and University of California, Los Angeles, USA	contributor
Sheel, ARG	Department of Clinical Cancer Medicine, Institute of Translational Medicine, University of Liverpool, Liverpool, UK	contributor
Shimosegawa, T	Division of Gastroenterology, Tohoku University Graduate School of Medicine, Sendai, Miyagi, Japan	contributor
Shirinskaya, AV	Department of Surgery and Urology, Omsk State Medical University, Omsk, Russia	contributor
Shirinskaya, N	Omsk State Medical Information-Analytical Centre, Omsk State Medical University, Omsk State Clinical Emergency Hospital #2, Omsk, Russia	contributor
Simanaitis, V	Department of Surgery, Lithuanian University of Health Sciences, Kaunas, Lithuania	contributor
Sinervirta, R	Department of Biotechnology and Molecular Medicine, A. I. Virtanen Institute for Molecular Sciences, Biocenter Kuopio, University of Kuopio, Kuopio, Finland	contributor
Singh, A	Department of Gastroenterology, Hepatology and Endocrinology, Hannover Medical School, Hannover, Germany	contributor
Singh, VP	Department of Medicine, Mayo Clinic, Scottsdale, AZ, USA	contributor
Sipos, Z	Institute for Translational Medicine, Medical School, University of Pécs, Pécs, Hungary	contributor
Siska, A	Department of Clinical Chemistry, University of Szeged, Szeged, Hungary	contributor
Sobczynska-Tomaszewska, A	MedGen Health Care Centre, Warsaw, Poland	contributor
Soltes, M	1st Department of Surgery, University Hospital of L. Pasteur, Kosice, Slovakia	contributor
Solymár, M	Institute for Translational Medicine, Medical School, University of Pécs, Pécs, Hungary	contributor
Somorácz, Á	Second Department of Pathology, Semmelweis University, Budapest, Hungary	contributor
Song, K	"Carol Davila" University of Medicine and Pharmacy, Bucharest, Romania	contributor
Soós, A	Department of Medicine, University of Szeged, Szeged, Hungary	contributor
Söti, D	Center for Translational Medicine, Semmelweis University, Budapest, Hungary	contributor
Stecher, SS	Department of Medicine II, University Hospital, LMU Munich, Germany	contributor
Steigenberger, S	Else Kröner-Fresenius-Zentrum für Ernährungsmedizin (EKFZ), Technische Universität München (TUM), Freising, Germany	contributor
Stimac, D	Clinical Hospital Center Rijeka, Rijeka, Croatia	contributor
Stubnya, B	First Department of Pediatrics, Semmelweis University, Budapest, Hungary	contributor
Stumvoll, M	Department for Internal Medicine, Neurology and Dermatology, Division of Endocrinology, University of Leipzig, Leipzig, Germany	contributor
Suceveanu, AI	Faculty of Medicine, Ovidius University of Constanta, County, Emergency, and Clinical Hospital of Constanta, Constanta, Romania	contributor
Sud, A	Liverpool Pancreatitis Research Group, University of Liverpool and the Royal Liverpool and Broadgreen University Hospital Trust, Liverpool, UK	contributor
Susak, Y	Department of Surgery, Bogomolets National Medical University, Kyiv, Ukraine	contributor
Susánszki, P	Department of Medicine, University of Szeged, Szeged, Hungary	contributor
Sutton, R	University of Liverpool, Liverpool University Hospitals NHS Foundation Trust, Liverpool, UK	contributor
Sümegi, J	Borsod-Abaúj-Zemplén County Hospital and University Teaching Hospital, Miskolc, Hungary	contributor
Svébis, MM	Department of Internal Medicine, Semmelweis University of Medicine, Budapest, Hungary	contributor
Sz Varga, I	Biological Isotope Laboratory, Attila József University, Szeged, Hungary	contributor
Szabó, A	Institute for Translational Medicine, Szentágothai Research Centre, Medical School, University of Pécs, Pécs, Hungary	contributor
Szabó, B	Centre for Translational Medicine, Semmelweis University, 1085, Budapest, Hungary	contributor
Szabó, FK	Division of Gastroenterology and Nutrition, Children's Hospital of Richmond, Virginia Commonwealth University, Richmond, VA, USA	contributor
Szabó, I	Department of Gastroenterology, First Department of Medicine, Medical School, University of Pécs, Pécs, Hungary	contributor
Szabó, L	Institute for Translational Medicine, Szentágothai Research Centre, Medical School, University of Pécs, Pécs, Hungary	contributor
Szabó, S	Hepatology and Nutrition, Cincinnati Children's Hospital Division of Pediatric Gastroenterology, Cincinnati, Ohio, USA	contributor
Szabó, V	MTA-SZTE Momentum Epithelial Cell Signaling and Secretion Research Group, University of Szeged, Szeged, Hungary	contributor
Szabolcs, A	First Department of Medicine, University of Szeged, Szeged, Hungary	contributor
Szadai, L	Department of Dermatology and Allergology, University of Szeged, Szeged, Hungary	contributor
Szakács, Z	Institute for Translational Medicine, Medical School, University of Pécs, Pécs, Hungary	contributor
Szakó, L	Institute for Translational Medicine, Medical School, University of Pécs, Pécs, Hungary	contributor

Name	Institute	Role
Szalai, EÁ	Department of Restorative Dentistry and Endodontics, Semmelweis University, Budapest, Hungary	contributor
Szatmary, P	University of Liverpool, Liverpool University Hospitals NHS Foundation Trust, Liverpool, UK	contributor
Szederkényi, E	Department of Surgery, University of Szeged, Szeged, Hungary	contributor
Székely, CA	First Department of Medicine, University of Szeged, Szeged, Hungary	contributor
Szekeres, A	Department of Microbiology, University of Szeged, Szeged, Hungary	contributor
Szemes, K	Division of Gastroenterology, First Department of Medicine, Medical School, University of Pécs, Pécs, Hungary	contributor
Szénási, G	School of Medicine, Institute of Translational Medicine, Semmelweis University, Budapest, Hungary	contributor
Szentkereszty, Zs	Department for Surgery, University of Debrecen, Debrecen, Hungary	contributor
Szepes, A	Department of Gastroenterology, Bács-Kiskun County Hospital, Kecskemét, Hungary	contributor
Szepes, Z	Department of Medicine, University of Szeged, Szeged, Hungary	contributor
Szilvássy, Z	Department of Pharmacology, Medical University of Debrecen, Debrecen, Hungary	contributor
Szmola, R	Department of Interventional Gastroenterology, National Institute of Oncology, Budapest, Hungary	contributor
Szögi, T	Department of Pathology, University of Szeged, Szeged, Hungary	contributor
Szőllősiné Varga, I	Biological Isotope Laboratory, University of Szeged, Szeged, Hungary	contributor
Szücs, Á	First Department of Surgery, Semmelweis University, Budapest, Hungary	contributor
Szűcs, E	Institute of Biochemistry, Biological Research Center, 6726 Szeged, Hungary	contributor
Tajti, M	First Department of Medicine, University of Szeged Faculty of Medicine, Szeged, Hungary	contributor
Takács, T.	First Department of Medicine, University of Szeged, Szeged, Hungary	contributor
Takaori, K	Department of Surgery, Kyoto University Graduate School of Medicine, Kyoto, Japan	contributor
Takáts, A	Institute for Translational Medicine, Medical School, University of Pécs, Pécs, Hungary	contributor
Tantau, AI	Gastroenterology Department, 4th Medical Clinic, "Iuliu Hatieganu" University of Medicine and Pharmacy, Cluj Napoca, Romania	contributor
Tantau, M	Department of Gastroenterology, Iuliu Hatieganu University of Medicine and Pharmacy, Prof. Octavian Fodor Institute of Gastroenterology and Hepatology, Cluj-Napoca, Romania	contributor
Tantau, VM	"Octavin Fodor" Institute of Gastroenterology and Hepartology, "Iuliu Hatieganu" University of Medicine and Pharmacy, Cluj Napoca, Romania	contributor
Tari, E	Institute of Pancreatic Diseases, Semmelweis University, Budapest, Hungary	contributor
Tarján, D	Division of Pancreatic Disorders, Heart and Vascular Centre, Semmelweis University, Budapest, Hungary	contributor
Tárnok, A	Department of Paediatrics, Medical School, University of Pécs, Pécs, Hungary	contributor
Tcaciuc, E	Department of Gastroenterology, Nicolae Testemitanu State University of Medicine and Pharmacy, Chisinau, Republic of Moldova	contributor
Tebbing, J	Else Kröner-Fresenius-Zentrum für Ernährungsmedizin (EKFZ), Technische Universität München (TUM), Freising, Germany	contributor
Teich, N	Practice for Digestive and Metabolic Diseases, Leipzig, Germany	contributor
Tél, B	Department of Pediatrics, Semmelweis University, Budapest, Hungary	contributor
Tenk, J	Institute for Translational Medicine, Medical School, University of Pécs, Pécs, Hungary	contributor
Tészás, A	Department of Paediatrics, University of Pécs Clinical Centre, Pécs, Hungary	contributor
Teutsch, B	Institute for Translational Medicine, Medical School, University of Pécs, Pecs, Hungary	contributor
Tihanyi, B	Department for Surgery, Hungarian Defence Forces - Medical Centre, Budapest, Hungary	contributor
Tinusz, B	Institute for Translational Medicine, Medical School, University of Pécs, Pécs, Hungary	contributor
Tiszlavicz, L	Department of Pathology, University of Szeged, Szeged, Hungary	contributor
Tiuliukin, IO	O.O.Bogomolets National Medical University, Kiev, Ukraine	contributor
Tlili, A	Mohamed Ben Sassi Hospital, Gabes, Tunisia	contributor
Tocia, C	Faculty of Medicine, Ovidius University of Constanta, Constanta, Romania	contributor
Tod, P	Centre for Translational Medicine, Semmelweis University, Budapest, Hungary	contributor
Tóháti, R	Semmelweis University, Budapest, Hungary	contributor
Tokarev, MV	Sklifosovsky Institute for Clinical Medicine, Sechenov First Moscow State Medical University, Moscow, Russia	contributor
Tokodi, I	Szent György University Teaching Hospital of Fejér County, Székesfehérvár, Hungary	contributor
Tornai, T	Division of Pancreatic Disorders, Heart and Vascular Centre, Semmelweis University, Budapest, Hungary	contributor
Tóth, A	Pediatric Nutritional Medicine & Else Kröner-Fresenius-Centre for Nutritional Medicine (EKFZ), Technical University Munich (TUM), Freising, Germany	contributor
Tóth, A	Department of Microbiology, University of Szeged, Szeged, Hungary	contributor

Name	Institute	Role
Tóth, B	Department of Pathophysiology, University of Szeged,Szeged, Hungary	contributor
Tóth, E	Department of Medicine, University of Szeged, Szeged, Hungary	contributor
Tóth, S	Division of Pancreatic Disorders, Heart and Vascular Centre, Semmelweis University, Budapest, Hungary	contributor
Tozlu, M	Department of Gastroenterology, Faculty of Medicine, Sakarya University, Sakarya, Turkey	contributor
Török, I	County Emergency Clinical Hospital of Târgu Mures - Gastroenterology Clinic and University of Medicine, Pharmacy, Sciences and Technology "George Emil Palade", Targu Mures, Romania	contributor
Tretter, L	Department of Medical Biochemistry, Semmelweis University, Budapest, Hungary	contributor
Tubak, V	Creative Laboratory Ltd., Szeged, Hungary	contributor
Tulassay, Zs	Second Department of Medicine, Semmelweis University, Budapest, Hungary	contributor
Turcan, S	Department of Gastroenterology, Nicolae Testemitanu State University of Medicine and Pharmacy, Chisinau, Republic of Moldova	contributor
Tüzün Ince, A	Hospital of Bezmialem Vakif University, School of Medicine, Istanbul, Turkey	contributor
Uc, A	Division of Pediatric Gastroenterology, Stead Family Department of Pediatrics, University of Iowa Carver College of Medicine, Iowa City, IA, USA	contributor
Unger, LS	Pediatric Nutritional Medicine & Else Kröner-Fresenius-Centre for Nutritional Medicine (EKFZ), Technical University Munich (TUM), Freising, Germany	contributor
Urbán, O	Division of Pancreatic Disorders, Heart and Vascular Centre, Semmelweis University, Budapest, Hungary	contributor
Vág, J	Department of Conservative Dentistry, Semmelweis University, Budapest, Hungary	contributor
Vajda, Á	First Department of Medicine, University of Szeged, Szeged, Hungary	contributor
Váncsa, S	Institute for Translational Medicine, Medical School, University of Pécs, Pécs, Hungary	contributor
Varabei, AV	Belarusian Medical Academy of Postgraduate Education, Minsk, Belarus	contributor
Váradi, A	Department of Metagenomics, University of Debrecen, Debrecen, Hungary	contributor
Varga, Á	Department of Medicine, University of Szeged, Szeged, Hungary	contributor
Varga, Cs	Department of Emergency Medicine, Semmelweis University, Budapest, Hungary	contributor
Varga, G	Institute of Surgical Research, University of Szeged, Szeged, Hungary	contributor
Varga, M	Department of Gastroenterology, BMKK Dr. Rethy Pál Hospital, Békéscsaba, Hungary	contributor
Varga, SI	Department of Biochemistry and Molecular Biology, University of Szeged, Szeged, Hungary	contributor
Varga,M	Dr. Réthy Pál Hospital, Békéscsaba, Hungary	contributor
Varga-Müller, M	Institute for Translational Medicine, University of Pécs, Pécs, Hungary	contributor
Varghese, T	Department of Gastroenterology, Medical College Hospital, Calicut, India	contributor
Varjú, P	Institute for Translational Medicine, Medical School, University of Pécs, Pécs, Hungary	contributor
Varró, A	Department of Pharmacology and Pharmacotherapy, University of Szeged, Szeged, Hungary	contributor
Vécsei, L	Department of Neurology, Interdisciplinary Excellence Centre, University of Szeged, Szeged, Hungary	contributor
Végh, E	First Department of Medicine, University of Szeged, Szeged, Hungary	contributor
Végh, ET	First Department of Medicine, University of Szeged, Szeged, Hungary	contributor
Veligotsky, N	Department Thoraco-abdominal Surgery Kharkov Medical Academy Postgraduate Education, Kharkov, Ukraine	contributor
Venglovecz, V	Department of Pharmacology and Pharmacotherapy, University of Szeged, Szeged, Hungary	contributor
Verbói, M	Institute for Translational Medicine, Medical School, University of Pécs, Pécs, Hungary	contributor
Veréb, Z	Doctoral School of Clinical Medicine, University of Szeged, Szeged, Hungary	contributor
Vereczkei, A	Department of Surgery, Medical School, University of Pécs, Pécs, Hungary	contributor
Vereczkei, Z	Institute for Translational Medicine, Medical School, University of Pécs, Pécs, Hungary	contributor
Veres, DS	Department of Biophysics and Radiation Biology, Semmelweis University, Budapest, Hungary	contributor
Veres, G	Pediatric Institute-Clinic, Medical School, University of Debrecen, Debrecen, Hungary	contributor
Verkhratsky, A	Faculty of Biology, Medicine and Health, University of Manchester, Manchester, UK	contributor
Verzár, Z	Department of Emergency Medicine, University of Pécs School of Medicine, Pécs, Hungary	contributor
Vigh, É	Department of Radiology, Medical School, University of Pécs, Pécs, Hungary	contributor
Vincze, Á	Division of Gastroenterology, First Department of Medicine, Medical School, University of Pécs, Pécs, Hungary	contributor
Vincze, PA	Department of Pharmaceutics and Central Clinical Pharmacy, University of Pécs, Pécs, Hungary	contributor
Vinkó, Z	Institute for Translational Medicine, Szentágothai Research Centre, Medical School, University of Pécs, Pécs, Hungary	contributor
Vitális, Z	Department of Gastroenterology, Institute of Internal Medicine, Faculty of Medicine, University of Debrecen, Debrecen, Hungary	contributor

Name	Institute	Role
Vornhuelz, M	LMU University Hospital, LMU Munich, Munich, Germany	contributor
Vörhendi, N	Institute for Translational Medicine, Medical School, University of Pécs, 7623, Pecs, Hungary	contributor
Vranić, L	Department of Gastroenterology, Clinical Hospital Center Rijeka, University of Rijeka, Rijeka, Croatia	contributor
Wauchope, E	Liverpool University Hospitals NHS Foundation Trust, Liverpool, UK	contributor
Wedrychowicz, A	Department of Pediatrics, Gastroenterology and Nutrition, Faculty of Medicine, Jagiellonian University Medical College, Krakow, Poland	contributor
Whitcomb, DC	Division of Gastroenterology, Hepatology and Nutrition, Department of Medicine, University of Pittsburgh, Pittsburgh, PA, USA	contributor
Wiedenmann, B	Department of Internal Medicine, Division of Hepatology and Gastroenterology, Charité, Campus Virchow-Klinikum, Berlin, Germany	contributor
Wilschanski, M	Hadassah Hebrew University Medical Center, Jerusalem, Israel	contributor
Witt, H	Pediatric Nutritional Medicine & Else Kröner-Fresenius-Centre for Nutritional Medicine (EKFZ), Technical University Munich (TUM), Munich, Germany	contributor
Witt, U	Department of Surgery, TUM, Munich, Germany	contributor
Wittmann, T	First Department of Medicine, University of Szeged, Szeged, Hungary	contributor
Wu, D	Department of Gastroenterology, Peking Union Medical College Hospital, Beijing, China	contributor
Xia, L	Department of Gastroenterology, First Affiliated Hospital of Nanchang University, Nanchang, China	contributor
Xia, Q	Department of Integrated Traditional Chinese and Western Medicine, Sichuan Provincial Pancreatitis Center and West China-Liverpool Biomedical Research Center, West China Hospital, Sichuan University, Cl	contributor
Xue, P	Department of Integrated Traditional Chinese and Western Medicine, Shangjin Hospital, West China Medical School of Sichuan University, Chengdu, China	contributor
Yadav, D	Division of Gastroenterology, Hepatology and Nutrition, Department of Medicine, University of Pittsburgh, Pittsburgh, PA, USA	contributor
Yaroslav, MS	Department of Surgery With a Course of Emergency and Vascular Surgery, Bogomolet National Medical University, Kiev, Ukraine	contributor
Yeshy, V	Department of Surgery, Belarusian Medical Academy Postgraduate Education, Minsk, Belarus	contributor
Zaccolo, M	Department of Physiology, Anatomy and Genetics, Oxford University, Oxford, UK	contributor
Zádori, N	Institute for Translational Medicine, Medical School, University of Pécs, Pécs, Hungary	contributor
Zahariev, OJ	Institute of Pancreatic Diseases, Semmelweis University, Budapest, Hungary	contributor
Zankl, F	Pediatric Nutritional Medicine & Else Kröner-Fresenius-Centre for Nutritional Medicine (EKFZ), Technical University Munich (TUM), Freising, Germany	contributor
Zatorski, H	Department of Digestive Tract Diseases, Medical University of Lodz, Lodz, Poland	contributor
Závogyán, N	First Department of Medicine, University of Szeged, Szeged, Hungary	contributor
Zemplényi, A	Faculty of Pharmacy, Division of Pharmacoeconomics, University of Pécs, Pécs, Hungary	contributor
Zhou-Suckow, Z	Department of Translational Pulmonology, Translational Lung Research Center Heidelberg, German Center for Lung Research (DZL), University of Heidelberg, Heidelberg, Germany	contributor
Zhu, Q	Department of Gastroenterology, Clinical Medical College, Yangzhou University, Yangzhou, Jiangsu, China	contributor
Zhu, Y	Department of Gastroenterology, First Affiliated Hospital of Nanchang University, Nanchang, China	contributor
Zimmer, K-P	Department of Pediatrics, Justus-Liebig-Universität, Gießen, Germany	contributor
Zolcsák, Á	Department of Biophysics and Radiation Biology, Semmelweis University, Budapest, Hungary	contributor
Zolotov, AN	Department of Pathophysiology, Clinical Pathophysiology, Omsk State Medical University, Omsk, Russia	contributor
Zubek, L	Department of Anesthesiology and Intensive Therapy, Semmelweis University, Budapest, Hungary	contributor
Zvara, Á	Laboratory of Functional Genomics, Biological Research Centre, Hungarian Academy of Sciences, Szeged, Hungary	contributor
Zsoldos, F	Heim Pál Children’s Hospital, Budapest, Hungary	contributor
Zsóri, G	First Department of Medicine, University of Szeged Faculty of Medicine, Szeged, Hungary	contributor

Figure S1. Number of abstracts by countries at the EPC Meeting in 2019, Bergen, Norway

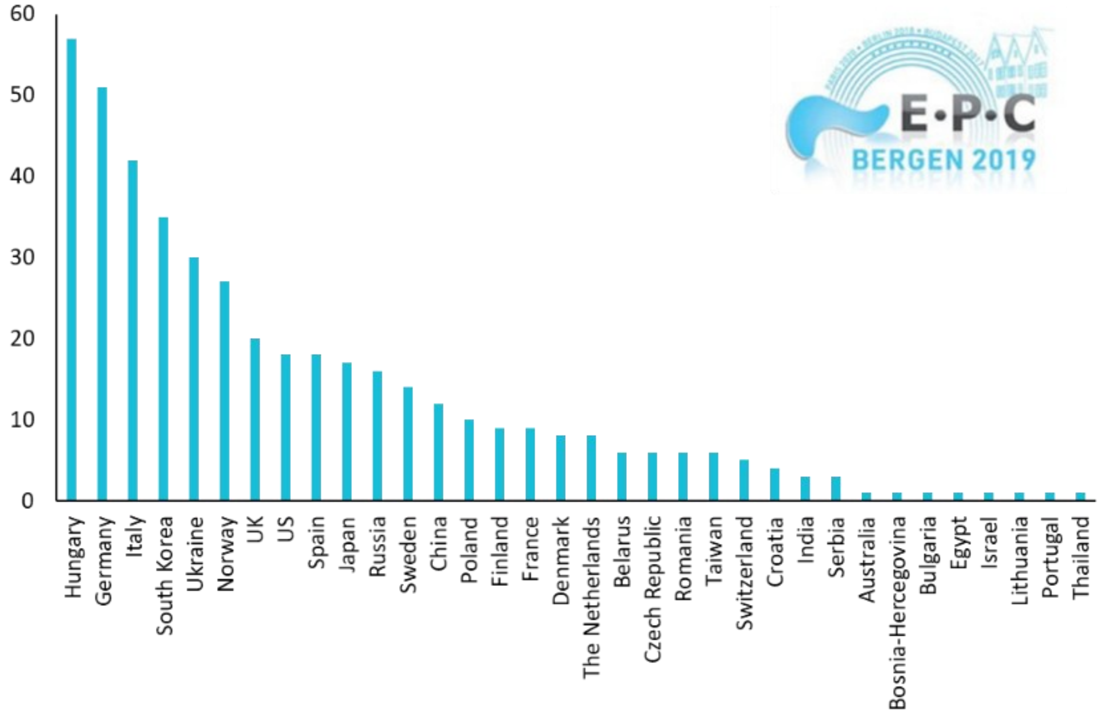

Table S1. Quality evaluation of HPSG cohort analyses

Study	Selection	Comparability	Outcome	NOS score	Quality
Párniczky et al., 2016	★★★★	★★	★★★	9	Good
Lakatos et al., 2016	★★★★	★★	★★★	9	Good
Szücs et al., 2017	★★★★	★	★★	7	Good
Gódi et al., 2018	★★★★	★	★★★	8	Good
Meczker et al., 2019	★★	★	★★★	6	Fair
Szakács et al., 2019	★★★★	★★	★★★	9	Good
Halász et al., 2019	★★★★	★	★★★	8	Good
Párniczky et al., 2019	★★★★	★	★★★	8	Good
Farkas et al., 2019	★★★★	★	★★★	8	Good
Szentesi et al., 2019	★★★★	★★	★★★	9	Good
Huang et al., 2019	★★★★	★★	★★★	9	Good
Zádori et al., 2020	★★★★	★	★★★	8	Good
Mosztbacher et al., 2020	★★★★	★	★★★	8	Good
Hágendorn et al., 2020	★★★★	★	★★★	8	Good
Meczker et al., 2020	★★	★	★★★	6	Fair
Demcsák et al., 2020	★★★★	★★	★★★	9	Good
Hegyi et al., 2021	★★★★	★★	★★★	9	Good
Erőss et al., 2021	★★★★	★★	★★★	9	Good
Szakó et al., 2021	★★★★	★	★★★	8	Good
Nagy et al., 2021	★★★★	★★	★★★	9	Good
Tod et al., 2021	★★★★	★	★★★	8	Good
Ocskay et al., 2021	★★★	★★	★★★	8	Good
Moran et al., 2021	★★★★	★★	★★★	9	Good
Szentesi et al., 2022	★★★★	★	★★★	8	Good
Juhász et al., 2022	★★★★	★	★★★	8	Good
Kiss et al., 2022	★★★★	★★	★★★	9	Good
Dohos et al., 2022	★★★★	★	★★★	8	Good
Földi et al., 2022	★★★★	★	★★★	8	Good
Váncsa et al., 2023	★★★★	★★	★★★	9	Good
Juhász et al., 2023	★★★★	★	★★★	8	Good
Czapári et al., 2023	★★★★	★★	★★	8	Good

Table S2. Evidence-based guidelines by the HPSG

**EVIDENCE-BASED GUIDELINES - HPSG AS DEVELOPER**			
[**Pediatric pancreatitis**. Evidence based management guidelines of the Hungarian Pancreatic Study Group]	Orvosi Hetilap	pediatric pancreatitis	2015
[**Pancreatic cancer**. Evidence based management guidelines of the Hungarian Pancreatic Study Group]	Orvosi Hetilap	pancreatic cancer	2015
[**Autoimmune pancreatitis**. Evidence based management guidelines of the Hungarian Pancreatic Study Group]	Orvosi Hetilap	autoimmun pancreatitis	2015
[**Chronic pancreatitis**. Evidence based management guidelines of the Hungarian Pancreatic Study Group]	Orvosi Hetilap	chronic pancreatitis	2015
[**Acute pancreatitis**. Evidence-based practice guidelines, prepared by the Hungarian Pancreatic Study Group]	Orvosi Hetilap	acute pancreatitis	2015
EPC/HPSG evidence-based guidelines for the management of **pediatric pancreatitis**	Pancreatology	pediatric pancreatitis	2018
**EVIDENCE-BASED GUIDELINES - HPSG AS CONTRIBUTOR**			
United European Gastroenterology evidence-based guidelines for the **diagnosis and therapy of chronic pancreatitis** (HaPanEU)	UEG Journal	chronic pancreatitis	2017
Recommendations from the United European Gastroenterology evidence-based guidelines for the **diagnosis and therapy of chronic pancreatitis**	Pancreatology	chronic pancreatitis	2018
International consensus statements on **early chronic pancreatitis**. Recommendations from the working group for the international consensus guidelines for chronic pancreatitis in collaboration with the International Association of Pancreatology, American Pancreatic Association, Japan Pancreas Society, PancreasFest Working Group and European Pancreatic Club	Pancreatology	chronic pancreatitis	2018
European evidence-based guidelines on **pancreatic cystic neoplasms**	Gut	pancreatic cancer	2018
International consensus guidelines on **surveillance for pancreatic cancer in chronic pancreatitis**. Recommendations from the working group for the international consensus guidelines for chronic pancreatitis in collaboration with the International Association of Pancreatology, the American Pancreatic Association, the Japan Pancreas Society, and European Pancreatic Club	Pancreatology	chronic pancreatitis	2020
International consensus guidelines for **risk factors in chronic pancreatitis**. Recommendations from the working group for the international consensus guidelines for chronic pancreatitis in collaboration with the International Association of Pancreatology, the American Pancreatic Association, the Japan Pancreas Society, and European Pancreatic Club	Pancreatology	chronic pancreatitis	2020

Table S3. The list of HPSG publications

Title	Year	D1-Q4	IF	Disease	Type	Journal	doi
Intraductal acidosis in acute biliary pancreatitis	2013	Q1	2.504	AP	Basic	Pancreatology	doi: 10.1016/j.pan.2013.05.011.
The exocrine pancreas: the acinar-ductal tango in ph	2013	D1	3.9	AP	Review	Rev Physiol Biochem Pharmacol	doi: 10.1007/112_2013_14.
Robust autoactivation, chymotrypsin C independen	2013	Q1	3.986	CP	Registry-Genetics	The FEBS Journal	doi: 10.1111/febs.12292.
Central role of mitochondrial injury in the pathogen	2013	Q1	4.251	AP	Review Basic	Acta Physiol (Oxf)	doi: 10.1111/apha.12037.
Variants in CPA1 are strongly associated with early c	2013	D1	29.648	CP	Basic	Nature Genetics	doi: 10.1038/ng.2730.
Pre-study protocol MagPEP: a multicentre randomiz	2013	Q1	2.113	AP	Pre-study protocol	BMC Gastroenterology	doi: 10.1186/1471-230X-13-11.
Calcium signaling in pancreatic ductal epithelial cells	2014	Q1	3.513	AP	Review	Cell Calcium	doi: 10.1016/j.ceca.2014.02.004.
The role of pancreatic ductal secretion in protection	2014	Q1	6.312	AP	Basic	Critical Care Medicine	doi: 10.1097/CCM.0000000000000101.
Recent advances in the investigation of pancreatic ir	2014	Q1	3.676	AP	Review Basic	Laboratory Investigation	doi: 10.1038/labinvest.2013.143.
Ethanol and its non-oxidative metabolites profound	2014	Q1	4.101	AP	Basic	Pflugers Arch	doi: 10.1007/s00424-013-1333-x.
Chronic inflammation in the pancreas and salivary g	2014	Q1	3.452	CP	Review -Basic	Curr Pharm Des	doi: 10.2174/13816128113199990415.
Functional effects of 13 rare PRSS1 variants presume	2014	D1	14.66	CP	Registry-Genetics	Gut	doi: 10.1136/gutjnl-2012-304331.
[Pediatric pancreatitis. Evidence based managemen	2015	Q4	0.349	PP	Guideline	Orvosi Hetilap	doi: 10.1556/OH.2015.30062.
Genetic analysis of the bicarbonate secreting anion	2015	Q1	2.406	CP	Registry-Genetics	Pancreatology	doi: 10.1016/j.pan.2015.08.008.
[Pancreatic cancer. Evidence based management gu	2015	Q4	0.349	PC	Guideline	Orvosi Hetilap	doi: 10.1556/OH.2015.30063.
[Autoimmune pancreatitis. Evidence based manage	2015	Q4	0.349	AIP	Guideline	Orvosi Hetilap	doi: 10.1556/OH.2015.30061.
[Chronic pancreatitis. Evidence based management	2015	Q4	0.349	CP	Guideline	Orvosi Hetilap	doi: 10.1556/OH.2015.30060.
[Acute pancreatitis. Evidence-based practice guideli	2015	Q4	0.349	AP	Guideline	Orvosi Hetilap	doi: 10.1556/OH.2015.30059.
The Physiology and Pathophysiology of Pancreatic D	2015	Q1	2.738	AP	Basic	Pancreas	doi: 10.1097/MPA.0000000000000421.
Cystic fibrosis-style changes in the early phase of pa	2015	Q2	1.872	AP	Review	Clin Res Hepatol Gastroenterol	doi: 10.1016/j.clinre.2015.05.020.
Breakdown of bioenergetics evoked by mitochondri	2015	Q1	2.406	AP	Review	Pancreatology	doi: 10.1016/j.pan.2015.06.002.
Early Achievable Severity (EASY) index for simple an	2015	Q2	1.891	AP	Pre-study protocol	Journal of Gastrointestinal and L	doi: 10.15403/jgld.2014.1121.242.easy.
Pancreatic epithelial fluid and bicarbonate secretion	2015	D1	14.921	AP	Basic	Gut	doi: 10.1136/gutjnl-2015-309776.
The role of pancreatic ducts in the pathogenesis of a	2015	Q1	2.406	AP	Review	Pancreatology	doi: 10.1016/j.pan.2015.03.010.
Preventive pancreatic stents in the management of a	2015	Q1	2.406	AP	Pre-study protocol	Pancreatology	doi: 10.1016/j.pan.2015.02.007.
New insights into the methodology of L-arginine-ind	2015	Q1	3.057	AP	Basic	PLoS One	doi: 10.1371/journal.pone.0117588.
Alcohol disrupts levels and function of the cystic fibr	2015	D1	18.187	AP	Basic	Gastroenterology	doi: 10.1053/j.gastro.2014.11.002.
Potassium channels in pancreatic duct epithelial cell	2015	Q1	3.654	AP	Review	Pflugers Arch	doi: 10.1007/s00424-014-1585-0.
Bile acids inhibit Na^+/H^+ exchanger and Cl^-/HCO_3^- ex	2015	Q1	3.654	AP	Basic	Pflugers Arch	doi: 10.1007/s00424-014-1560-9.
Complement component 5 mediates development c	2015	D1	18.187	CP	Basic	Gastroenterology	doi: 10.1053/j.gastro.2015.05.012.
Polymorphisms at PRSS1–PRSS2 and CLDN2–MORC4	2015	D1	14.921	CP	Registry-Genetics	Gut	doi: 10.1136/gutjnl-2014-307453.
Pain in the Early Phase of Pediatric Pancreatitis (PINI	2016	Q2	2.088	PP	Pre-study protocol	Digestion	doi: 10.1159/000441352.
Prospective, Multicentre, Nationwide Clinical Data fr	2016	Q1	2.806	AP	Registry-Cohort	PLoS One	doi: 10.1371/journal.pone.0165309.
Analysis of Research Activity in Gastroenterology: Pa	2016	Q2	2.088	AP	Retrospective	PLoS One	doi: 10.1371/journal.pone.0165244.
Meta-Analysis of Early Nutrition: The Benefits of Ent	2016	Q1	3.226	AP	Meta-analysis	International Journal of Molecul	doi: 10.3390/ijms17101691.
Genetic Analysis of Human Chymotrypsin-Like Elasta	2016	Q1	3.226	CP	Registry-Genetics	International Journal of Molecul	doi: 10.3390/ijms17122148.
SPINK1 Promoter Variants in Chronic Pancreatitis	2016	Q1	2.967	CP	Registry-Genetics	Pancreas	doi: 10.1097/MPA.0000000000000412.
A Common CCK-B Receptor Intronic Variant in Pancr	2016	Q1	2.967	PC	Registry-Genetics	Pancreas	doi: 10.1097/MPA.0000000000000539.
Bile as a key aetiological factor of acute but not chro	2016	Q1	4.739	AP	Basic	Journal of Physiology	doi: 10.1113/JP273108.
Ca2+ toxicity and mitochondrial damage in acute pa	2016	D1	5.846	AP	Review	Philos Trans R Soc Lond B Biol Sc	doi: 10.1098/rstb.2015.0425.
Pancreatic Cancer: Multicenter Prospective Data Col	2016	Q2	1.837	PC	Registry-Cohort	Journal of Gastrointestinal and L	doi: 10.15403/jgld.2014.1121.252.pcr.
CFTR: A New Horizon in the Pathomechanism and Tr	2016	D1	4.769	AP	Review	Rev Physiol Biochem Pharmacol	doi: 10.1007/112_2015_5002.
Pathogenic cellular role of the p.L104P human cation	2016	Q1	3.468	CP	Registry-Genetics	Am J Physiol Gastrointest Liver P	doi: 10.1152/ajpgi.00444.2015.
A novel, protective role of ursodeoxycholate in bile-	2016	Q1	3.468	AP	Basic	Am J Physiol Gastrointest Liver P	doi: 10.1152/ajpgi.00317.2015.
Toxic-metabolic risk factors in pediatric pancreatitis:	2016	Q1	2.799	PP	Review	Journal of pediatric gastroenterc	doi: 10.1097/MPG.0000000000001035
Blockade of calcium entry provides a therapeutic wi	2016	Q1	5.182	AP	Comment	The Journal of Physiology	doi: 10.1113/JP271710.
Epithelial anion transport as modulator of chemokir	2016	Q2	3.232	P	Review	Mediators of inflammation	doi: 10.1155/2016/7596531.
Analysis of Pediatric Pancreatitis (APPLE Trial): Pre-S	2017	Q2	2.088	PP	Pre-study protocol	Digestion	doi:10.1159/000441353
Chronic pancreatitis: Multicentre prospective data c	2017	Q1	2.766	CP	Registry-Cohort	PloS One	doi: 10.1371/journal.pone.0171420.
Novel PRSS1 Mutation p.P17T Validates Pathogenic F	2017	D1	10.231	CP	Registry-Genetics	American Journal of Gastroenter	doi: 10.1038/ajg.2017.393.
Smoking and Drinking Synergize in Pancreatitis: Mul	2017	D1	20.773	AP	Comment Basic	Gastroenterology	doi: 10.1053/j.gastro.2017.10.031.
High versus low energy administration in the early p	2017	Q1	2.413	AP	Pre-study protocol	BMJ Open	doi: 10.1136/bmjopen-2017-015874.
Detection of human elastase isoforms by the ScheBo	2017	Q1	3.293	CP	Registry-Genetics	American Journal of Physiology	doi: 10.1152/ajpgi.00060.2017.
United European Gastroenterology evidence-based	2017	Q1	3.477	CP	Guideline	United European Gastroenterolo	doi: 10.1177/2050640616684695.
Restoration of energy level in the early phase of acu	2017	Q1	3.3	PP	Meta-analysis	World Journal of Gastroenterolo	doi: 10.3748/wjg.v23.i6.957.
Misfolding cationic trypsinogen variant p.L104P caus	2017	D1	17.016	CP	CaseGen	Gut	doi: 10.1136/gutjnl-2016-313451.
Efficacy and Safety of FOLFIRINOX in Locally Advance	2017	Q2	1.935	PC	Clinical trial	Pathology & Oncology Research	doi: 10.1007/s12253-016-0176-0.
Genome-wide association study identifies inversion	2018	D1	17.943	CP	Registry-Genetics	Gut	doi: 10.1136/gutjnl-2017-314454.
Centralized care for acute pancreatitis significantly i	2018	Q2	2.063	AP	Registry-Cohort	Journal of Gastrointestinal and L	doi: 10.15403/jgld.2014.1121.272.pan
Recommendations from the United European Gastr	2018	Q1	3.241	CP	Guideline	Pancreatology	doi: 10.1016/j.pan.2018.09.016.
Bidirectional Relationship Between Reduced Blood p	2018	Q2	3.201	AP	Meta-analysis	Frontiers in Physiology	doi: 10.3389/fphys.2018.01360.
Genetic determinants of telomere length and risk of	2018	Q1	5.145	PC	Registry-Genetics	International journal of Cancer	doi: 10.1002/ijc.31928.
The effect of serum triglyceride concentration on th	2018	D1	4.011	AP	Meta-analysis	Scientific Reports	doi: 10.1038/s41598-018-32337-x.
Pancreatitis-Associated Genes and Pancreatic Cance	2018	Q2	2.675	PC	Meta-analysis	Pancreas	doi: 10.1097/MPA.0000000000001145.
Preexisting Diabetes Elevates Risk of Local and Syste	2018	Q2	2.675	AP	Meta-analysis	Pancreas	doi: 10.1097/MPA.0000000000001122.
Guts and Gall: Bile Acids in Regulation of Intestinal E	2018	Q1	24.25	AP	Review Basic	Physiological Reviews	doi: 10.1152/physrev.00054.2017.
The Importance of Aquaporin 1 in Pancreatitis and I	2018	Q2	3.201	AP	Basic	Frontiers in Physiology	doi: 10.3389/fphys.2018.00854
5-ASA induces mild acute pancreatitis. Case report a	2018	Q2	2.063	AP	Case	Journal of Gastrointestinal and L	doi: 10.15403/jgld.2014.1121.272.asa.
Ductal Mucus Obstruction and Reduced Fluid Secret	2018	Q2	3.201	CP	Basic	Frontiers in Physiology	doi: 10.3389/fphys.2018.00632.
Common variants in the CLDN2-MORC4 and PRSS1-F	2018	Q1	3.241	AP	Registry-Genetics	Pancreatology	doi: 10.1016/j.pan.2018.05.486.
International consensus statements on early chronic	2018	Q1	3.241	CP	Guideline	Pancreatology	doi: 10.1016/j.pan.2018.05.008.
Genome-wide meta-analysis identifies five new susc	2018	D1	11.878	PC	Registry-Genetics	Nature Communications	doi: 10.1038/s41467-018-02942-5.
EPC/HPSG evidence-based guidelines for the manag	2018	Q1	3.241	PP	Guideline	Pancreatology	doi: 10.1016/j.pan.2018.01.001.
Mitochondrial Dysfunction, Through Impaired Autop	2018	D1	19.809	AP	Basic	Gastroenterology	doi: 10.1053/j.gastro.2017.10.012.
Accelerating the Drug Delivery Pipeline for Acute an	2018	Q2	2.675	P	Review	Pancreas	doi: 10.1097/MPA.0000000000001176
Necrotic amplification loop in acute pancreatitis: pa	2018	Q1	5.182	AP	Comment	The Journal of Physiology	doi: 10.1113/JP275930.
European evidence-based guidelines on pancreatic c	2018	D1	17.943	PC	Guideline	Gut	doi: 10.1136/gutjnl-2018-316027.
Retrospective Matched-Cohort Analysis of Acute Par	2019	Q1	2.92	AP	Registry-Cohort	Pancreas	doi: 10.1097/MPA.0000000000001297.
Aging and Comorbidities in Acute Pancreatitis II.: A C	2019	Q2	3.367	AP	Registry-Cohort	Frontiers in Physiology	doi: 10.3389/fphys.2018.01776.
Outcomes and timing of endoscopic retrograde chol	2019	Q2	3.57	AP	Registry-Cohort	Digestive and Liver Disease	doi: 10.1016/j.dld.2019.03.018.
Antibiotic therapy in acute pancreatitis: From global	2019	Q1	3.629	AP	Registry-Cohort	Pancreatology	doi: 10.1016/j.pan.2019.04.003.
A Multicenter, International Cohort Analysis of 1435	2019	Q2	3.367	AP	Registry-Cohort	Frontiers in Physiology	doi: 10.3389/fphys.2019.01092.
Multiple Hits in Acute Pancreatitis: Components of M	2019	Q2	3.367	AP	Registry-Cohort	Frontiers in Physiology	doi: 10.3389/fphys.2019.01202.
Common variants in glyoxalase I do not increase chr	2019	Q1	2.74	CP	Registry-Genetics	PLoS One	doi: 10.1371/journal.pone.0222927.
Novel mitochondrial transition pore inhibitor N-met	2019	Q1	4.547	AP	Basic	Journal of Physiology	doi: 10.1113/JP278517.

Supplementary Material

Title	Year	D1-Q4	IF	Disease	Type	Journal	doi
Computed Tomography Severity Index vs. Other Ind	2019	Q2	3.367	AP	Meta-analysis	Frontiers in Physiology	doi: 10.3389/fphys.2019.01002.
Alcohol-dependent effect of PRSS1-PRSS2 haplotype	2019	D1	23.059	CP	Registry-Genetics	Gut	doi: 10.1136/gutjnl-2019-319729.
Observational longitudinal multicentre investigation	2019	Q1	2.496	AP	Pre-study protocol	BMJ Open	doi: 10.1136/bmjopen-2018-025500.
Endoscopic sphincterotoMy for delayIng choLecyste	2019	Q1	2.496	AP	Pre-study protocol	BMJ Open	doi: 10.1136/bmjopen-2018-025551.
Aging and Comorbidities in Acute Pancreatitis I: A M	2019	Q2	3.367	AP	Meta-analysis	Frontiers in Physiology	doi: 10.3389/fphys.2019.00328.
Body-mass index correlates with severity and morta	2019	Q1	3.665	AP	Meta-analysis	World Journal of Gastroenterolo	doi: 10.3748/wjg.v25.i6.729.
Germline BRCA2 K3326X and CHEK2 I157T mutation	2019	Q1	5.145	PC	Registry-Genetics	International Journal of Cancer	doi: 10.1002/ijc.32127.
Evaluation of the Pathogenic Significance of the Nov	2019	Q1	2.92	AP	Case	Pancreas	doi: 10.1097/MPA.0000000000001214.
Genetic variability of the ABCC2 gene and clinical ou	2019	Q1	4.603	PC	Registry-Genetics	Carcinogenesis	doi: 10.1093/carcin/bgz006.
The common truncation variant in pancreatic lipase	2019	Q1	2.776	CP	Registry-Genetics	PloS One	doi: 10.1371/journal.pone.0206869.
Resection of pancreatic cancer in Europe and USA: a	2019	D1	19.819	PC	Registry-Cohort	Gut	doi: 10.1136/gutjnl-2017-314828.
Uncommon appearance of concurrent liver cirrhosis	2019	Without Q		CP	Comment	Digestive and Liver Disease	doi: 10.1016/j.dld.2018.12.023.
Spilanthol inhibits inflammatory transcription factor	2019	Q1	4.556	AP	Basic	International Journal of Molecul	doi: 10.3390/ijms20174308.
Insufficient implementation of the IAP/APA guidelin	2020	Q1	4.623	AP	Registry-Cohort	United European Gastroenterol	doi: 10.1177/2050640620918695.
Hypertriglyceridemia-induced acute pancreatitis: A	2020	Q1	3.996	AP	Registry-Cohort	Pancreatology	doi: 10.1016/j.pan.2020.03.018.
Development of disturbance of consciousness is ass	2020	Q1	3.996	AP	Registry-Cohort	Pancreatology	doi: 10.1016/j.pan.2020.05.009.
Analysis of 1060 Cases of Drug-Induced Acute Pancr	2020	D1	22.682	AP	Registry-Cohort	Gastroenterology	doi: 10.1053/j.gastro.2020.07.016.
Acid suppression therapy, gastrointestinal bleeding	2020	Q1	3.996	AP	Registry-Cohort	Pancreatology	doi: 10.1016/j.pan.2020.08.009.
New Onset of DiabetEs in aSsociation with pancreat	2020	Q1	2.692	PC	Pre-study protocol	BMJ Open	doi: 10.1136/bmjopen-2020-037267.
Bile accelerates carcinogenic processes in pancreati	2020	D1	4.379	PC	Basic	Scientific Reports	doi: 10.1038/s41598-020-79181-6.
Recurrent acute pancreatitis induced by 5-ASA and a	2020	Q1	3.996	AP	Case	Pancreatology	doi: 10.1016/j.pan.2020.10.026.
Insufficient etiological workup of COVID-19-associat	2020	Q1	5.742	AP	Meta-analysis	World Journal of Gastroenterolo	doi: 10.3748/wjg.v26.i40.6270.
Assessment of the course of acute pancreatitis in th	2020	D1	4.379	AP	Meta-analysis	Scientific Reports	doi: 10.1038/s41598-020-74943-8.
Analysis of GPRC6A variants in different pancreatitis	2020	Q1	3.996	CP	Registry-Genetics	Pancreatology	doi: 10.1016/j.pan.2020.08.001.
Fatty Liver Disease and Non-Alcoholic Fatty Liver Dis	2020	Without Q	4.241	AP	Meta-analysis	Journal of Clinical Medicine	doi: 10.3390/jcm9092698.
Lipotoxicity and Cytokine Storm in Severe Acute Pan	2020	D1	22.682	AP	Meta-analysis	Gastroenterology	doi: 10.1053/j.gastro.2020.07.014.
International consensus guidelines on surveillance f	2020	Q1	3.996	CP	Guideline	Pancreatology	doi: 10.1016/j.pan.2020.05.011.
Academia Europaea Position Paper on Translational	2020	Without Q	4.241	AP	Translational medici	Journal of Clinical Medicine	doi: 10.3390/jcm9051532.
International Consensus Guidelines for Risk Factors	2020	Q1	3.996	CP	Guideline	Pancreatology	doi: 10.1016/j.pan.2020.03.014.
Genome-wide association study identifies an early c	2020	D1	7.396	PC	Registry-Genetics	International Journal of Cancer	doi: 10.1002/ijc.33004.
EarLy Elimination of Fatty Acids iN hypertriglyceride	2020	Q1	3.996	AP	Pre-study protocol	Pancreatology	doi: 10.1016/j.pan.2019.12.018.
TRPM2-mediated extracellular Ca 2+ entry promote	2020	Q1	5.182	AP	Basic	Journal of Physiology	doi: 10.1113/JP279047.
LIFEStyle, Prevention and Risk of Acute PaNcreatitis (	2020	Q1	2.692	AP	Pre-study protocol	BMJ Open	doi: 10.1136/bmjopen-2019-029660.
Endoscopic and surgical drainage for pancreatic flui	2020	Q1	3.996	AP	Meta-analysis	Pancreatology	doi: 10.1016/j.pan.2019.10.006.
Mouse pancreatic ductal organoid culture as a relev	2020	Q1	5.662	AP	Basic	Laboratory Investigation	doi: 10.1038/s41374-019-0300-3.
Novel p.K374E variant of CPA1 causes misfolding-in	2020	D1	23.059	CP	CaseGen	Gut	doi: 10.1136/gutjnl-2019-318751.
Inflammatory bowel diseases elevate the risk of dev	2020	Q2	3.327	AP	Meta-analysis	Pancreas	doi: 10.1097/MPA.0000000000001650
PrescrAIP: a pan-European study on current treatme	2020	Q1	5.093	AIP	Retrospective	Frontiers in Medicine	doi: 10.3389/fmed.2020.00408.
Evidence for diagnosis of early chronic pancreatitis a	2021	D1	4.996	AP	Registry-Cohort	Scientific Reports	doi: 10.1038/s41598-020-80532-6.
Metabolic signature might be an option to identify p	2021	D1	31.793	AP	Registry-Cohort	Gut	doi: 10.1136/gutjnl-2021-324206.
Early occurrence of pseudocysts in acute pancreatiti	2021	Q1	3.977	AP	Registry-Cohort	Pancreatology	doi: 10.1016/j.pan.2021.05.007.
Glucose levels show independent and dose-depend	2021	Q1	3.977	AP	Registry-Cohort	Pancreatology	doi: 10.1016/j.pan.2021.06.003.
Initial Renal Function (eGFR) Is a Prognostic Marker	2021	Q1	5.058	AP	Registry-Cohort	Frontiers in Medicine	doi: 10.3389/fmed.2021.671917.
Hypoalbuminemia affects one third of acute pancre	2021	D1	4.996	AP	Registry-Cohort	Scientific Reports	doi: 10.1038/s41598-021-03449-8.
Accelerating the translational medicine cycle: the Ac	2021	D1	87.241	AP	Translational medici	Nature Medicine	doi: 10.1038/s41591-021-01458-8.
Identification of Recessively Inherited Genetic Varia	2021	Q1	5.738	PC	Registry-Genetics	Frontiers in Oncology	doi: 10.3389/fonc.2021.771312.
Endoscopic ultrasound-guided ethanol and radiofre	2021	Q1	4.802	PC	Meta-analysis	Therap Adv Gastroenterol	doi: 10.1177/17562848211042171.
Early infection is an independent risk factor for incre	2021	Q1	3.977	AP	Registry-Cohort	Pancreatology	doi: 10.1016/j.pan.2021.11.003.
Kynurenic Acid and Its Analogue SZR-72 Ameliorate	2021	Q1	8.786	AP	Basic	Frontiers in Immunology	doi: 10.3389/fimmu.2021.702764.
Mislocalization of CFTR expression in acute pancrea	2021	Q1	6.228	AP	Basic	The Journal of Physiology	doi: 10.1113/JP281765.
Association of Genetic Variants Affecting microRNAs	2021	Q2	4.772	PC	Registry-Genetics	Frontiers in Genetics	doi: 10.3389/fgene.2021.693933.
Genetic Polymorphisms Involved in Mitochondrial M	2021	Q1	4.09	PC	Registry-Genetics	Cancer Epidemiol Biomarkers Pr	doi: 10.1158/1055-9965.EPI-21-0353.
Common calcium-sensing receptor (CASR) gene vari	2021	Q1	3.977	CP	Registry-Genetics	Pancreatology	doi: 10.1016/j.pan.2021.08.012.
Combined use of indomethacin and hydration is the	2021	Q1	3.977	AP	Meta-analysis	Pancreatology	doi: 10.1016/j.pan.2021.07.005.
Revisiting the evidence-based management of paed	2021	Q1	3.977	PP	Case	Pancreatology	doi: 10.1016/j.pan.2021.06.008.
Associations between pancreatic expression quantit	2021	Q1	4.741	PC	Registry-Genetics	Carcinogenesis	doi: 10.1093/carcin/bgab057.
Lack of association of CD44-rs353630 and CHI3L2-rs	2021	D1	4.996	PC	Registry-Genetics	Scientific Reports	doi: 10.1038/s41598-021-87130-0.
Genome-wide scan of long noncoding RNA single nu	2021	Q1	7.316	PC	Registry-Genetics	International Journal of Cancer	doi: 10.1002/ijc.33475.
Polygenic and multifactorial scores for pancreatic du	2021	Q1	5.941	PC	Registry-Genetics	Journal of Medical Genetics	doi: 10.1136/jmedgenet-2020-106961.
Design and validation of a patient-reported outcom	2021	D1	31.793	AP	Clinical trial	Gut	doi: 10.1136/gutjnl-2020-320729.
Pancreatitis severity in mice with impaired CFTR fun	2021	Q2	5.295	AP	Basic	J Cell Mol Med	doi: 10.1111/jcmm.16404.
Practice patterns and adherence to nutrition guideli	2021	Q1	3.977	AP	Guideline	Pancreatology	doi: 10.1016/j.pan.2021.01.001
Critical thresholds: key to unlocking the door to the	2021	D1	31.793	AP	Review	Gut	doi: 10.1136/gutjnl-2020-322163
Alcohol consumption and smoking dose-dependent	2022	D1	24.5	AP	Registry-Cohort	Gut	doi: 10.1136/gutjnl-2021-326853
Association of Body Mass Index With Clinical Outcor	2022	D1	13.353	CF	Meta-analysis	JAMA Network Open	doi: 10.1001/jamanetworkopen.2022.0740.
Exosomes as prognostic biomarkers in pancreatic du	2022	D1	10.171	PC	Meta-analysis	Translational Research	doi: 10.1016/j.trsl.2022.01.001.
Impaired regulation of PMCA activity by defective CF	2022	D1	9.207	AP	Basic	Cell Mol Life Sci	doi: 10.1007/s00018-022-04287-1.
EASY-APP: An artificial intelligence model and applic	2022	Q4	10.6	AP	Clinical trial	Clinical and Translational Medici	doi: 10.1002/ctm2.842.
In-Hospital Patient Education Markedly Reduces Alc	2022	D1	5.9	AP	Clinical trial	Nutrients	doi: 10.3390/nu14102131.
Prognostic role of cell-free DNA biomarkers in pancr	2022	Q1	6.625	PC	Meta-analysis	Crit Rev Oncol Hematol	doi: 10.1016/j.critrevonc.2021.103548.
Bile acid- and ethanol-mediated activation of Orai1	2022	Q1	6.228	AP	Basic	Journal of Physiology	doi: 10.1113/JP282203.
Fentanyl but Not Morphine or Buprenorphine Impro	2022	D1	6.208	AP	Basic	International Journal of Molecul	doi: 10.3390/ijms23031192.
Pancreatic family history does not predict disease pr	2022	Q1	3.9	AP	Registry-Cohort	Frontiers in Medicine	doi: 10.3389/fmed.2022.801592
Early prediction of acute necrotizing pancreatitis by	2022	D1	4.6	AP	Registry-Cohort	Scientific Reports	doi: 10.1038/s41598-022-11517-w.
Common variability in oestrogen-related genes and	2022	D1	4.6	PC	Registry-Genetics	Scientific Reports	doi: 10.1038/s41598-022-22973-9.
The combination of ulinastatin and somatostatin re	2022	D1	4.6	AP	Meta-analysis	Scientific Reports	doi: 10.1038/s41598-022-22341-7.
Tricetin Reduces Inflammation and Acinar Cell Injury	2022	Q1	4.757	AP	Basic	Biomedicines	doi: 10.3390/biomedicines10061371.
OGG1 Inhibition Reduces Acinar Cell Injury in a Mou	2022	Q1	4.757	AP	Basic	Biomedicines	doi: 10.3390/biomedicines10102543.
Role of stereotactic body radiation in the enhancem	2022	Q1	4.309	PC	Meta-analysis	Radiat Oncol	doi: 10.1186/s13014-022-02076-5.
The EFFect of dietary fat content on the recurrence	2022	Q1	3.6	AP	Pre-study protocol	Pancreatology	doi: 10.1016/j.pan.2021.10.002.
Variants in the pancreatic CUB and zona pellucida-li	2022	Q1	3.6	CP	Registry-Genetics	Pancreatology	doi: 10.1016/j.pan.2022.04.015.
Diagnosis and treatment of exocrine pancreatic insu	2022	Q1	3.6	CP	Expert opinion surve	Pancreatology	doi: 10.1016/j.pan.2022.03.013.
Inflammatory bowel disease does not alter the clini	2022	Q1	3.6	AP	Registry-Cohort	Pancreatology	doi: 10.1016/j.pan.2022.09.241
Loss-of-function variant in chymotrypsin like elastas	2022	Q1	3.6	CP	Registry-Genetics	Pancreatology	doi: 10.1016/j.pan.2022.06.258.
Risk of chronic pancreatitis in carriers of loss-of-fun	2022	Q1	3.752	CP	Meta-analysis	PLoS One	doi: 10.1371/journal.pone.0268859.

Supplementary Material

Title	Year	D1-Q4	IF	Disease	Type	Journal	doi
Bicarbonate defective CFTR variants increase risk fo	2022	Q1	3.752	CP	Meta-analysis	PLoS One	doi: 10.1371/journal.pone.0276397
The characteristics and prognostic role of acute abd	2022	Q1	3.6	AP	Registry-Cohort	European Journal of Pain	doi: 10.1002/ejp.1885.
Admission risk factors and predictors of moderate o	2022	Q1	3.569	PP	Meta-analysis	Frontiers in Pediatrics	doi: 10.3389/fped.2022.947545.
Recurrent acute pancreatitis prevention by the elimi	2022	Q1	2.9	AP	Pre-study protocol	BMJ Open	doi: 10.1136/bmjopen-2021-050821.
Preoperative Serum Carbohydrate Antigen 19-9 Leve	2022	Q2	2.874	PC	Meta-analysis	Pathol Oncol Res	doi: 10.3389/pore.2022.1610266.
Association between a polymorphic variant in the C	2022	Q1	5.7	PC	Registry-Genetics	International Journal of Cancer	doi: 10.1002/ijc.34383.
Acute Pancreatitis Severity Prediction: It Is Time to U	2022	Q1	3.983	AP	Case series	Journal of Clinical Medicine	doi: 10.3390/jcm12010290.
Polymorphic variants involved in methylation regula	2023	Q1	3.006	PC	Registry-Genetics	BMJ Open	doi: 10.1136/jmg-2022-108910.
Genetic and non-genetic risk factors for early-onset	2023	Q2	4	PC	Registry-Genetics	Digestive and Liver Disease	doi: 10.1016/j.dld.2023.02.023.
Metabolic-associated fatty liver disease is associated	2023	D1	5.8	AP	Registry-Cohort	United European Gastroenterolo	doi: 10.1002/ueg2.12389.
Lactated Ringer's Solution Reduces Severity, Mortali	2023	Q1	4.757	AP	Meta-analysis	Biomedicines	doi: 10.3390/biomedicines11020321.
No evidence for the benefit of PPIs in the treatment	2023	D1	3.8	AP	Meta-analysis	Scientific Reports	doi: 10.1038/s41598-023-29939-5.
Position statement on the definition, incidence, diag	2023	Q1	2.8	CP	Systematic review	Pancreatology	doi: 10.1016/j.pan.2023.01.010.
CFTR-beyond the airways: Recent findings on the ro	2023	Q1	4.233	AP	Review	Journal of Cystic Fibrosis	doi: 10.1016/j.jcf.2022.12.017.
Thiopurines impair the apical plasma membrane ex	2023	Q1	8.189	AP	Basic	Cellular and Molecular Life Scien	doi: 10.1007/s00018-022-04662-y.
The PANcreatic Disease ReseArch (PANDoRA) conso	2023	Q1	5.5	PC	Review	Critical Reviews in Oncology/Hem	doi: 10.1016/j.critrevonc.2023.104020.
Discharge protocol in acute pancreatitis: an internat	2023	D1	3.8	AP	Survey	Scientific Reports	doi: 10.1038/s41598-023-48480-z.
Low molecular weight heparin decreases mortality a	2023	Q1	3.359	AP	Meta-analysis	Frontiers in Medicine	doi: 10.3389/fmed.2023.1241301.
Fatty Pancreas Is a Risk Factor for Pancreatic Cancer	2023	Q1	4.862	PC	Meta-analysis	Cancers	doi: 10.3390/cancers15194876.
Clinical usefulness of scoring systems to predict seve	2023	D1	5.8	AP	Meta-analysis	Uninted European Gastroentero	doi: 10.1002/ueg2.12464.
A scan of all coding region variants of the human ge	2023	Q1	3.3	PC	Registry-Genetics	Carcinogenesis	doi: 10.1093/carcin/bgad056.
Exploring the Neandertal legacy of pancreatic ductal	2023	D1	4.3	PC	Registry-Genetics	Biol Res	doi: 10.1186/s40659-023-00457-y.
Invalidity of Tokyo guidelines in acute biliary pancre	2023	D1	5.8	AP	Registry-Cohort	Uninted European Gastroentero	doi: 10.1002/ueg2.12402.
Risk of chronic pancreatitis in carriers of the c.180C>	2023	Q1	2.8	CP	Meta-analysis	Pancreatology	doi: 10.1016/j.pan.2023.05.013.
Detailed Characteristics of Post-discharge Mortality	2023	D1	25.7	AP	Registry-Cohort	Gastroenterology	doi: 10.1053/j.gastro.2023.05.028.
The risk of developing splanchnic vein thrombosis in	2024	D1	5.8	AP	Meta-analysis	Uninted European Gastroentero	doi: 10.1002/ueg2.12550.
Role of CFTR in diabetes-induced pancreatic ductal f	2024	Q1	3.286	AP	Basic	Journal of Physiology	
Polymorphisms in transcription factor binding sites a	2024	Q1	3.8	PC	Registry-Genetics	Human genomics	doi: 10.1186/s40246-024-00576-x.
Persistently High Procalcitonin and C-Reactive Protei	2024	D1	5.493	AP	Meta-analysis	International Journal of Molecul	doi: 10.3390/ijms25021273.
Risk factors for diabetes mellitus after acute pancrea	2024	Q1	3.359	AP	Meta-analysis	Frontiers in Medicine	doi: 10.3389/fmed.2023.1257222.
Type 1 Autoimmune Pancreatitis in Europe: Clinical	2024	D1	5.374	AIP	Survey	Clinical Gastroenterology and He	doi: 10.1016/j.cgh.2023.12.010.
Nafamostat Reduces the Incidence of post-ERCP Par	2024	D1	5.22	AP	Meta-analysis	Clin Pharmacol Ther	doi: 10.1002/cpt.3118.
Prevalence of autoimmune pancreatitis in pancreati	2024	Q2	2.595	AIP	Meta-analysis	BMC Gastroenterol	doi:10.1186/s12876-024-03367-9
Chronic liver disease is an important risk factor for v	2024	D1	3.8	AP	Meta-analysis	Scientific Reports	doi:10.1038/s41598-024-66710-w
Potential association between PSCA rs2976395 funct	2024	Q1	5.948	PC	Registry-Genetics	Int J Cancer	doi:10.1002/ijc.35046
Incidence of recurrent and chronic pancreatitis after	2024	Q1	4.171	AP	Meta-analysis	Therap Adv Gastroenterol	doi: 10.1177/17562848241255303.
Heavy metals in cigarette smoke strongly inhibit pan	2024	D1	7.9	CP	Basic+Registry-Coho	Clin Translational Medicine	doi: 10.1002/ctm2.1733.
Psychological intervention improves quality of life in	2024	D1	3.8	PC	Meta-analysis	Scientific Reports	doi: 10.1038/s41598-024-63431-y.
Investigating the influence of taurochenodeoxycholi	2024	Q2	4.49	PC	Basic	J. Biotechnol	doi: 10.1016/j.jbiotec.2024.05.010.
A pleiotropy scan to discover new susceptibility loci	2024	Q1	2.5	PC	Registry-Genetics	Mutagenesis	doi: 10.1093/mutage/geae012.
